# HSA-nanobinders crafted from bioresponsive prodrugs for combined cancer chemoimmunotherapy–an *in vitro* exploration

**DOI:** 10.3389/fchem.2024.1378233

**Published:** 2024-03-27

**Authors:** Matilde Tubertini, Luca Menilli, Celeste Milani, Cecilia Martini, Maria Luisa Navacchia, Marta Nugnes, Manuela Bartolini, Marina Naldi, Daniele Tedesco, Elisa Martella, Andrea Guerrini, Claudia Ferroni, Francesca Moret, Greta Varchi

**Affiliations:** ^1^ Institute for Organic Synthesis and Photoreactivity (ISOF), National Research Council of Italy (CNR), Bologna, Italy; ^2^ Department of Science and High Technology, University of Insubria, Como, Italy; ^3^ Pharmacy Unit, Veneto Institute of Oncology IOV-IRCSS, Padua, Italy; ^4^ Department of Biology (DiBio), University of Padova, Padua, Italy; ^5^ Department of Pharmacy and Biotechnology (FaBiT), University of Bologna, Bologna, Italy

**Keywords:** prodrugs, chemotherapy, IDO1 inhibition, endogenous human serum albumin, carrier-free nanoparticles, truncated evans blue, triple-negative breast cancer

## Abstract

**Introduction:** Triple-negative breast cancer (TNBC) is an aggressive subtype of breast cancer still lacking effective treatment options. Chemotherapy in combination with immunotherapy can restrict tumor progression and repolarize the tumor microenvironment towards an anti-tumor milieu, improving clinical outcome in TNBC patients. The chemotherapeutic drug paclitaxel has been shown to induce immunogenic cell death (ICD), whereas inhibitors of the indoleamine 2,3- dioxygenase 1 (IDO1) enzyme, whose expression is shared in immune regulatory and tumor cells, have been revealed to enhance the anti-tumor immune response. However, poor bioavailability and pharmacokinetics, off-target effects and hurdles in achieving therapeutic drug concentrations at the target tissue often limit the effectiveness of combination therapies.

**Methods:** This work describes the development of novel biomimetic and carrier-free nanobinders (NBs) loaded with both paclitaxel and the IDO1 inhibitor NLG919 in the form of bioresponsive and biomimetic prodrugs. A fine tuning of the preparation conditions allowed to identify NB@5 as the most suitable nanoformulation in terms of reproducibility, stability and *in vitro* effectiveness.

**Results and discussion:** Our data show that NB@5 effectively binds to HSA in cell-free experiments, demonstrating its protective role in the controlled release of drugs and suggesting the potential to exploit the protein as the endogenous vehicle for targeted delivery to the tumor site. Our study successfully proves that the drugs encapsulated within the NBs are preferentially released under the altered redox conditions commonly found in the tumor microenvironment, thereby inducing cell death, promoting ICD, and inhibiting IDO1.

## 1 Introduction

Although cancer therapy has made significant progresses in recent years, certain solid tumors or their most aggressive subtypes still lack effective treatment options. One such example is triple-negative breast cancer (TNBC), accounting for 15%–20% of all breast cancers. TNBC is characterized by the absence of estrogen, progesterone, and human epidermal growth factor 2 receptors, and it is associated with high rates of distant recurrence and reduced overall survival (Yin et al., 2020). In light of the limited efficacy of current treatments, significant efforts have been undertaken in recent years to expand the therapeutic options for TNBC patients (Guan et al., 2021; Bianchini et al., 2022). Over the past decade, a substantial body of evidence has underscored the crucial role of the immune system in shaping the disease course (Loizides and Constantinidou, 2022). Tumor-infiltrating lymphocytes (TILs) are widely acknowledged as reliable indicators of favorable prognosis in both adjuvant and neoadjuvant treatments (Loi et al., 2019; Marra et al., 2019). Alongside TILs, the expression of immune evasion molecules in the tumor microenvironment (TME), like programmed death-ligand 1 (PD-L1) and the indoleamine-2,3-dioxygenase enzyme 1 (IDO1), has been demonstrated to negatively influence TNBC prognosis (Beckers et al., 2016; Keenan and Tolaney, 2020; Alkhayyal et al., 2022). In particular, the cytotoxic killing ability of TILs may be limited by the overexpression of IDO1 due to its interference with immune metabolism (Li et al., 2021; Nel et al., 2022; Zhang et al., 2022). IDO1 catalyzes the first and rate-limiting step of the kynurenine (Kyn) pathway, where the essential amino acid l-tryptophan (Trp) is converted into the immunosuppressive metabolite l-Kyn. The effect of this conversion is two-fold: Trp depletion induces apoptosis in T cells through the action of the mammalian target of rapamycin complex 1 (mTORC1) regulatory protein (Ye et al., 2019), while increased levels of Kyn activate the aryl hydrocarbon receptor (AHR), a transcription factor responsible for the differentiation of effector CD4^+^ cells into T_reg_ cells (Nguyen et al., 2010). Therefore, IDO1 inhibition has been revealed as a promising approach to rewire immune surveillance towards cancer cells (Zhai et al., 2018; Gao et al., 2019; Feng et al., 2020; Girithar et al., 2023).

In parallel, recent studies have demonstrated that specific chemotherapeutic drugs, such as doxorubicin, camptothecin, oxaliplatin and paclitaxel (Ptx) can induce immunogenic cell death (ICD) (Lau et al., 2020; Vanmeerbeek et al., 2020; Zhai et al., 2023). During ICD, dying cancer cells release damage-associated molecular patterns (DAMPs) and danger signals, such as calreticulin (CRT), adenosine 5′-triphosphate (ATP) and the high-mobility group box 1 protein (HMGB1), which collectively recruit phagocytic cells and activate the immune system at the tumor site. Additionally, ICD is characterized by the exposure of tumor antigens on the surface of dying cancer cells, which can be taken up by antigen-presenting cells to stimulate T cell response (Kroemer et al., 2022). ICD is of great interest in cancer immunotherapy, as it can enhance the effectiveness of immune checkpoint inhibitors, such as IDO1 inhibitors, by priming the immune system to recognize and attack cancer cells (Bonaventura et al., 2019; Feng et al., 2020). Therefore, inducing ICD and relieving IDO1 immunosuppression by combining drugs capable of simultaneously reaching the TME in an appropriate ratio is expected to achieve potent antitumor efficacy in TNBC.

Approaching combination therapy by including multiple drugs within a single entity represents a meaningful way to reduce unwanted toxicities, ensure comparable pharmacokinetics and achieve an improved delivery of drugs at the tumor site (Nel et al., 2022). In this context, carrier-free drug delivery systems have gained much attention thanks to their unique advantages over carrier-based ones, such as a higher drug payload and no carrier-induced toxicity (Fu et al., 2022). In addition, exploiting the binding to endogenous human serum albumin (HSA) is a valuable option to achieve tumor targeting and improve the pharmacokinetic profile of drugs and nanosystems (Zhang et al., 2018; Chen et al., 2022; Li et al., 2022). As a major source of energy and amino acids, HSA is actively recruited into the TME allowing enhanced accumulation in tumor tissues compared to normal ones (Hoogenboezem and Duvall, 2018). Hence, endogenous HSA can serve as a valuable carrier for targeted drug delivery to tumors by utilizing albumin-binding moieties, enabling the biomimetic delivery of drugs to tumor cells (Zhang et al., 2017; Rapozzi et al., 2022). Several studies have shown that the attachment of drugs or molecular conjugates to HSA significantly prolongs their circulation time and decreases their clearance from the reticuloendothelial system, thus promoting accumulation and retention at the tumor site (Spada et al., 2021; Fan et al., 2022; Ullah et al., 2023).

Given these considerations, this study introduces a unique and pioneering biomimetic nanosystem that encompasses two primary breakthrough components: the design and synthesis of novel non-covalent HSA-binding amphiphilic prodrugs of the cytotoxic drug Ptx and of the IDO1 inhibitor NLG919 (Nlg), and the subsequent formulation of these prodrugs into HSA-binding and carrier-free nanobinders (NBs). It is noteworthy that the strategic use of prodrugs assumes particular significance, as it ensures a more precise control over drug release, imparts amphiphilicity, and enhances NBs stability within physiological contexts. The main objectives of this study are the preparation of HSA-binding prodrugs and their corresponding NBs and the assessment, in cell-free experiments and in two- and three-dimensional *in vitro* models of TNBC, that the newly synthesized prodrugs and NBs maintain the antitumor cytotoxicity and the ICD effect of Ptx while inhibiting IDO1 enzymatic activity. This exploratory study lays the foundation for further *in vitro* and *in vivo* preclinical validation of carrier-free and biomimetic NBs as a potential therapeutic option for treating TNBC and solid tumors characterized by increased IDO1 expression (Girithar et al., 2023).

## 2 Materials and methods

### 2.1 Materials–Chemistry

Pure Ptx was kindly provided by Indena SpA (Italy). NLG919 (Nlg) was purchased from Zentek Srl (Italy). Unless otherwise stated, all other reagents were purchased from Sigma-Aldrich (Merck, Italy). All reagents were used as obtained from commercial sources unless otherwise indicated; solvents for synthesis were dried over standard drying agents and freshly distilled prior to use. Ultrapure water (uH_2_O) was produced using a Sartorius Arium Pro^®^ system. Proton and carbon-13 nuclear magnetic resonance (^1^H-NMR and ^13^C-NMR) spectra were recorded on a 500 MHz Agilent DD2 PremiumCompact Plus NMR spectrometer, equipped with a single ADC console and a OneNMR probe, and a 400 MHz Varian Mercury NMR spectrometer, equipped with an AutoSwitchable probe. Deuterated solvents–deuterium oxide (D_2_O), methanol-*d*_4_ (CD_3_OD), chloroform-*d* (CDCl_3_), dimethyl sulfoxide-*d*_6_ (DMSO-*d*_6_), acetone-*d*_6_–were purchased from Eurisotop (France) and used as specified for each compound. ^1^H chemical shifts values (δ, in ppm) are referenced to the residual non-deuterated components of the NMR solvents. ESI-MS data were acquired on a TSQ Quantum Access Max Triple Quadrupole mass spectrometer. Flash chromatography was performed on a Teledyne Isco CombiFlash Rf 200 system using RediSep Rf normal‐phase silica gel flash columns (230–400 mesh). Thin-layer chromatography (TLC) was performed on plastic plates coated with silica gel 60 and fluorescent indicator F254. All compounds tested in biological assays were >95% pure, as determined by high-performance liquid chromatography analysis with ultraviolet detection (HPLC–UV) performed on a Shimadzu Nexera XR UHPLC system equipped with a LC-40D XR pump, a SIL-40C XR autosampler, a DGU-405 degassing unit, a CTO-40S column oven and an SPD-M40 photodiode array (PDA) detector. The purity of intermediates was >90%, unless otherwise stated. Solvents and reagents for analytical studies–methanol (MeOH), ethanol (EtOH), acetonitrile (ACN), trifluoroacetic acid (TFA), ammonium acetate (NH_4_OAc), acetic acid (AcOH), dimethyl sulfoxide (DMSO)–were of HPLC grade or higher.

All synthetic procedures and compounds characterization including NMR and LC-MS spectra (Supplementary Figures S2–S21) are reported in the Supplementary Material section.

### 2.2 HSA binding affinity

Surface plasmon resonance (SPR) analyses were performed using a Biacore X100 (GE Healthcare, Italy). Binding affinities, expressed as dissociation constants (K_D_) were determined using a CM5 sensor-chip previously functionalized with an anti-HSA antibody (polyclonal anti-human albumin antibody, Sigma-Aldrich Merck) which was covalently immobilized on both the flow cells by amine coupling using an Amine Coupling kit (Cytiva Italy); the functionalized sensor chip was used to immunocapture HSA (96%, essentially fatty acid free, product code A1887; Sigma-Aldrich, Merck, Italy). Analyses were performed using a single-cycle approach: at each single-cycle analysis, a 50 µM HSA solution was flowed on the surface of the active cell at a flow rate of 5 μL/min for 840 s to allow HSA immunocapture. tEB and pMAC1 solutions were prepared at concentrations of 0.781, 1.56, 3.12, 6.25 and 12.5 µM while pMAC2 and nMAC were prepared at concentrations of 1.56, 3.12, 6.25, 12.5 and 25 µM in phosphate buffered saline (PBS) pH 7.4 with 0.05% polysorbate 20% and 2% DMSO. Analyses were performed at a flow rate of 30 μL/min. Each analyte solution was allowed to interact with HSA for 120 s. Two subsequent regeneration steps were performed at the end of the analysis cycle by subsequently injecting a 100 mM glycine hydrochloride (Gly) solution and a 50 mM sodium hydroxide (NaOH) solution at a flow rate of 10 μL/min for 40 s for surface regeneration. The SPR sensorgrams were corrected by a double referencing procedure and globally fitted using a 1:1 binding model. K_D_ values were calculated using the Biacore X100 Evaluation Software version 2.0.1 in affinity mode (GE Healthcare). All SPR experiments were performed in duplicate, and binding affinities for each analyte were averaged over the two experiments.

### 2.3 Prodrug stability

The stability of prodrugs (pMal, pMAC1, pMAC2, nMAC) was determined on 10 µM samples prepared by diluting stock solutions (1 mM in DMSO) to 40 µM in EtOH before the addition of PBS pH 7.4 (final solvent: PBS/EtOH 75:25, v/v). Four different concentrations of dithiothreitol (DTT) were used to test the sensitivity of prodrugs towards the redox potential of the environment (0 mM, 0.2 mM, 1 mM, 10 mM). The effect of HSA on stability was also evaluated on equimolar mixtures (10 µM) of protein and prodrugs and tested under the same redox conditions. Two independent samples were prepared for each condition and submitted without further purification to HPLC-UV analysis, which was carried out on the Shimadzu Nexera XR system with a Phenomenex Jupiter C4 column (150 × 2.0 mm I.D., 5 µm particle size, 300 Å pore size) using an injection volume of 20 µL. Gradient elution was achieved by mixing mobile phases A (TFA 0.1% in uH_2_O, v/v) and B (TFA 0.1% in ACN, v/v) at a constant flow rate of 0.5 mL/min, according to the following time program: 0 min, 20% B; 0.50 min, 20% B; 2.75 min, 35% B; 5.75 min, 45% B; 7.25 min, 55% B; 9.30 min, 55% B; 9.50 min, 20% B, 13.33 min, 20% B. Column oven and autosampler temperatures were set to 40°C and 20°C, respectively. Chromatograms at the relevant detection wavelengths (556 nm for pMAC1, pMAC2 and nMAC; 392 nm for pMal) were extracted from PDA data (600–200 nm). Analytes were identified based on their retention times (*t*_R_) and PDA-extracted UV spectra (Supplementary Figure S22). The quantities of unreacted prodrugs in the presence and absence of HSA were monitored at four different time steps after sample preparation (10 min, 1 h, 2 h, 3 h) by summing the peak areas of pMal (*t*_R_ = 10.9 min), pMAC1 (*t*_R_ = 10.2 min), pMAC2 (*t*_R_ = 9.8 min) or nMAC (*t*_R_ = 6.0 min) to that of HSA-bound prodrugs (*t*_R_ = 6.7 min for HSA). Prodrug stabilities (% m/m) as a function of reaction time were finally derived for each redox condition from the cumulative peak areas of prodrugs relative to those obtained in the absence of DTT at the initial time step of the assay (Supplementary Figure S23).

### 2.4 Nanobinders preparation and characterization

For the preparation of single-component micelles preparation, @pMal and @NlgD, uH_2_O was added to a solution of prodrugs dissolved in EtOH under vigorous stirring. After stirring at room temperature (r.t.) for 2 h, the suspension was purified by ultrafiltration (UF) with a regenerated cellulose centrifugal filter (MWCO:10 kDa; Amicon Ultra, Millipore, Merck, Italy), centrifuged and resuspended in uH_2_O by vortexing at 8,000 rpm (5 min) and at 4,000 rpm (2 min) (3x). All experimental details on prodrugs stock solutions and relative ratios are reported in Supplementary Table S3.

Multi-component micelles including fluorescently labeled NBs, e.g., @pMal-NlgD, NBs and f-NBs, were prepared by dissolving the bioresponsive prodrug(s) and the Nile Red dye (for fluorescent NBs) in *N*,*N*-dimethylformamide (DMF) or EtOH and the obtained solution was slowly added to uH_2_O under vigorous stirring to form micelles. After stirring at r.t. for 2 h, the suspensions were purified as previously described for single component micelles. All experimental details on prodrugs and Nile Red stock solutions and relative ratios are reported in Supplementary Tables S4–S6.

The hydrodynamic diameter and polydispersity index (PDI) of micelles were determined by dynamic light scattering (DLS) at 25°C using a NanoBrook Omni particle size analyzer (Brookhaven Instruments Corporation, United States) equipped with a 35 mW red diode laser (nominal wavelength 640 nm). The measurements were performed on samples prepared by diluting 50 μL of micelles’ solution in 1.6 mL of uH_2_O (representative concentration = 60 mg/mL). The ζ-potential of micelles was measured at 25°C using the same instrument to evaluate the electrophoretic mobility. Stability studies on micelles suspensions (50 mg/mL) were performed over time in PBS pH 7.4 (37°C, 24 h) with or without HSA (35 mg/mL) and fetal bovine serum (FBS; 10% v/v). In a typical experiment, 0.2 mL of micelles suspension (2.5 mg/mL) were diluted with 1.8 mL of the selected stability medium, while maintaining them at 37°C. Changes in particles’ size distribution were monitored by DLS. NB morphology was analyzed by transmission electron microscopy (TEM). Sample micelles (0.1 mg/mL), pre-incubated or not with HSA (10:1, m/m), were dispensed as a drop on a carbon-coated nickel grid and after 20 min, any excess of the solution was absorbed by filter paper. The nanoformulation was subsequently observed with a Jeol Jem-1011 transmission electron microscope (Jeol Jem, Peabody, United States).

### 2.5 Drug release

The release of drugs from NB@5 was evaluated by equilibrium dialysis and HPLC-UV analysis, which was performed on the Shimadzu Nexera XR system with a Phenomenex Kinetex C18 column (150 × 4.6 mm I.D., 5 µm particle size, 100 Å pore size) using an injection volume of 50 µL. Gradient elution was achieved by mixing mobile phases A (NH_4_OAc 20 mM in uH_2_O, pH 5.1 buffer with AcOH) and B (NH_4_OAc 20 mM in MeOH) at a constant flow rate of 1.0 mL/min, according to the following time program: 0 min, 50% B; 2.50 min, 50% B; 6.50 min, 90% B; 9.00 min, 90% B; 9.10 min, 50% B; 13.33 min, 50% B. Column oven and autosampler temperatures were set to 40°C and 10°C, respectively. Chromatograms at the relevant detection wavelengths (228 nm for Ptx, 271 nm for Nlg) were extracted from PDA data (600–200 nm). NB@5 suspensions were prepared in uH_2_O at a nominal concentration of 202.4 μg/mL, equivalent to a Ptx concentration of 50 μg/mL after complete reaction of pMAC2. Samples for dialysis (250 μL) were then loaded into Pur-A-Lyzer Mini dialysis devices (MWCO: 12 kDa) and dialyzed against 2.5 mL of release medium (PBS/EtOH 75:25 v/v; total volume: 2.75 mL) for 48 h at 37°C; three different DTT concentrations (0 mM, 1 mM, 10 mM) were tested to assess the sensitivity of drug release towards the redox potential of the environment. During dialysis, aliquots (150 μL) were sampled from release media at 14 time points (one every 20 min from 5 min to 85 min, one every hour from 2 h to 7 h, then 24 h, 28 h and 48 h), replaced with the same volume of fresh medium and submitted to HPLC-UV analysis with duplicate injections. The exact contents of Ptx and Nlg in NB@5 suspensions were determined after a 1:10 dilution, achieved by sequential addition of ACN (5 volumes) and DTT (50 mM in uH_2_O; 4 volumes), yielding samples (20.24 μg/mL) dissolved in a solution of DTT 20 mM in uH_2_O/ACN 50:50 (v/v); sonication cycles (10 min) were performed after each dilution step to facilitate the full solubilization of prodrugs. The resulting samples (*n* = 3) were incubated at 45°C for 3 h to drive the drug release reaction from prodrugs to completion, then submitted to HPLC-UV analysis with duplicate injections. The released quantities of Ptx and Nlg at each time point were determined from the cumulative peak areas for the main stereoisomers of both drugs (Supplementary Figure S24)–Ptx (*t*_R_ = 7.5 min) and 7-epi-Ptx (*t*_R_ = 7.8 min), resulting from the epimerization of Ptx at pH 7.4; (*R*,*S*)/(*S*,*R*)-Nlg (*t*_R_ = 7.9 min) and (*S*,*S*)/(*R*,*R*)-Nlg (*t*_R_ = 8.1 min)–using calibration curves obtained by linear regression (Supplementary Table S1 and Supplementary Figure S25), then expressed as mass fractions (% m/m) relative to their exact contents in NB@5 suspensions (Supplementary Table S2). Kinetic profiles were derived by plotting mass fractions as a function of dialysis time.

### 2.6 Cell lines

Human TNBC cell lines MDA-MB-468 and MDA-MB-231 were purchased from American Type Culture Collection (ATCC, United States). MDA-MB-468 and MDA-MB-231 cells were grown in Dulbecco’s Modified Eagle Medium-High Glucose (DMEM-HG) with Glutamax^TM^ supplemented with 10% heat-inactivated FBS and antibiotics (100 U/mL streptomycin, 100 μg/mL penicillin G).

Human Mesenchymal stroma cell line (MSCs) was isolated, as previously described, (Martella et al., 2023), from bone marrow sample obtained from patient undergoing surgery at the Rizzoli Orthopedic Institute (Bologna, Italy) after informed consent according to the protocol approved by the local ethical committee (n 0029817/2015). MSCs were expanded in complete medium consisting of α-MEM supplemented with 20% of FBS and 1%GlutaMax, changing medium twice for week and detached, using triple select, at 60%–70% confluency. Cell culture media and supplements were purchased from Life Technologies (Italy), while sterile plasticware was purchased from Falcon (Corning, United States).

### 2.7 *In vitro* studies on TNBC cells monolayers

#### 2.7.1 Cytotoxicity of prodrugs and nanobinders

The cytotoxic effects of synthetized Ptx prodrugs, NBs and controls (e.g., reference drugs) in TNBC cells and MSCs were assessed using CellTiter 96^®^ AQueous One Solution Cell Proliferation Assay (MTS, Promega, Italy). MDA-MB-468 (8×10^3^ cells/well), MDA-MB-231 (8×10^3^ cells/well) and MSCs (4 × 10^3^ cells/well) were seeded in 96-well plates and allowed to adhere for 24 h. Cells were treated for 24 or 48 h with increasing concentrations of Ptx-based prodrugs or NBs diluted in culture media. For the MTS assay, the medium was replaced with 100 μL of serum-free medium and 20 μL of the CellTiter 96^®^ reagent. After 60 min, 90 min (TNBC cells) or 2 h (MSCs), the absorbance at 492 nm was measured with a Multiskan GO (Thermo Fischer Scientific, United States) plate reader and cell viability was expressed as a function of absorbance relative to that of control cells (considered as 100% viability). Cell viability data were elaborated with the software GraphPad Prism 9.5 to obtain dose-effect curves and calculate the half maximal inhibitory concentration value (IC_50_) of each tested compound. To assess the effectiveness of NBs in a simulated TME (*i.e.,* different reducing conditions), the viability assay was performed in the presence of DTT (10 μM and 10 mM) and HSA (NB@5/HSA 1:10 m/m). Briefly, NB@5 were incubated with HSA for 30 min at 37°C and for further 2 h in the presence of DTT. Cells were then treated for 48 h with pre-treated NB@5 at the corresponding concentrations of Ptx of 0.01 µM and 0.1 µM.

#### 2.7.2 Kynurenine *in vitro* assay: IDO1 inhibition

The IDO1 inhibitory activity of Nlg, Nlg prodrugs and Nlg-containing NBs was assessed by measuring the kynurenine content in cell culture media. MDA-MB-468 cells (8×10^3^ cells/well) were seeded into 96-well plate and allowed to grow overnight. Cells were stimulated with 50 ng/mL of recombinant human interferon-γ (IFN-γ; Life Technologies, Italy) to induce the expression of IDO1 and simultaneously treated with three concentrations of Nlg-containing formulations (e.g., 0.1, 1 and 10 μM). After incubation (24 h), 140 μL of the supernatant from each well were transferred to a new 96-well plate and mixed with 50 μL of 30% trichloroacetic acid. The plate was incubated for 30 min at 50°C to facilitate protein precipitation and then centrifuged at 2,500 rpm. Then, 100 μL of the resulting supernatants were collected in a new plate and mixed with an equal volume of Ehrlich reagent (2% p-dimethylaminobenzaldehyde in glacial AcOH, w/v). After 10 min of incubation, the absorbance at 490 nm was determined using the Multiskan GO microplate reader.

#### 2.7.3 Immunogenic cell death (ICD)

The induction of immunogenic cell death by Ptx-containing formulations on the MDA-MB-468 and MDA-MB-231 cell lines was assessed by analyzing CRT exposure on the plasma membrane, migration of HMGB1 from the nucleus to the cytoplasm and ATP release. CRT exposure and HMGB1 migration were detected by immunofluorescence. Briefly, 5×10^4^ cells were seeded on 4-well chamber slides (Falcon, United States) and grown overnight at 37°C in a humidified environment with 5% CO_2_. Cells were treated with 0.1 and 1 μM of Ptx-containing formulations and further incubated for 12 h. Cells were then washed with ice-cold PBS twice, fixed with 4% paraformaldehyde for 15 min at r.t. and permeabilized with 0.1% Triton X-100 for 10 min at r.t. (this last step was done only for HMGB1 detection). Cells were then blocked using a solution containing 2% HSA and 20 mg/mL Gly for 1 h at 37°C, prior to immunolabeling with rabbit anti-CRT-Alexafluor 488 (1:500) or anti-HMGB1-Alexafluor 488 (1:250) antibodies overnight at 4°C. Both antibodies were purchased from Abcam. After three washes with PBS, cell nuclei were stained with Hoechst 33342. The slides were then further washed three times with PBS and sealed with VectaShield Plus mounting medium (VectorLabs, United States). Cells were imaged with a ZEISS LSM 900 with Airyscan 2 confocal microscope and, subsequently, analyzed with the software Fiji. (Schindelin et al., 2012). Briefly, the region of interest (ROI) was defined for each cell and the integrated density, corresponding to fluorescence, was measured. ATP release from cells was measured using the RealTime-Glo™ Extracellular ATP Assay (Promega, Italy) following the manufacturer’s specifications. Briefly, 1×10^4^ cells were seeded in white 96-well plates with clear bottom and grown for 24 h. The day after, cells were treated with increasing concentrations of Ptx-containing formulations diluted in complete medium supplemented with RealTime-Glo Extracellular ATP Assay Reagent. Luciferin luminescence was then monitored using Victor 3 multimodal microplate reader (Perkin Elmer, United States).

#### 2.7.4 Intracellular uptake of nanobinders

The hydrophobic fluorophore Nile Red (NR) was loaded in selected NB formulations to assess their internalization by flow cytometry. 8×10^4^ MDA-MB-468 cells were seeded in 24-wells plate in complete medium and allowed to adhere for 24 h. Cells were then treated for 2 h with NBs (1 μM NR concentration) diluted in culture medium in the absence or in the presence of 10% FBS. At the end of the incubation time, cells were washed twice with Versene solution and detached from the plates with trypsin, which was then neutralized by the addition of FBS. Cells were centrifuged and resuspended in Versene before measuring NR fluorescence using a BD Fortessa^TM^ X-20 flow cytometer (Becton Dickinson, United States). A blue laser (488 nm) was used to excite the fluorophore and its fluorescence was detected in the PE-Texas Red channel (610/20 filter). For each sample, 10^4^ events were acquired and analyzed using the BD FACSDiva 9.0 software.

### 2.8 *In vitro* studies on TNBC spheroids

#### 2.8.1 Spheroids generation

MDA-MB-468 spheroids were generated into 96-wells ultra-low attachment plates (Nunclon^TM^ Sphera^TM^ U-shaped bottom microplates, Thermo Fisher Scientific, United States) using the liquid overlay-technique. Briefly, 3.5×10^3^ cells/well were seeded in DMEM supplemented with 10% FBS and 6 μL/mL of collagen I (BD Biosciences, United States). Immediately after seeding, cells were centrifuged for 3 min at 100×*g* to promote cell aggregation and allowed to grow in the incubator for 4 days at 37°C under 5% CO_2_ before their use in the following experiments.

#### 2.8.2 Cytotoxicity in spheroids

Cell viability reduction in treated spheroids was assessed using the CellTiter-Glo^®^ 3D Cell Viability Assay (Promega Italia), based on the measurement of intracellular ATP content. Four-days-old MDA-MB-468 spheroids were incubated with fresh medium containing 10% FBS and increasing concentration of prodrugs or NBs for 72 h before measuring viability. For the assay, 100 μL of medium were left in each spheroid well and 100 μL of CellTiter-Glo^®^ 3D reagent were added; the well was incubated at r.t. for 30 min before transferring the 200 μL of sample/wells in a blank 96/well (Perkin Elmer, Waltham, MA, United States). Luminescence was measured with a Perkin Elmer VICTOR^TM^X3 instrument. Cell viability was expressed as a function of luminescence relative to that of control cells (considered as 100% viability).

#### 2.8.3 Kynurenine *in vitro* assay: IDO1 inhibition

The inhibitory activity on IDO1 of Nlg, Nlg prodrugs and Nlg-containing NBs was assessed by measuring the kynurenine content in cell culture media. 3.5×10^3^ MDA-MB-468 cells/well were seeded in 96-well Nunclon Sphera 3D plates (Thermo Fisher, Italy) in complete cell culture medium supplemented with collagen I and allowed to grow for 96 h. Spheroids were stimulated with 50 ng/mL of recombinant human interferon-γ (IFN-γ; Life Technologies, Italy) to induce the expression of IDO1 and simultaneously treated with three concentrations of Nlg formulations (e.g., 0.1, 1 and 10 μM). After incubation (48 h), 140 μL of the supernatant from each well were transferred to a new 96-well plate and mixed with 50 μL of 30% trichloroacetic acid. The plate was incubated for 30 min at 50°C to facilitate protein precipitation and then centrifuged at 2,500 rpm. Then, 100 μL of the resulting supernatants were collected in a new plate and mixed with an equal volume of Ehrlich reagent (2% p-dimethylaminobenzaldehyde in glacial AcOH, w/v). After 10 min of incubation, the absorbance at 490 nm was determined using Multiskan GO microplate reader.

### 2.9 Statistical analysis of biological data

Biological data shown in this study are expressed as mean ± SD. All experiments were performed at least twice and carried out in triplicate, unless otherwise stated in the respective legend figure. All statistical analyses were performed with GraphPad Prism 9.5 (GraphPad; San Diego, CA, United States) using one-way ANOVA test and Tukey’s multiple comparison as a post-test in Figure 8C and for CRT and HMGB1 quantification. Data reported in Figure 4, Figure 6 and Figure 8A were analyzed using the 2-way ANOVA test and Bonferroni’s multiple comparison as a post-test. Results were statistically significant for *p*-values < 0.05 (*p-values< 0.05, ** *p*-values< 0.01, *** *p*-values< 0.001 and ****p-values< 0.0001).

## 3 Results and discussion

### 3.1 Synthesis of bioresponsive and HSA-binding building blocks

**FIGURE 1:**
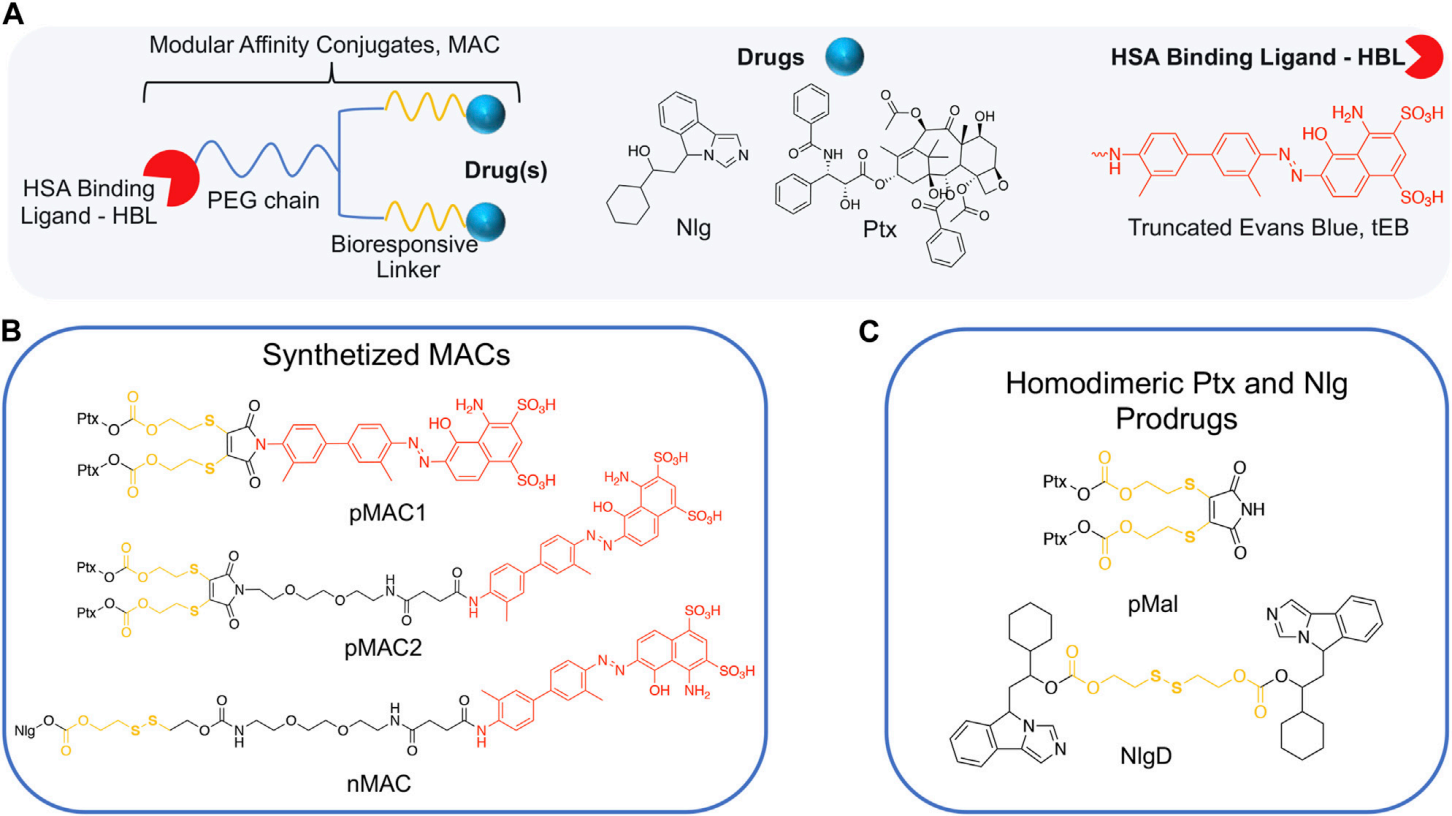
**(A)** General structure of Modular Affinity Conjugates (MAC). **(B)** Chemical structures of synthesized MACs derivatives, e.g., pMAC1, pMAC2 and nMAC. **(C)** Chemical structures of homodimeric Ptx and Nlg prodrugs, e.g., pMal and NlgD.

We conceived the design and preparation of bioresponsive Modular Affinity Conjugates (MAC, Figure 1) with diverse functionalities. These MACs consist of an HSA-binding ligand (HBL) capable of selectively binding to the protein *in vivo*, along with one or two drug molecules, such as paclitaxel (Ptx) or NLG919 (Nlg), connected *via* a bioresponsive linker. As the HBL, we selected the truncated Evans Blue dye (tEB, Figure 1; Supplementary Scheme S1). tEB maintains the HSA-binding properties of the native EB, and allows for conjugation with other chemical entities thanks to the presence of a free NH_2_ terminal group (Chen et al., 2016). Instead of exploiting the covalent bonding with Cys34 on HSA, we intentionally focused the design of our systems on the non-covalent tEB ligand to avoid unwanted cross-reactions with Cys and Lys residues on other blood and cell proteins, as well as to overcome potential issues posed by lower availability of free Cys34 associated with the elevated oxidative stress present in cancer patients (Maciążek-Jurczyk et al., 2021).

**Scheme 1:**
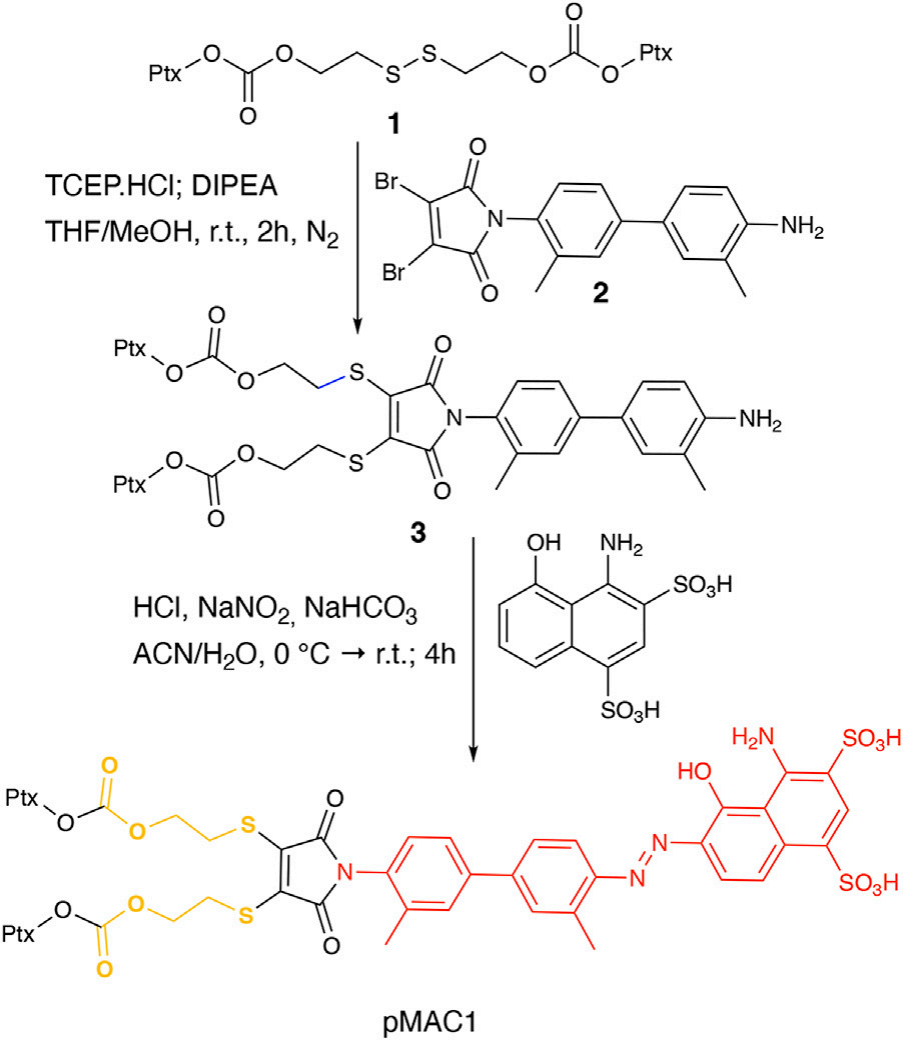
Synthetic route to pMAC1.

**Scheme 2:**
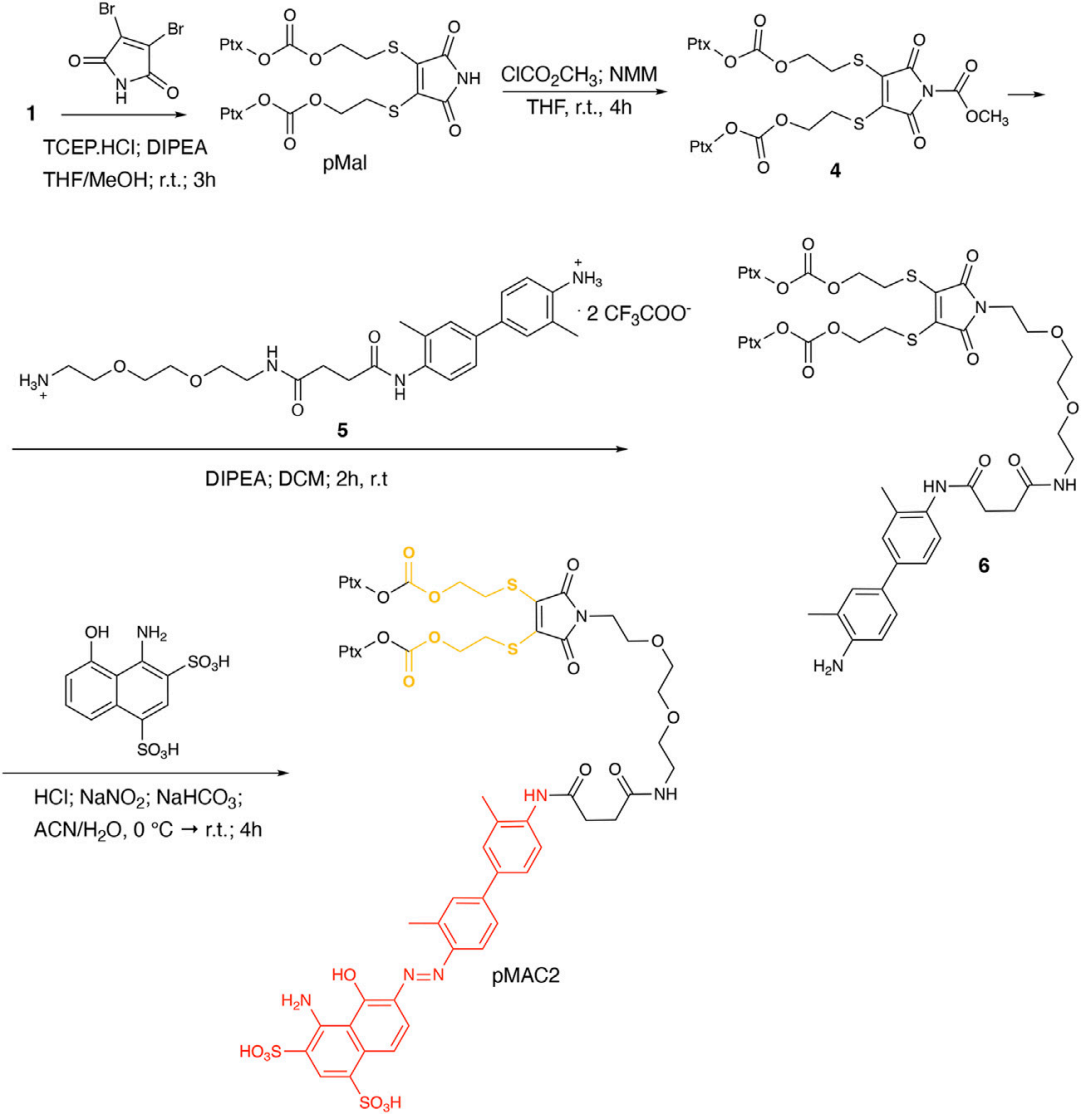
Synthetic route to pMAC2.

This work started with the synthesis of the bioresponsive prodrugs required for the fabrication of the corresponding carrier-free nanosystems. Upon exploring different synthetic strategies and considering the challenges of semi-synthetic modifications of natural compounds, such as Ptx, we were able to setup a complex but straightforward process allowing to isolate in good yields pMAC1 (Scheme 1) and pMAC2 (Scheme 2) derivatives in good yields. The Ptx dimer **1** was synthesized in 85% yield starting from commercially available Ptx according to an optimized synthetic procedure (Supplementary Scheme S2). Upon reaction with compound **2** and in the presence of tris(2-carboxyethyl)phosphine hydrochloride (TCEP^.^HCl) and N,N-diisopropylethylamine (DIPEA), the corresponding derivative **3** was obtained in 65% yield. The final diazotization reaction afforded the desired compound pMAC1 in 70% yield upon dialysis purification (Scheme 1).

To explore the effect of the linker between the prodrug moiety and the tEB framework on the formation of bioresponsive NBs, a second derivative, namely pMAC2, was prepared starting from the Ptx dimer **1**, which was reacted with commercially available 2,3-dibromomaleimide to afford compound pMal in >70% yield (Scheme 2). Following a literature procedure applied to different compounds (Castañeda et al., 2013), derivative **4** was isolated in quantitative yield and coupled with compound **5** (Scheme 2; Supplementary Scheme S3) to afford **6** in 50% yield. The final diazotization step allowed to isolate the target compound pMAC2 in quantitative yield upon dialysis purification.

Conjugates pMAC1 and pMAC2 are characterized by the presence of 2 molecules of Ptx connected through a bioresponsive linker to the HSA-targeting moiety tEB and are reported here for the first time. It is worth noticing that, unlike what previously reported on other drug-tEB conjugates in which tEB is coupled with the target molecule as the final reaction step (Chen et al., 2017; Wang et al., 2018), the procedure herein developed foresees the diazotization reaction as the last synthetic step. Indeed, in our hand, the direct coupling between tEB and any precursor’s prodrug afforded poor yields and required tedious and unsuccessful purification procedures. Conversely, the mild conditions of the diazotization reaction allow to easily access the desired compounds. In principle, this unprecedented synthetic procedure can drive the simple and straightforward tEB functionalization of several active ingredients with the aim of improving their blood circulation time and preferential tumor accumulation by binding to endogenous HSA.

**Scheme 3:**
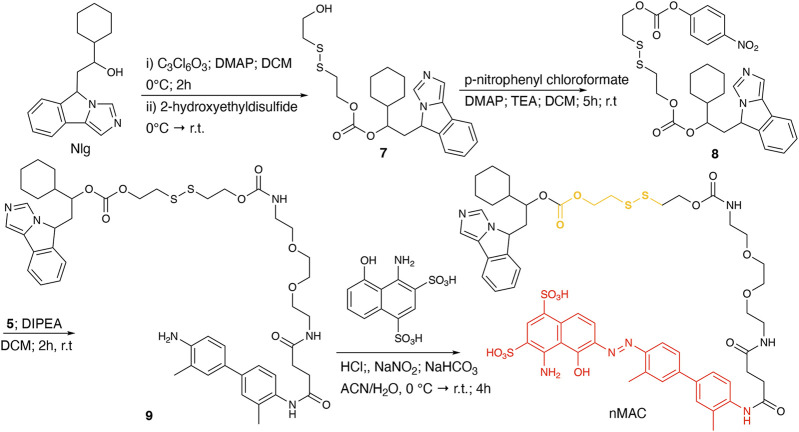
Synthetic route to nMAC.

For the preparation of nanobinders (NBs), several parameters must be thoughtfully considered, including their stability over time, the percentage of tEB functionalization to allow proper binding with HSA, and the Nlg/Ptx ratio, which should be in favor of Nlg (Sonpavde et al., 2020). Moreover, it is established that homodimeric disulfide prodrugs, such as Nlg dimer, i.e., NlgD (Figure 1; Supplementary Material), (Feng et al., 2018) can enhance the stability of prodrug-based nanoassemblies and facilitate the reduction-responsive release of active drugs (Zhou et al., 2021; Liu et al., 2022; Zhong et al., 2023). Therefore, to achieve greater flexibility in the composition of NBs concerning stability and drug ratio, we chose to synthesize a monomeric HSA-binding analogue of Nlg, i.e., nMAC (Scheme 3), to be combined with pMACs and NlgD (see § 3.4).

nMAC was prepared starting from commercially available Nlg that was reacted with triphosgene and 2-hydroxyethyldisulfide affording compound **7** in 52% yield (Scheme 3). Activation of the free hydroxyl group on the linker chain with *p*-nitrophenyl chloroformate afforded derivative **8** in 60% yield that upon reaction with compound **5** (Supplementary Scheme S3) provided the precursor **9** in 32% yield. The diazotization step followed by dialysis purification allowed to isolate the nMAC conjugate in 70% yield (Scheme 3).

### 3.2 MACs characterization: HSA binding affinity of Ptx-based MACs

A SPR analysis was performed to confirm that the chemical modifications on tEB (Supplementary Scheme S1) did not compromise the ability to bind to HSA. Within the same investigation the binding affinity of pMAC1, pMAC2 and nMAC to HSA with reference to tEB was assessed. To this aim, a HSA-functionalized sensor chip was prepared by immunocapturing HSA onto a CM5 sensor chip previously functionalized with an anti-HSA antibody. Non-specific interactions of analytes with the surface antibody were zeroed by functionalizing in parallel the reference flow cell with the same antibody. Due to the expected low dissociation rates of Evan blue-HSA complexes, a single-cycle kinetics was used for the determination of the affinity constants (Supplementary Figure S1). Interaction between tEB and HSA resulted in a *K*_D_ value of 20.7 ± 1.5 µM. Investigations on MAC derivatives confirmed that structural modifications did not hamper their ability to bind to HSA. Conversely, all derivatives showed a slightly higher affinity for HSA, when compared to tEB, although in the same order of magnitude. This behavior suggests that the higher structural complexity of MACs further contributes to complex stabilization. *K*_D_ values for tEB and MACs are summarized in Table 1.

**TABLE 1:** HSA binding affinities of tEB and Ptx-based MACs, as determined by SPR analysis. Results are averaged over two independent experiments.

Compound	*K*_D_ (µM)
tEB	20.7 ± 1.5
pMAC1	14.6 ± 0.1
pMAC2	8.1 ± 1.1
nMAC	8.5 ± 0.1

### 3.3 MACs characterization: stability of MACs in solution

**FIGURE 2:**
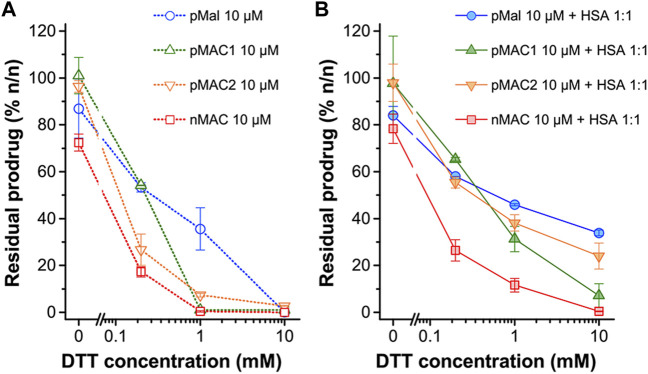
Stability of pMal and MACs (10 μM in PBS/EtOH 75:25 v/v) as a function of redox potential (DTT concentration), as determined by HPLC-UV analysis after 3 h incubation at 20°C. **(A)** In the absence of HSA and **(B)** in the presence of HSA in equimolar ratio. Results are averaged over two independent experiments.

The sensitivity of MACs towards the redox potential of the surrounding environment was evaluated by means of stability studies in solution. MACs (pMAC1, pMAC2, nMAC, Figure 1) and the main precursor of Ptx-based MACs (pMal, Figure 1) were exposed to increasing concentrations (0 to 1000-fold molar excess) of dithiothreitol (DTT), incubated for 3 h in the presence or absence of equimolar quantities of HSA and analyzed by HPLC-UV (Figure 2). A protective effect of HSA against reduction by DTT was observed for all prodrugs, albeit to different extents (Figure 2A). In particular, the stability of pMal and pMAC2 in strongly reducing conditions improved significantly in the presence of HSA, while pMAC1 and nMAC were less protected (Figure 2B). A possible explanation for this behavior in solution is given by the combined affinity of Ptx and tEB towards HSA. For pMal, the interaction with HSA is mainly driven by Ptx leading to a reduced exposure of the redox-sensitive dithiomaleimide moiety (lower release). On the other hand, MACs can interact with HSA also through the tEB moiety: however, the longer linker in the nMAC backbone leaves the disulfide bond too exposed to the environment, while the direct link between the dithiomaleimide ring and the tEB moiety may lead to a sub-optimal interaction of pMAC1 with HSA. In this framework, the PEG linker of pMAC2 seems to provide a balanced combination between HSA binding and redox sensitivity.

### 3.4 Preparation and characterization of NBs

**FIGURE 3:**
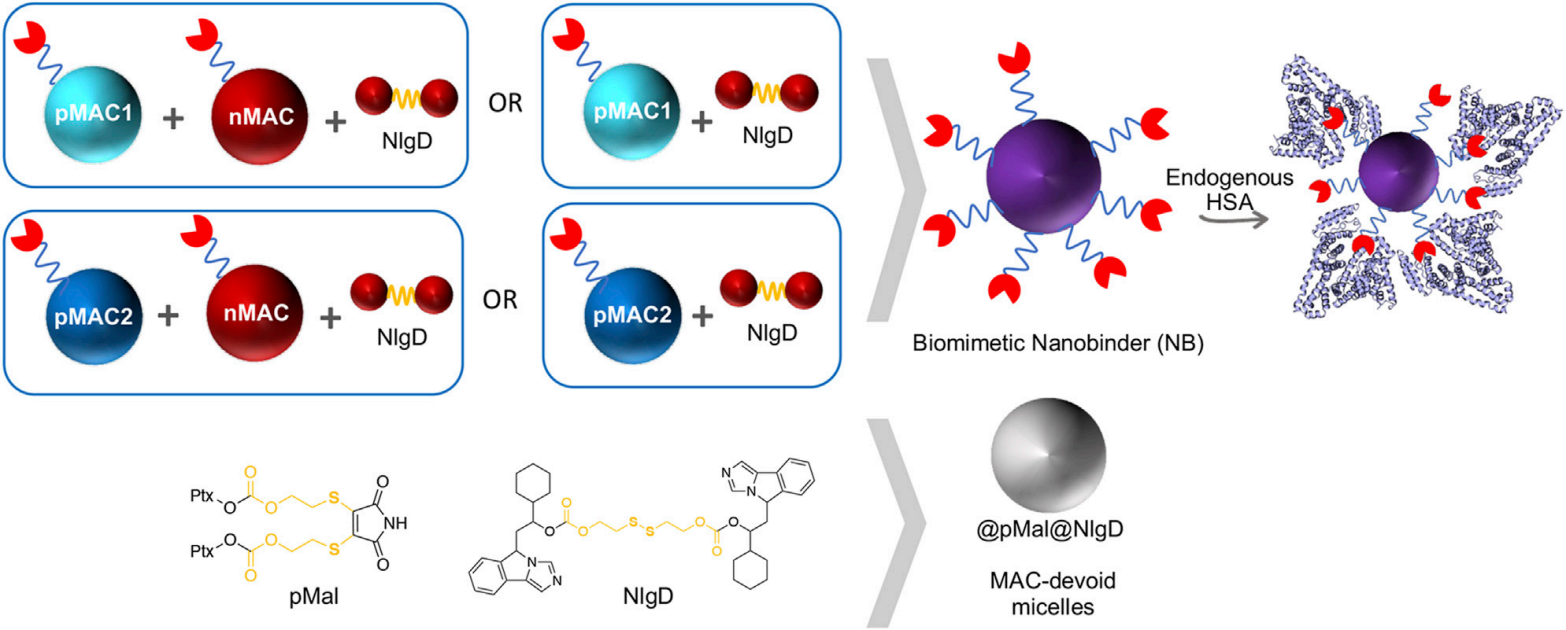
Schematic representation of biomimetic NB and MAC-devoid micelles composition.

We designed and generated different NBs including different combinations of MACs and NlgD, as well as single-component and dual-loaded “MAC-devoid” micelles, e.g., @pMal, @NlgD and @pMal-NlgD, respectively (Figure 3). As previously mentioned, the NlgD was loaded into NBs to exploit its intrinsic capability to aid the stabilization of micelles (Zhou et al., 2021) and to widen the versatility of nanosystems in terms of drug ratio, allowing to finely tune and optimize the concentration of both drugs inside the formulation and ultimately at the tumor site. HSA-binding NBs as well as MAC-devoid micelles were prepared by the nanoprecipitation method (Salvage et al., 2015), and several procedures and experimental conditions were tested to optimize their formation in terms of hydrodynamic diameter, PDI and stability in different media. The different physicochemical properties of prodrugs required tailored conditions for NBs preparation; therefore, four parameters were systematically investigated: the type of organic solvent for prodrug dissolution (DMF, DMSO or EtOH); the amount of water used for the nanoprecipitation; the order of addition of water and organic phases; the purification method (dialysis, UF or centrifugation). Ultimately, two different preparation procedures were optimized for single-component micelles (@pMal and @NlgD), and multi components micelles (@pMal-NlgD, NBs and fluorescent NBs), respectively (see § 2.4).

The preparation of MAC-devoid micelles resulted in the formation of stable particles with satisfactory size (120–150 nm) and PDI (Supplementary Table S3) (Gonda et al., 2019), as observed by DLS measurements. Regardless of the preparation procedure and drug contents, NBs-based on pMAC1 were significantly less stable than those containing pMAC2, resulting either in precipitation over time or in highly polydisperse micelles (Supplementary Table S4). Indeed, because of this high instability, it was not feasible to produce NBs based on pMAC1 with the same Nlg/Ptx ratios achieved for pMAC2-based NBs. These results, combined with those on prodrugs’ stability (Figure 2) and HSA binding affinity (Table 1), suggest that the short PEG chain in the pMAC2 structure might aid NB stabilization and interaction with the protein, prompting us to identify pMAC2 as the most promising prodrug for further NB development.

**FIGURE 4:**
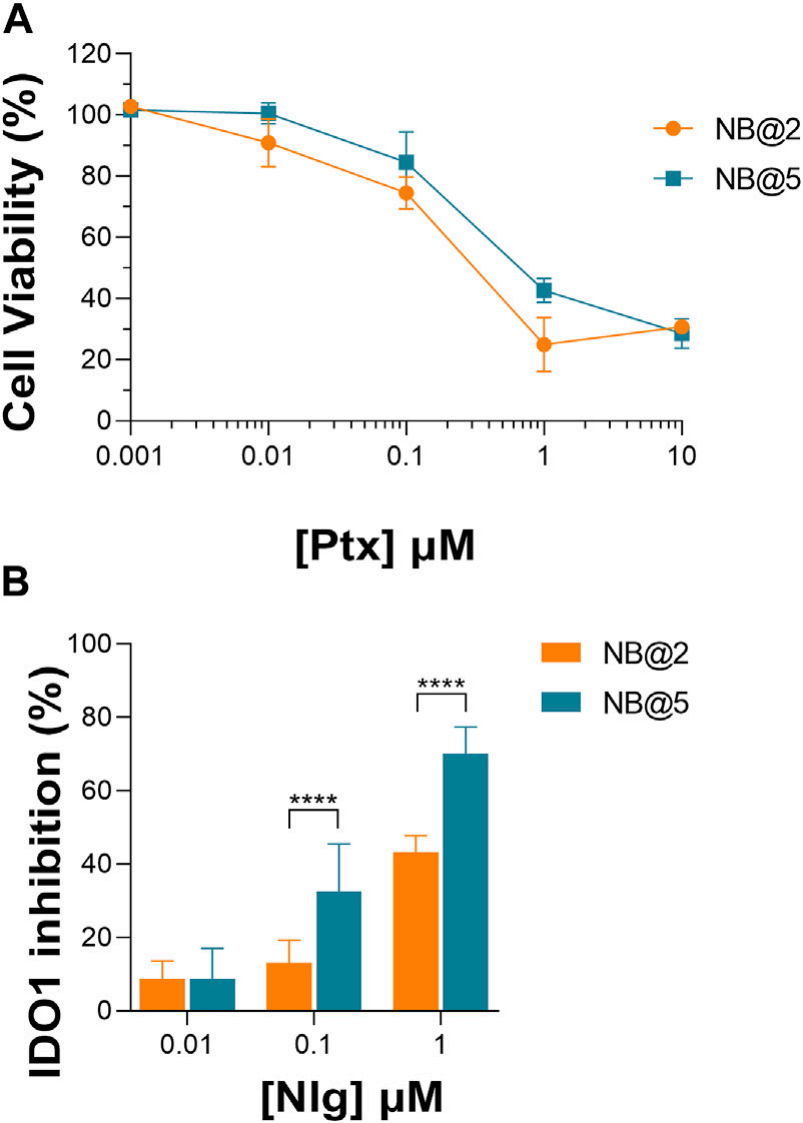
**(A)** Evaluation of cytotoxicity of NB@2 and NB@5 on MDA-MB-468 cells after 48 h of exposure. **(B)** Evaluation of IDO1 inhibition rate (%) in MDA-MB-468 cells after 24 h of exposure to NBs. Data are expressed as mean ± SD of at least two independent experiments carried out in triplicate. Data were analyzed using the 2-way ANOVA test, and Bonferroni’s multiple comparison as a post-test. Results were statistically significant for *p*-values < 0.05 (****p-values< 0.0001).

An optimization study was then carried out to identify the most favorable conditions to produce pMAC2-based NBs with different prodrug ratios (Supplementary Table S5), resulting in different Nlg/Ptx ratios (0.6–4.8, n/n), MAC loading ratios (a proxy for tEB functionalization; 39%–64%, n/n) and hydrodynamic diameters (49–242 nm). As a result, two formulations, namely NB@2 and NB@5 (Table 2), were selected as the most promising representatives of two- and three-components nanoassemblies based on their tEB functionalization, polydispersity, and reproducibility. NB@2 and NB@5 have comparable hydrodynamic diameters and MAC loading ratios, while the Nlg/Ptx ratio is more than double for NB@5 compared to NB@2. Indeed, preliminary *in vitro* cytotoxicity and IDO1 inhibition experiments on MDA-MB-268 TNBC cells showed that, despite having similar cells growth inhibition activity (Figure 4A), NB@5 were significantly more effective in reducing the IDO1 enzymatic activity (Figure 4B); therefore, we focused only on NB@5 for a deeper physicochemical and *in vitro* characterization.

**TABLE 2:** Characterization of selected pMAC2-based NBs.

Entry	Sample	Nlg/Ptx ratio (n/n)	MAC loading (n/n) (%)	Diameter (nm)	PDI
1	pMAC2@NB1	0.63	61	241.8 ± 9.6	0.278 ± 0.089
2	pMAC2@NB2 (NB@2)	1.54	39	87.2 ± 2.2	0.142 ± 0.002
3	pMAC2@NB3	4.13	37	75.0 ± 4.4	0.184 ± 0.047
4	pMAC2@NB4	4.76	26	49.3 ± 2.7	0.365 ± 0.028
5	pMAC2@NB5 (NB@5)	3.51	50	92.1 ± 1.3	0.133 ± 0.001

**FIGURE 5:**
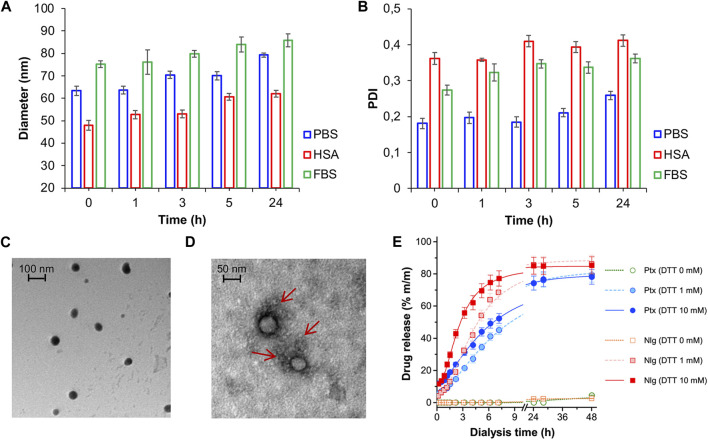
NB@5 stability in PBS, PBS + HSA and PBS + FBS at 37°C, evaluated by DLS analysis of hydrodynamic diameter **(A)** and PDI **(B)**. **(C)** TEM analysis of NB@5 (scale bar: 100 nm). **(D)** TEM analysis of NB@5 pre-incubated with HSA (10:1, m/m; scale bar: 50 nm). **(E)** Release profiles of Ptx and Nlg from NB@5 in different redox conditions, as determined by equilibrium dialysis and HPLC–UV analysis.

NB@5 are tri-components micelles composed of pMAC2, nMAC and NlgD to a final Nlg/Ptx molar ratio of 3.5 and a 50% MAC loading. DLS stability studies were performed in PBS pH 7.4 (37°C, 24 h) with or without HSA (35 mg/mL) and FBS (10%, v/v): NB@5 showed good stability profiles both in terms of hydrodynamic diameters (Figure 5A) and polydispersity (Figure 5B) in different media. Transmission electron microscopy (TEM) studies allowed to assess the morphology of NB@5. In the absence of HSA, NB@5 show a spherical shape with average dry diameters of about 50 nm (Figure 5C). Upon incubation with excess HSA (10:1, m/m), an irregular protein corona was observed around NB@5, supporting the hypothesis of an interaction between NBs and the protein mediated by tEB (Figure 5D). Indeed, treatment with HSA in the same conditions did not lead to the formation of a protein corona around MAC-devoid micelles @pMal-NlgD (Supplementary Figure S26), further confirming the key role played by tEB in promoting the binding of HSA to NBs.

The kinetics of Ptx and Nlg release from NB@5 was evaluated by HPLC–UV analysis (Figure 5E) after equilibrium dialysis (37°C, 48 h) in PBS/EtOH 75:25 v/v with increasing concentrations of DTT (0–10 mM). As expected, the release of drugs in non-reducing conditions was negligible, while the kinetic curves in reducing conditions showed that both Ptx and Nlg were released faster as the concentration of DTT increased, further supporting the redox-sensitive mechanism of prodrug conversion.

### 3.5 *In vitro* activity of bioresponsive and HSA-binding prodrugs

**FIGURE 6:**
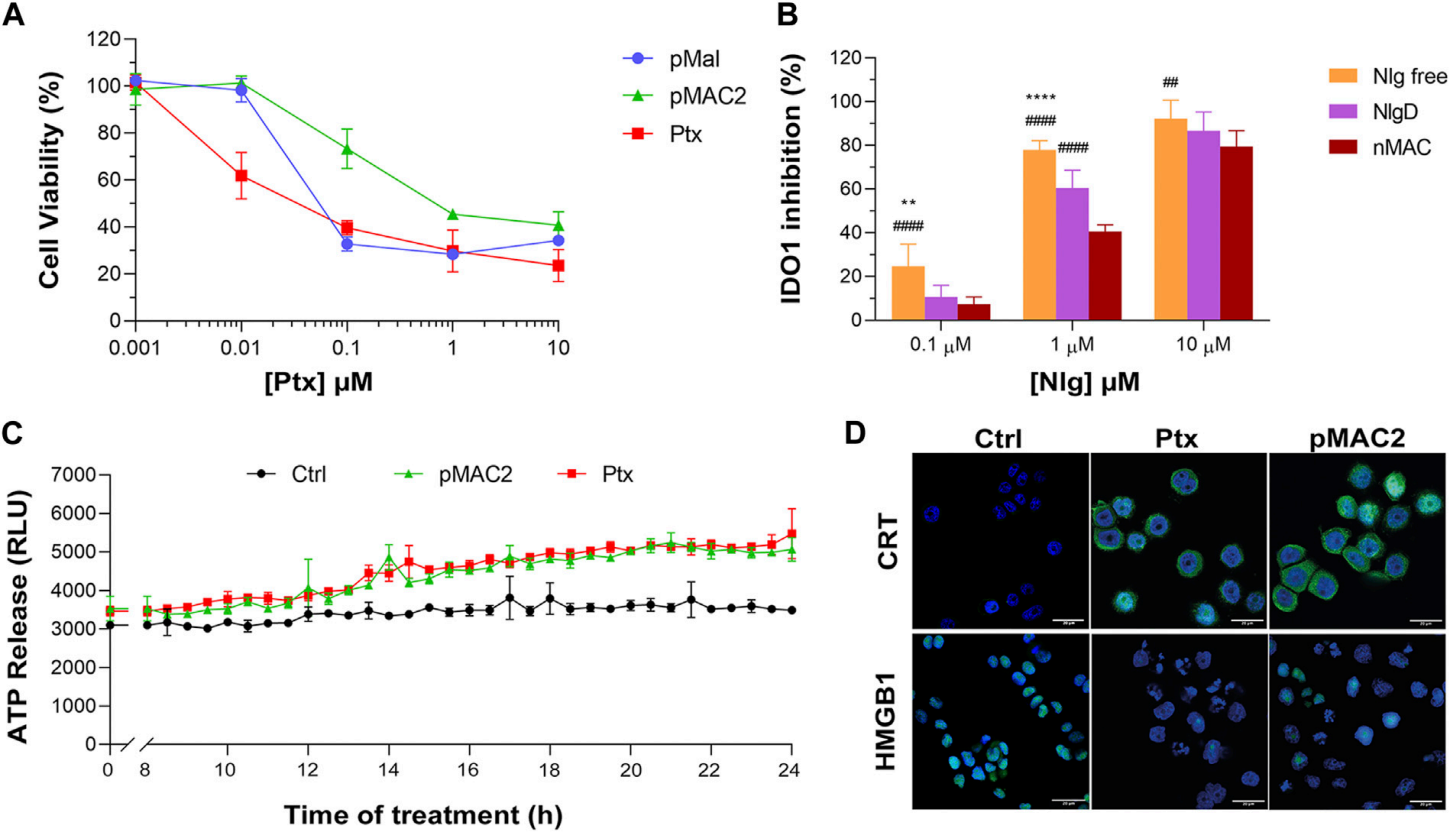
**(A)** Viability curves of MDA-MB-468 cells exposed to increasing concentrations of Ptx and Ptx bioresponsive conjugates measured by MTS tests after 48 h of incubation. **(B)** IDO1 inhibition rate (%) in MDA-MB-468 cells exposed for 24 h to different concentrations of Nlg and Nlg prodrugs. **(C)** ATP release in the cell culture medium of cells exposed to drugs at Ptx concentration 1 µM for 24 h. **(D)** Immunofluorescence images of cells incubated with the compounds at Ptx concentration 1 µM for 12 h and stained with antibody against CRT and HMGB1- FITC conjugated (green) and with Hoechst 33342 (blue) for nuclei recognition. Scale bar: 20 µm. Data are expressed as mean ± SD of at least 2 independent experiments carried out in triplicate and statistical analysis has been generated using the 2-way ANOVA test, and Bonferroni’s multiple comparison as a post-test. Results were considered statistically significant at *p*-values < 0.05 (* *p*-values < 0.05, ** *p*-values < 0.01, *** *p*-values < 0.001 and **** *p*-values < 0.0001). Data compared to NlgD are reported as *, while data compared to nMAC are indicated with #.

The capability of bioresponsive Ptx and Nlg prodrugs to induce cytotoxicity and IDO1 inhibition, respectively was assessed *in vitro* in MDA-MB-468 TNBC cells. Upon incubation with increasing concentration of pMal, pMAC2 or Ptx for 48 h, cell viability was assessed by the MTS assay at the end of incubation time. Cell viability reduction was concentration-dependent for all Ptx derivatives up to 1 μM, while above this concentration a plateau was reached (35%–20% cell viability) (Figure 6A). As expected, the IC_50_ value of pMAC2 (0.690 ± 0.005 μM) was significantly higher as those of pMal (0.050 ± 0.012 μM) and Ptx (0.034 ± 0.001 μM). The reduced cytotoxic effect of pMAC2 compared to pMal highlights the crucial role of protein binding, mediated by the tEB moiety, in controlling Ptx release. A similar trend was observed when treating MDA-MB-231 TNBC cell lines with bioresponsive prodrugs (Supplementary Figure S27A).

The IDO1 inhibitory capacity of Nlg and Nlg prodrugs (NlgD and nMAC), in MDA-MB-468 cells previously stimulated with IFN-γ to increase the enzyme expression, was measured indirectly by monitoring the decrease of Kyn release in the medium after 24 h of cell incubation. The percentage of IDO1 inhibition was almost complete when 10 μM Nlg and Nlg prodrugs were administered to cells (Figure 6B) and persisted almost unaltered for the following 24 h (Supplementary Figure S27B). At 1 μM Nlg concentration, the inhibitory activity was significantly higher for free Nlg (ca. 80%), followed by NlgD (ca. 60%) and nMAC (ca. 40%) after 24 h, while being comparable at 10 μM Nlg equivalent. As anticipated, free Nlg exhibited a higher potency in inhibiting IDO1 compared to NlgD and nMAC. This confirms our hypothesis that the disulfide bridge of the prodrugs must be cleaved to release the active drug and induce its effects. Additionally, nMAC appeared to be the least effective among the series, likely due to interactions with serum proteins, which further delayed its release. This characteristic could be especially significant *in vivo*, facilitating targeted drug release and minimizing undesired effects on healthy tissues.

One important aspect of our rationale is boosting IDO1 inhibition and Ptx-induced cytostatic effect with an early stimulation of ICD orchestrated by pMAC2. Therefore, we assessed the occurrence of the three principal ICD hallmarks, i.e., ATP release, CRT exposure on extracellular plasma membrane, HMGB1 translocation/release, (Fucikova et al., 2020), in MDA-MB-468 cells exposed to Ptx and pMAC2. The kinetic of ATP release in the culture medium of cells exposed to 1.0 μM Ptx equivalent determined a significantly different ATP release when free Ptx and pMAC2 prodrug were administered to cells (Figure 6C). Immunofluorescence and confocal microscopy were used to investigate CRT exposure and HMGB1 translocation/release in cells exposed for 12 h to 1 μM of Ptx equivalent either as free molecule or pMAC2. Control cells clearly showed green signals diffused throughout the cytoplasm and in the nuclei for CRT and HMGB1, respectively (Figure 6D). Cells exposure to 1 μM Ptx equivalent induced CRT membrane translocation and HMGB1 cytoplasmatic or extracellular release in most of the analyzed cells (Figure 6D) to a very similar extent for Ptx and pMAC2, as confirmed by signal quantification (Supplementary Figure S28) Importantly, these analyses collectively confirm that our unprecedented bioresponsive conjugate pMAC2 can induce ICD in our TNBC model similarly to unmodified Ptx.

### 3.6 NB@5 *in vitro* cytotoxicity, ICD induction and IDO1 inhibition

**FIGURE 7:**
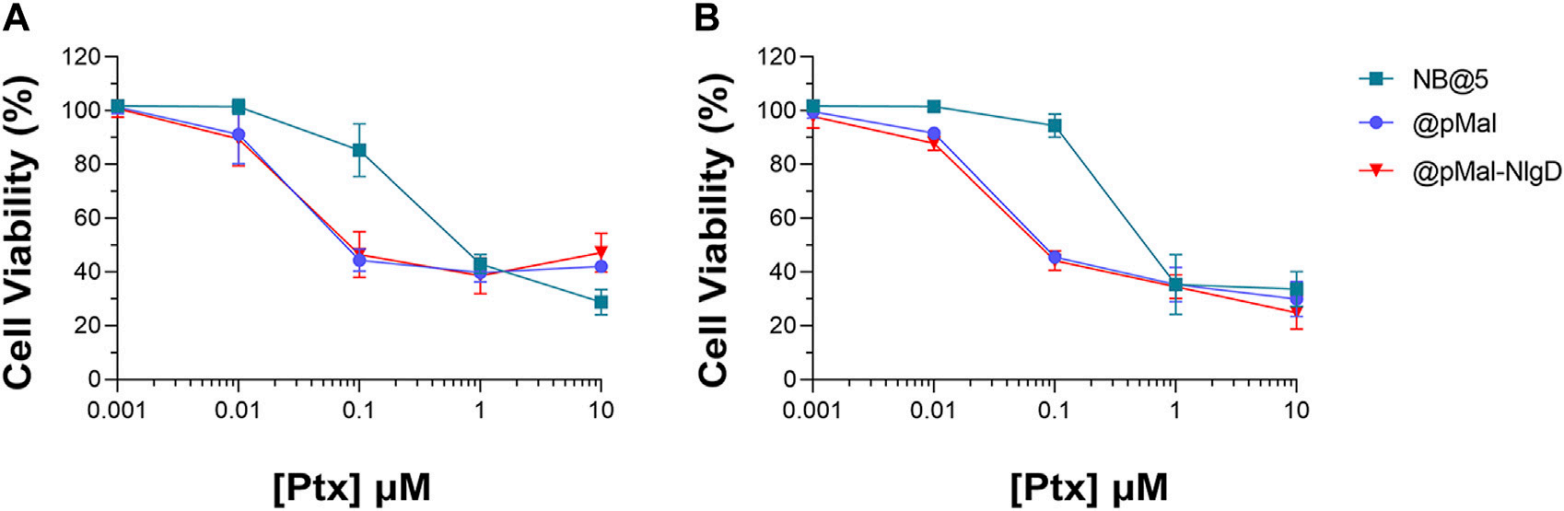
Evaluation of cytotoxicity after 48 h treatment with NB@5, @pMal and @pMal-NlgD in TNBC MDA-MB-468 **(A)** and MDA-MB-231 **(B)** cells.

The cytotoxic potential of NB@5 as compared to MAC-devoid micelles (@pMal and @pMal-NlgD) toward TNBC *in vitro* models was evaluated in MDA-MB-231 and MDA-MB-468 cancer cell lines (Figure 7). The viability curves and the corresponding IC_50_ values (Table 3) confirmed that NB@5 was the less toxic formulation against both cancer cell lines as compared to MAC-devoid micelles, which show similar viability reduction trends and IC_50_ values. Indeed, neither free Nlg nor @NlgD are cytotoxic toward TNBC cells at the tested concentrations (not shown), thus explaining the identical IC_50_ values of @pMal and @pMal-NlgD in MDA-MB-468 (∼0.02 μM) and MDA-MB-231 (∼0.04 μM) cells. Furthermore, we assessed the cytotoxic impact of NB@5 on a healthy mesenchymal stromal cell line (MSCs) isolated from patient bone marrow, revealing a reduced toxicity of NB@5 in MSCs compared to Ptx (Supplementary Figure S29).

**TABLE 3:** IC_50_ values of NB@5, @pMal and @pMal-NlgD in TNBC cell lines.

Drugs	Cell lines	
	MDA-MB-468 (µM)	MDA-MB-231 (µM)
NB@5	0.298 ± 0.036	0.233 ± 0.065
@pMal	0.023 ± 0.005	0.038 ± 0.005
@pMal-NlgD	0.022 ± 0.006	0.037 ± 0.006

**FIGURE 8:**
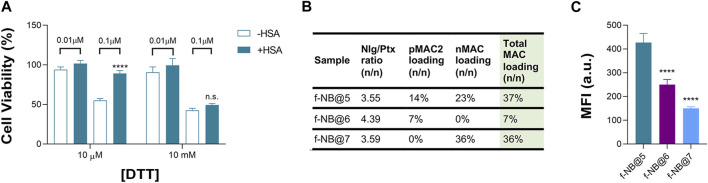
**(A)** Cell viability measured in MDA-MB-468 exposed for 48 h to NB@5, at 0.01 and 0.1 μM Ptx equivalent concentrations, pre-incubated or not with HSA before cells treatment. Data are expressed as mean ± SD of at least two independent experiments carried out in triplicate; data were analyzed using the 2-way ANOVA test, and Bonferroni’s multiple comparison as a post-test. Results were statistically significant for *p*-values < 0.05 (****p-values < 0.0001). **(B)** Fluorescent NBs (f-NB@ composition and MAC loading (n/n). **(C)** Intracellular uptake measured by FACS in MDA-MB-468 cells treated with fluorescent NBs for 2 h. Data are expressed as mean ± SD of at least two independent experiments carried out in triplicate; data were analyzed using the one-way ANOVA test and Tukey’s multiple comparison as post-test. Results were statistically significant at *p*-values < 0.05 (****p-values< 0.0001).

Overall, we speculated that the reduced toxicity of NB@5 with respect to MAC-devoid micelles could be related to the presence of the tEB moiety on the NBs’ surface and the consequent binding to serum albumins once diluted in cell culture medium (basal medium supplemented with 10% FBS). Indeed, as earlier shown under reducing conditions, the release of Ptx from pMAC2 in solution is strongly reduced in the presence of HSA (Figure 2). To further support this hypothesis, MDA-MB-468 cells were exposed to NB@5 nanoparticles that were pre-incubated with HSA or left untreated, in the presence of varying concentrations of DTT, i.e., 10 μM DTT and 10 mM DTT mimicking healthy and tumor tissues, respectively. At a Ptx equivalent concentration of 0.1 μM, the pre-incubation of NBs with HSA led to higher cell viability (80%) under mild reducing conditions (10 μM DTT, Figure 8A), strengthening the role of the protein in modulating the release of Ptx under physiological redox conditions. (Kennedy et al., 2020). Conversely, under compromised redox conditions (10 mM DTT), the protective effect of HSA was unremarkable (Figure 8A).

To gain more insight on the role of tEB on NBs protection and selectivity, we prepared fluorescently labeled NB formulations with similar Nlg/Ptx ratio as respect to NB@5 but with different MAC loading and incorporating the Nile Red (NR) fluorophore (f-NB@, Figure 8B and Supplementary Table S6). Our results indicate a pMAC2 loading-dependent intracellular uptake in MDA-MB-468 cells: the internalization rate of f-NB@6 and f-NB@7 (7% and 0% pMAC2 loading, respectively) was two and three folds lower compared to f-NB@5 (14% pMAC2 loading) (Figure 8C). These results suggest that the interaction of NBs with serum proteins is mainly mediated by pMAC2. Indeed, despite the similar MAC loading of f-NB@5 and f-NB@7, the latter exhibits less cellular internalization, likely due to the lower capacity of nMAC to stabilize NB-HSA interaction through the tEB moiety. This observation is consistent with the limited protection provided by HSA to nMAC, as observed in stability studies, in comparison to pMAC2.

**FIGURE 9:**
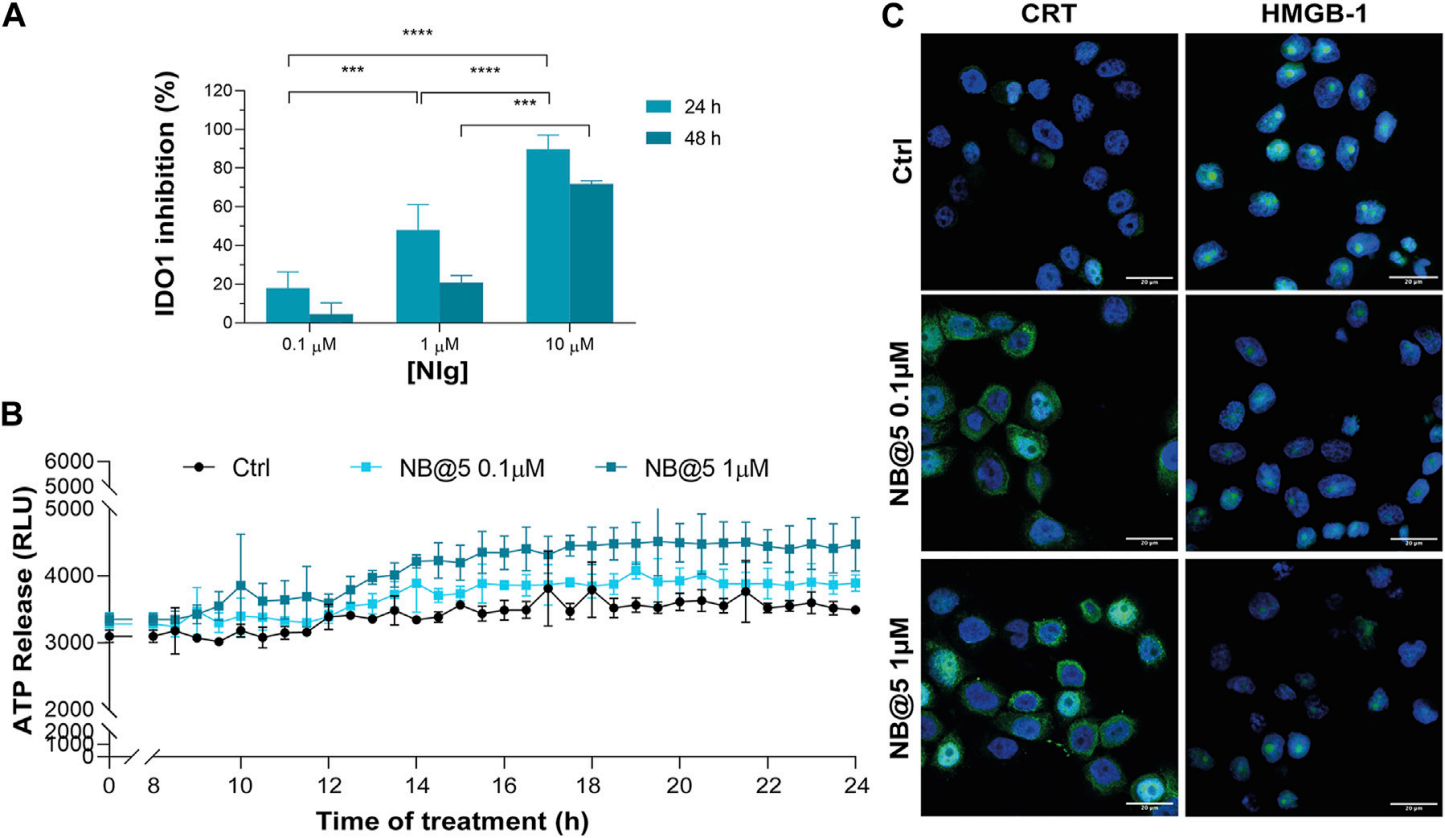
**(A)** IDO1 inhibition rate (%) in MDA-MB-468 cells incubated with different concentrations of Nlg incorporated into NB@5 for 24 h and 48 h **(B)** ATP release in the cell culture medium of cells exposed to NB@5 at Ptx equivalent concentrations of 0.1 and 1 µM for 24 h. **(C)** Immunofluorescence images of cells incubated with the compounds (1 µM Ptx) for 12 h and stained with antibody against CRT and HMGB1 FITC conjugated (green) and with Hoechst 33342 (blue) for nuclei recognition. Scale bar: 20 µm. Data are expressed as mean ± SD of at least 2 independent experiments carried out in triplicate; statistical analysis has been generated using the 2-way ANOVA test, and Tukey’s multiple comparison as a post-test. Results were considered statistically significant at *p*-values < 0.05 (* *p*-values < 0.05, ** *p*-values < 0.01, *** *p*-values < 0.001 and **** *p*-values < 0.0001).

Next, we evaluated whether NB@5 could successfully inhibit IDO1 enzymatic activity: our data confirmed a Nlg concentration-dependent inhibition of Kyn conversion, which was almost complete when MDA-MB-468 cells were incubated for 24 h with 10 μM equivalent of Nlg incorporated into NBs (Figure 9A). Finally, we investigated the capability of NB@5, at 0.1 and 1 μM Ptx equivalent concentrations, to drive ICD in TNBC cells. We measured a significant release of ATP in the extracellular media exclusively in MDA-MB-468 cells incubated with micelles containing 1 μM Ptx equivalent (Figure 9B), while in our experimental conditions, the amount of ATP released by MDA-MB-231 cells was not statistically different from control (not shown). Finally, our data confirmed both CRT membrane translocation and HMGB1 nuclear release in MDA-MB-468 (Figure 9C and Supplementary Figure S30) and MDA-MB-231 cells (Supplementary Figure S31) exposed to 1 μM Ptx equivalent for 12 h. These findings clearly demonstrate that NB@5 effectively trigger all the considered ICD hallmarks similarly to free Ptx, at least in MDA-MB-468 cells.

### 3.7 *In vitro* studies of NBs activity on TNBC cells spheroids

**FIGURE 10:**
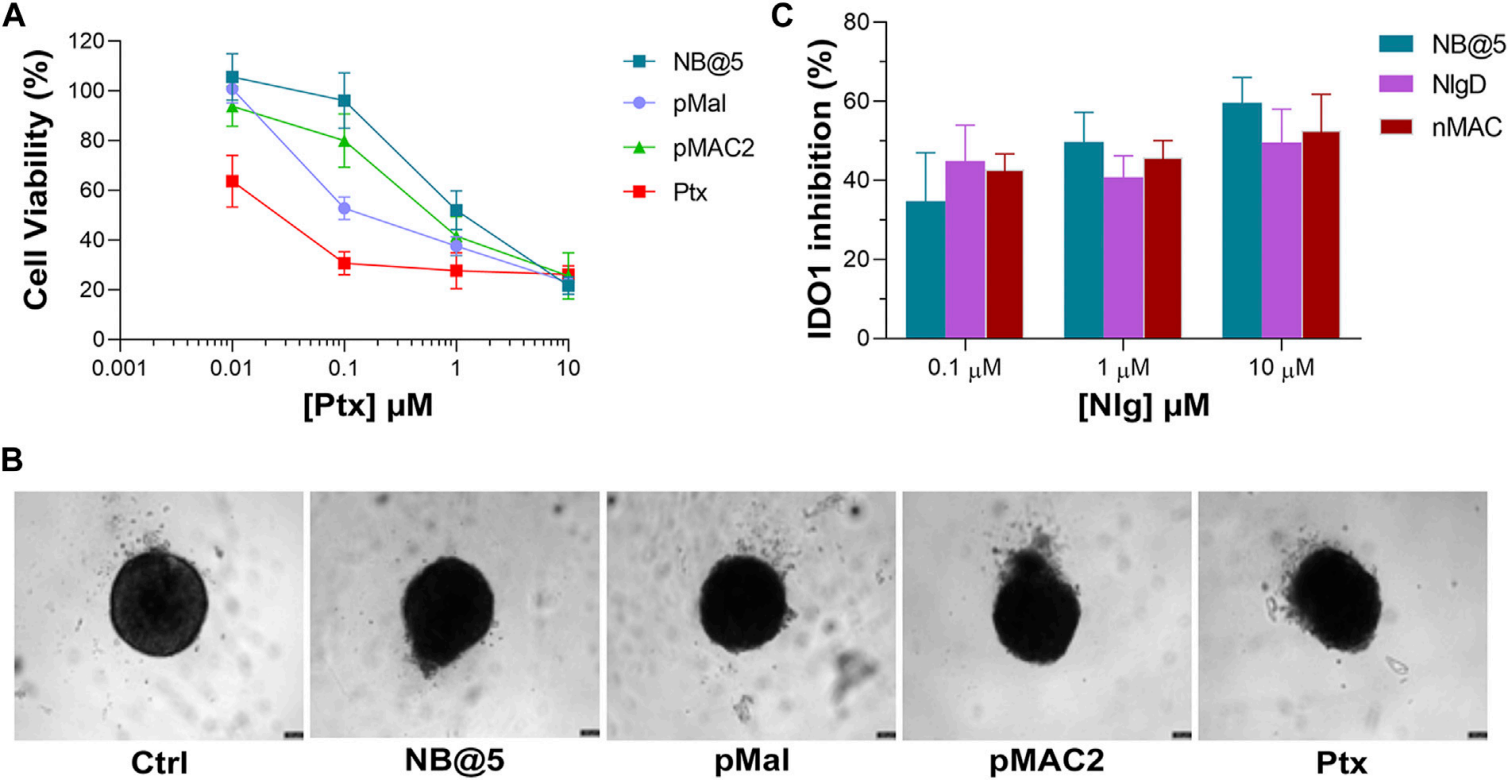
**(A)** Cytotoxicity measured in MDA-MB-468 spheroids treated for 72 h with NB@5 and reference compounds at increasing Ptx concentration and assessed with the CellTiter-Glo^®^ 3D assay. **(B)** Bright field images of treated spheroids at Ptx concentration corresponding to 1 µM. Scale bar: 100 µm. **(C)** IDO1 inhibition (%) calculated measuring Kyn content in the cell culture medium of cells exposed to drugs for 48 h.

To preliminary evaluate the anticancer and immunomodulating activity of NB@5 in an *in vitro* model resembling more closely *in vivo* solid tumors, we measured cell viability reduction and IDO1 inhibition activity in MDA-MB-468 cell-derived three-dimensional spheroids. Therefore, 4-days old spheroids were incubated for 72 h with Ptx formulations before assessing viability reduction using the 3D Glo assay. As depicted in Figure 10A, the cytotoxicity trend was similar to that observed in 2D cancer cell models, showing that at low Ptx concentrations, NB@5 and pMAC2 were significantly less toxic compared to Ptx and pMal. However, no significant differences in viability reduction were observed at higher Ptx concentrations. As evident from the bright field images of treated spheroids (Figure 10B), all treatments caused alterations in the morphological structure of the spheroids, leading to a loss of their spherical shape in comparison to the control group. However, the volume reduction was not statistically different (not shown). IDO1 inhibition rate was measured in spheroids exposed to NB@5 and Nlg prodrugs for 48 h. Our results indicate that both NB@5 and Nlg prodrugs effectively reduce IDO1 enzymatic activity (around 50%) in TNBC cells spheroids, although to a lesser extent as respect to 2D models and with less evident dose-inhibition dependency (Figure 10C).

## 4 Conclusion

Targeting IDO1 in conjunction with ICD-inducing chemotherapeutic agents represents a promising opportunity for enhancing both the initiation and sustenance of a tumor-specific T-cell immune response within the TME. However, critical factors must be considered when approaching combination therapy, such as uneven drug accumulation at the target tissue and premature drug release. To address these challenges, the use of inactive prodrugs encapsulated within well-designed nanocarriers offers distinct advantages, including protection against degradation, prolonged circulation time, improved pharmacokinetics, sustained drug release, and the ability to deliver insoluble drugs. Our study introduces the synthesis of innovative HSA-binding and bioresponsive prodrugs, forming stable carrier-free nanobinders with adjustable drug ratios and serum albumin binding functionalization. Our research data support the hypothesis that NB@5 effectively binds with HSA, showcasing its protective role in the controlled release of drugs *in vitro*. This study suggests that the developed nanobinders can exploit HSA as an endogenous shuttle for targeted delivery to the tumor site *in vivo*. Additionally, our study demonstrates that drugs encapsulated in the nanobinders are selectively released under the altered redox conditions typical of the TME, inducing cell death, promoting ICD, and inhibiting IDO1 under the tested conditions.

Nevertheless, although we preliminary assessed NBs’ cytotoxicity, ICD-inducing capability, and IDO1 inhibition efficacy, additional *in vitro* tests on immune cells are necessary to fully elucidate the system’s potential. Moreover, given the limitations of *in vitro* settings, especially for evaluating complex molecular pathways within the entire tumor microenvironment and the exploitation of HSA as the natural carrier of NBs, a preclinical *in vivo* assessment will be crucial to solidify the potential of our proposed approach.

## Data Availability

The original contributions presented in the study are included in the article/Supplementary Material, further inquiries can be directed to the corresponding authors.
