# Corrigendum: HSA-nanobinders crafted from bioresponsive prodrugs for combined cancer chemoimmunotherapy–an *in vitro* exploration

**DOI:** 10.3389/fchem.2024.1443822

**Published:** 2024-06-18

**Authors:** Matilde Tubertini, Luca Menilli, Celeste Milani, Cecilia Martini, Maria Luisa Navacchia, Marta Nugnes, Manuela Bartolini, Marina Naldi, Daniele Tedesco, Elisa Martella, Andrea Guerrini, Claudia Ferroni, Francesca Moret, Greta Varchi

**Affiliations:** ^1^ Institute for Organic Synthesis and Photoreactivity (ISOF), National Research Council of Italy (CNR), Bologna, Italy; ^2^ Department of Science and High Technology, University of Insubria, Como, Italy; ^3^ Pharmacy Unit, Veneto Institute of Oncology IOV-IRCSS, Padua, Italy; ^4^ Department of Biology (DiBio), University of Padova, Padua, Italy; ^5^ Department of Pharmacy and Biotechnology (FaBiT), University of Bologna, Bologna, Italy

**Keywords:** prodrugs, chemotherapy, IDO1 inhibition, endogenous human serum albumin, carrier-free nanoparticles, truncated evans blue, triple-negative breast cancer

In the published article, there was an error in the **Affiliation**. Matilde Tubertini’s second affiliation was missing. The correct affiliations appear above.

The authors apologize for this error and state that this does not change the scientific conclusions of the article in any way. The original article has been updated.

