# Supplementary material for: HSA-nanobinders crafted from bioresponsive prodrugs for combined cancer chemoimmunotherapy–an in vitro exploration
Source: Front Chem. Author manuscript; Available in PMC 2024 Apr 8. (PMC7615814; doi:10.3389/fchem.2024.1378233)
Supplement: Supplementary material [file EMS195136-supplement-Supplementary_material.PDF]

Supplementary Material

HSA-nanobinders crafted from bioresponsive prodrugs for combined cancer chemoimmunotherapy – an in vitro exploration

Matilde Tubertini[1‡], Luca Menilli[2‡], Celeste Milani[3‡], Cecilia Martini[1], Maria Luisa Navacchia[1], Marta Nugnes[4], Manuela Bartolini[4], Marina Naldi[4], Daniele Tedesco[1], Elisa Martella[1], Andrea Guerrini[1], Claudia Ferroni[1], Francesca Moret[3*], Greta Varchi[1*]

*** Correspondence:** Corresponding Authors: greta.varchi@isof.cnr.it; francesca.moret@unipd.it

Table of content

1 Synthesis and characterization ... 3
1.1 NLG919 dimer with disulfide linker (NlgD) ... 3
1.2 Truncated Evans Blue (tEB) ... 3
1.2.1 Compound A ... 3
1.2.2 Compound B ... 3
1.2.3 Truncated Evans Blue (tEB) ... 4
1.3 Compound 1 ... 4
1.4 Compound 2 ... 5
1.5 Compound 3 ... 5
1.6 pMAC1 ... 6
1.7 pMal ... 6
1.8 Compound 4 ... 6
1.9 Derivative 5 ... 7
1.9.1 Compound D ... 7
1.9.2 Compound E ... 7
1.9.3 Compound 5 ... 8
1.9.4 Derivative 6 ... 8
1.10 pMAC2 ... 8
1.11 Compound 7 ... 9
1.12 Compound 8 ... 9
1.13 Compound 9 ... 9
1.14 nMAC ... 10
1.15 HPLC-MS analyses ... 10
1.16 Sensorgrams ... 11
2 NMR and LC-MS Spectra ... 12
3 HPLC Analysis ... 25
3.1 UV spectra of drugs and prodrugs ... 25
3.2 Prodrug stability over time ... 26
3.3 Stereoisomers of Ptx and Nlg ... 26
3.4 HPLC calibration for Ptx and Nlg ... 27
3.5 Ptx and Nlg release from NB@5 ... 28
4 Nanobinders preparation and characterization ... 29
5 In vitro studies on TNBC cell monolayers ... 31
6 References ... 33

List of Supplementary Figures

Supplementary Figure 1. SPR analysis ... 11
Supplementary Figure 2. ^{1}H-NMR spectrum of NlgD ... 13
Supplementary Figure 3. ^{1}H-NMR spectrum of compound **2** ... 13
Supplementary Figure 4. ^{1}H-NMR spectrum of compound **3** ... 14
Supplementary Figure 5. ^{1}H-NMR spectrum of pMAC1 ... 15
Supplementary Figure 6. LC-MS analysis of pMAC1 ... 15
Supplementary Figure 7. ^{1}H-NMR spectrum of pMal ... 16
Supplementary Figure 8. ^{13}C-NMR spectrum of pMal ... 17
Supplementary Figure 9. ^{1}H-NMR spectrum compound **4** ... 17
Supplementary Figure 10. ^{1}H-NMR spectrum of compound **5** ... 18
Supplementary Figure 11. ^{1}H-NMR spectrum of compound **6** ... 18
Supplementary Figure 12. ^{13}C-NMR spectrum of compound **6** ... 19
Supplementary Figure 13. ^{1}H-NMR spectrum of pMAC2 ... 20
Supplementary Figure 14. LC-MS analysis of pMAC2 ... 20
Supplementary Figure 15. ^{1}H-NMR spectrum of compound **7** ... 21
Supplementary Figure 16. ^{13}C-NMR spectrum compound **7** ... 22
Supplementary Figure 17. ^{1}H-NMR spectrum of compound **8** ... 22
Supplementary Figure 18. ^{1}H-NMR spectrum of compound **9** ... 23
Supplementary Figure 19. ^{13}C-NMR spectrum of compound **9** ... 23
Supplementary Figure 20. ^{1}H-NMR spectrum of compound nMAC ... 24
Supplementary Figure 21. LC-MS analysis of nMAC ... 24
Supplementary Figure 22. PDA-extracted UV spectra ... 25
Supplementary Figure 23. Stability of pMal and MACs ... 26
Supplementary Figure 24. Main stereoisomers of Ptx and Nlg. ... 26
Supplementary Figure 25. HPLC-UV calibration curves ... 27
Supplementary Figure 26. TEM analysis of @pMal-NlgD pre-incubated with HSA ... 30
Supplementary Figure 27. Viability of MDA-MB-231 cells; IDO1 inhibition in MDA-MB-468 cells ... 31
Supplementary Figure 28. CRT/HMGB1 signals after treatment of MDA-MB-468 cells with Ptx and pMAC2 ... 31
Supplementary Figure 29. Cytotoxicity in MSCs cells ... 31
Supplementary Figure 30. CRT/HMGB1 signals after treatment of MDA-MB-468 cells with NB@5 ... 32
Supplementary Figure 31. Immunofluorescence and CRT/HMGB1 signals after treatment of MDA-MB-231 cells with NB@5 ... 32

List of Supplementary Schemes

Supplementary Scheme 1. Synthesis of truncated Evans Blue (tEB) ... 3
Supplementary Scheme 2. Synthesis of compound 1. ... 4
Supplementary Scheme 3. Synthesis of derivative 5. ... 7

List of Supplementary Tables

Supplementary Table 1. HPLC calibration parameters and limits ... 27
Supplementary Table 2. HPLC analysis on the release of Ptx and Nlg from NB@5 ... 28
Supplementary Table 3. Experimental details for the preparation of MAC-devoid micelles ... 29
Supplementary Table 4. Experimental details for the preparation of selected pMAC1-based NBs ... 29
Supplementary Table 5. Experimental details for the preparation of selected pMAC2-based NBs ... 29
Supplementary Table 6. Experimental details for the preparation of fluorescently labelled NB formulations ... 29

1 Synthesis and characterization

1.1 NLG919 dimer with disulfide linker (NlgD)

NlgD was synthesized in a one-pot reaction, as reported in literature (1). Nlg (1 equiv.) was dissolved in anhydrous DCM (5.7 mL/mmol) in a two-necked round-bottomed flask under nitrogen atmosphere. The flask was placed in an ice bath and cooled to 0 °C, and DMAP (3.5 equiv.) was added to the reaction mixture. Subsequently, a solution of triphosgene (0.4 equiv.) in dry DCM (3.6 mL/mmol) was added dropwise and the reaction stirred at 0 °C for 2 h. Next, a solution of 2-hydroxyethyldisulfide (0.4 equiv.) in DCM (1.4 mL/mmol) was added, and the reaction mixture was cooled to 4 °C and stirred overnight. Then, the mixture was gradually warmed to r.t. and treated with HCl (0.1 N in H_2O) and washed with H_2O (3 x 2 mL). The combined organic layers were dried over anhydrous sodium sulfate (Na_2SO_4), filtered and the solvent removed under reduced pressure and the crude product was purified by flash chromatography (eluent: DCM/cHex/MeOH 10:1:0.2 v/v/v), affording pure NlgD as a white crystalline powder in 40% yield. ^{1}H-NMR (400 MHz, $CDCl_3$;

Supplementary Figure 2): δ 7.71 (dd, J = 1.5, 0.7 Hz, 2H), 7.56 – 7.47 (m, 4H), 7.36 (td, J = 7.5, 1.0 Hz, 2H), 7.28 – 7.20 (m, 2H), 7.18 (s, 2H), 5.18 (dd, J = 7.4, 4.1 Hz, 2H), 4.76 (m, 2H), 4.35 – 4.18 (m, 4H), 2.89 (t, J = 6.6 Hz, 4H), 2.48 – 2.36 (m, 2H), 2.16 (m, 2H), 2.04 – 1.99 (m, 1H), 1.78 – 1.59 (m, 10H), 1.51 (m, 2H), 1.15 (m, 6H), 1.00 (m, 4H).

1.2 Truncated Evans Blue (tEB)

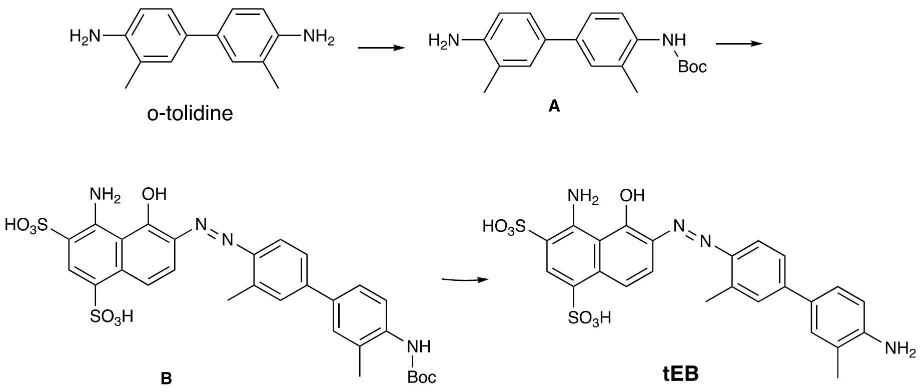

Supplementary Scheme 1. Synthesis of truncated Evans Blue (tEB).

1.2.1 Compound A

In a round-bottomed flask, o-tolidine (1 equiv.) was dissolved in anhydrous DCM (2 mL/mmol) under stirring. After the complete dissolution, *tert*-butoxycarbonyl anhydride (1 equiv.) was added to the solution and the reaction mixture was left for 24 h under stirring at r.t.. The crude product was then purified by flash chromatography (eluent: cHex/EtOAc; 2:1 v/v), leading to compound **I** in 68% yield. ^{1}H-NMR (400 MHz, $CDCl_3$): δ 7.80 (d, J = 8.3 Hz, 1H), 7.38 – 7.36 (m, 1H), 7.36 – 7.34 (m, 1H), 7.32 (dt, J = 2.3, 0.6 Hz, 1H), 7.29 – 7.23 (m, 3H), 6.73 (dt, J = 7.9, 0.5 Hz, 1H), 6.26 (s, 1H), 3.79 (s, 2H), 2.29 (s, 3H), 2.23 (s, 3H), 1.54 (s, 9H).

1.2.2 Compound B

Compound **A** (1 equiv.) was dissolved in ACN (6.9 mL/mmol) and cooled at 0 °C. A cold (0 °C) solution of HCl (0.3 N in uH_2O; 10.2 mL/mmol **A**) was then added followed by a cold (0 °C) solution of $NaNO_2$ (3 equiv.) dissolved in H_2O (2.8 mL/mmol $NaNO_2$). The reaction mixture, whose colour

changed from pink to yellow, was kept under stirring a 0 °C for 30 min. In a second flask, 1-amino-8-naphtol-2,4-disulfonic acid (1.1 equiv.) was added to a stirred solution of $NaHCO_3$ (3.6 equiv.) dissolved in uH_2O (0.57 mL/mmol) at 0 °C and stirred for 30 min. At this point, the first solution was slowly added to the flask and stirred for 3 h at 10 °C (violet solution). The reaction mixture was then freeze-dried to remove H_2O and ACN; the dry powder was dissolved in a tiny amount of uH_2O and MeOH, transferred into a cellulose dialysis bag (MWCO: 14 kDa) and dialyzed against excess uH_2O (100:1 v/v) for 24 h, with the dialysate replaced every 6 h with fresh uH_2O to remove salts. Upon dialysis, compound **B** was obtained as a violet solid in 61% yield. ^{1}H-NMR (500 MHz, D_2O): δ 8.40 (s, 1H), 7.63 (d, *J* = 9.7 Hz, 1H), 7.44 – 7.26 (m, 5H), 7.20 (s, 1H), 7.05 (d, *J* = 9.8 Hz, 1H), 6.88 (d, *J* = 8.2 Hz, 1H), 2.24 (s, 3H), 2.09 (s, 3H), 1.46 (s, 9H).

1.2.3 Truncated Evans Blue (tEB)

Unlike what reported in the literature, (2) a microwave enabled procedure was applied to the deprotection of compound **B.** Compound **B** (1 equiv.) was dissolved in H_2O (3 mL/mol) and inserted into a microwave oven at 300 W with an internal pressure of 5 bar for 8 min; pure tEB was obtained in quantitative yield as a dark red powder upon freeze drying. ^{1}H-NMR (400 MHz, CD_3OD): δ 8.70 (s, 1H), 7.99 (d, *J* = 9.9 Hz, 1H), 7.91 (d, *J* = 8.5 Hz, 1H), 7.52 (dd, *J* = 8.6, 2.0 Hz, 1H), 7.47 (d, *J* = 1.8 Hz, 1H), 7.35 – 7.25 (m, 3H), 7.17 (d, *J* = 9.8 Hz, 1H), 6.76 (d, *J* = 8.2 Hz, 1H), 2.53 (s, 3H), 2.22 (s, 3H).

1.3 Compound 1

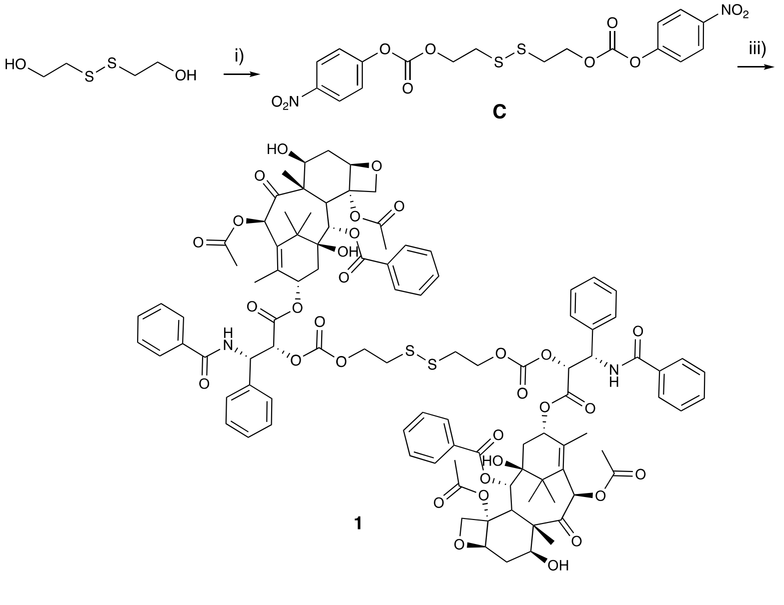

Supplementary Scheme 2. Synthesis of compound **1**.

2-Hydroxyethyl disulfide (1 equiv.) was added to a two-necked round-bottomed flask under nitrogen atmosphere and dissolved with dry DCM (1.15 mL/mmol). p-Nitrophenyl chloroformate was then added (3 equiv.) followed by *N*,*N*-diisopropylethylamine (DIPEA; 2.5 equiv.). The reaction mixture was stirred at r.t. for 5 h and monitored by TLC. Once complete, the solution was transferred into a separatory funnel and washed with HCl (0.1 N in H_2O), a saturated aqueous solution of sodium bicarbonate ($NaHCO_3$) and brine. The organic layer was recovered and dried over magnesium sulfate ($MgSO_4$). The crude compound was purified by flash chromatography (eluent: cHex/EtOAc 2:1, v/v),

affording derivative **C** as a yellow crystalline powder in 83% yield. ^{1}H-NMR (400 MHz, $CDCl_3$) δ 8.28 (d, *J* = 9.3 Hz, 4H), 7.40 (d, *J* = 7.1 Hz, 4H), 4.57 (t, *J* = 6.5 Hz, 4H), 3.08 (t, *J* = 6.5 Hz, 4H).

Compound **C** (1 equiv.) was added to a two-necked round-bottomed flask under nitrogen atmosphere and dissolved with dry DCM (18.2 mL/mmol). Afterwards, Ptx (2.09 equiv.) was added to the solution followed by DMAP (2.18 equiv.) After stirring at r.t. for 4 h, the reaction mixture was transferred into a separatory funnel and washed with HCl (0.1 N in uH_2O), saturated $NaHCO_3$ and brine. The organic layer was recovered and dried over dry $MgSO_4$. The crude compound was purified by flash chromatography (eluent: EtOAc/cHex 2:1 v/v → EtOAc/cHex/MeOH 3:1:0.1 v/v/v), affording pure compound **1** as a white crystalline powder in 90% yield. ^{1}H-NMR (400 MHz, $CDCl_3$): δ 8.17 – 8.12 (m, 4H), 7.75 – 7.69 (m, 4H), 7.65 – 7.57 (m, 2H), 7.55 – 7.32 (m, 20H), 6.97 (d, *J* = 9.4 Hz, 2H), 6.31 – 6.23 (m, 4H), 6.00 (dd, *J* = 9.4, 2.8 Hz, 2H), 5.69 (d, *J* = 7.1 Hz, 2H), 5.44 (d, *J* = 2.8 Hz, 2H), 4.97 (dd, *J* = 9.7, 2.3 Hz, 2H), 4.47 – 4.28 (m, 8H), 4.20 (dd, *J* = 8.4, 1.0 Hz, 2H), 3.81 (d, *J* = 7.0 Hz, 2H), 2.88 (t, *J* = 6.5 Hz, 4H), 2.62 – 2.34 (m, 12H), 2.22 (s, 6H), 1.94 – 1.83 (m, 6H), 1.68 (s, 6H), 1.61 (s, 6H), 1.23 (d, *J* = 4.6 Hz, 6H), 1.14 (s, 6H).

1.4 Compound 2

Commercially available o-tolidine (1 equiv.) was added to solution of methyl 3,4-dibromo-2,5-dioxocyclopent-3-ene-1-carboxylate in dry dichloromethane (DCM; 37.5 mL/mmol) under nitrogen atmosphere. After stirring at room temperature (r.t.) for 4 h, the solvent was evaporated under vacuum and the crude material was purified by flash chromatography using a cyclohexane/ethyl acetate mixture (cHex/EtOAc 3:5 v/v) as the eluent, affording pure compound **4** as a red crystalline solid in 45% yield. ^{1}H-NMR (400 MHz, $CDCl_3$; Supplementary Figure 3): δ 7.48 (d, *J* = 2.1 Hz, 1H), 7.44 (dd, *J* = 8.2, 2.1 Hz, 1H), 7.33 – 7.25 (m, 2H), 7.13 (d, *J* = 8.1 Hz, 1H), 6.74 (d, *J* = 8.0 Hz, 1H), 3.65 (s, 2H), 2.24 (s, *3*H), 2.21 (s, *3*H).

1.5 Compound 3

Compound **1** (1 equiv.) was introduced into a two-necked round-bottomed flask under argon atmosphere and dissolved with dry tetrahydrofuran (THF; 20 mL/mmol). Compound **2** (2. equiv.), dissolved into a THF/MeOH mixture (1:3 v/v; 150 mL/mmol), was then added to the resulting solution followed by tris(2-carboxyethyl) phosphine hydrochloride (TCEP·HCl; 2.5 equiv.) and *N*,*N*-diisopropylethylamine (DIPEA; 6 equiv.). The reaction was monitored by TLC (eluent: cHex/EtOAc/MeOH 6:8:0.1 v/v/v) and was considered complete after stirring at r.t. for 1.5 h. The crude mixture was then transferred into a separatory funnel and washed with hydrochloric acid (HCl; 0.1 N in H_2O) followed by brine and extracted with DCM. The collected organic layer was dried over magnesium sulphate ($MgSO_4$), filtered and concentrated under vacuum. The crude was purified by flash chromatography (eluent: cHex/EtOAc 6:9 v/v → 7:9 v/v) affording pure compound **3** in 65% yield. ^{1}H-NMR (400 MHz, $CDCl_3$; Supplementary Figure 4): δ 8.18 – 8.09 (m, 4H), 7.78 – 7.69 (m, 4H), 7.64 – 7.56 (m, 2H), 7.55 – 7.32 (m, 20H), 7.31 – 7.26 (m, 2H), 7.10 (dd, *J* = 14.7, 8.1 Hz, 1H), 6.91 (dd, *J* = 9.3, 2.2 Hz, 2H), 6.76 (dd, *J* = 8.0, 1.6 Hz, 1H), 6.28 (d, *J* = 9.6 Hz, 4H), 6.01 (dt, *J* = 9.4, 2.3 Hz, 2H), 5.69 (d, *J* = 7.0 Hz, 2H), 5.45 (t, *J* = 2.8 Hz, 2H), 4.99 – 4.93 (m, 2H), 4.55 – 4.39 (m, 6H), 4.36 – 4.28 (m, 2H), 4.21 (d, *J* = 8.4 Hz, 2H), 3.81 (t, *J* = 7.6 Hz, 2H), 3.76 – 3.62 (m, 4H), 2.61 – 2.49 (m, 2H), 2.45 (d, *J* = 3.8 Hz, 6H), 2.43 – 2.36 (m, 2H), 2.27 – 2.20 (m, 13H), 2.19 (s, 2H), 2.17 (s, 2H), 1.95 – 1.90 (m, 6H), 1.88 (dd, *J* = 4.0, 2.4 Hz, 2H), 1.79 (d, *J* = 1.4 Hz, 1H), 1.68 (s, 6H), 1.24 (s, 6H), 1.14 (s, 6H). MS (ES+): m/z calcd for $C_{118}H_{122}N_4O_{34}S_2$+$H^+$ 2203.75 $[M+H]^+$; found 2203.31, 2224.95 $[M+Na]^+$.

1.6 pMAC1

Compound **3** (1 equiv.) was dissolved in a glass vial with ACN (0.028 mL/mmol) and cooled at 0 °C; then, HCl (0.3 M in uH_2O; 3 equiv.) was added dropwise followed by a cold solution of sodium nitrite ($NaNO_2$) in uH_2O (6.1 mL/mmol $NaNO_2$). After stirring for 30 min, the solution was added dropwise to another glass vial, containing 1-amino-8-naphthol-2,4-disulfonic acid monosodium salt (1.1 equiv.) and sodium bicarbonate ($NaHCO_3$; 4 equiv.) dissolved in uH_2O (3.3 mL/mmol $NaHCO_3$), and kept at 0 °C; reaction completion (3 h) was followed by TLC. After the disappearance of the starting compound **3**, ACN was removed under vacuum; the residual aqueous solution was transferred into a cellulose dialysis bag (Sigma-Aldrich, Merck, Italy) with a 14 kDa molecular weight cut-off (MWCO) and dialyzed against excess uH_2O (100:1 v/v) for 24 h, with the dialysate replaced every 6 h with fresh uH_2O. The remaining solution was freeze-dried and purified by flash chromatography (eluent: acetone/uH_2O 30:1 v/v → 15:1 v/v) after preliminary column conditioning with acetone. The collected compound was further washed with a diethyl ether/DCM mixture (Et_2O/DCM; 2:1 v/v) to obtain pure compound pMAC1 as a dark purple powder in 70% yield. ^{1}H-NMR (500 MHz, CD_3OD; Supplementary Figure 5): δ 8.72 (s, 1H), 8.11 (dt, *J* = 8.6, 1.6 Hz, 4H), 8.04 – 7.98 (m, 2H), 7.83 – 7.77 (m, 4H), 7.70 – 7.55 (m, 9H), 7.50 (dd, *J* = 7.9, 3.4 Hz, 7H), 7.47 – 7.38 (m, 7H), 7.26 (td, *J* = 7.5, 1.5 Hz, 2H), 7.17 (d, *J* = 9.9 Hz, 1H), 6.44 (s, 2H), 6.10 (d, *J* = 10.4 Hz, 2H), 5.84 (d, *J* = 6.3 Hz, 2H), 5.63 (dd, *J* = 7.3, 2.2 Hz, 2H), 5.49 (dd, *J* = 6.4, 3.0 Hz, 2H), 4.99 (t, *J* = 6.5 Hz, 2H), 4.60 (s, 2H), 4.50 (dt, *J* = 11.5, 5.8 Hz, 2H), 4.34 (dd, *J* = 11.0, 6.7 Hz, 2H), 4.18 (s, 4H), 3.84 – 3.77 (m, 2H), 3.72 – 3.62 (m, 3H), 3.62 – 3.48 (m, 5H), 2.56 (s, 3H), 2.39 (s, 3H), 2.38 (s, 3H), 2.24 – 2.17 (m, 7H), 2.16 (s, 9H), 1.96 – 1.92 (m, 6H), 1.88 – 1.74 (m, 4H), 1.64 (s, 6H), 1.29 (s, 6H), 1.11 (d, *J* = 9.9 Hz, 6H). MS (ES+): m/z calcd for $C_{128}H_{128}N_6O_{41}S_4$+$H^+$ 2533.71 $[M+H]^+$; found 2533.59 (Supplementary Figure 6).

1.7 pMal

Compound **1** (1 equiv.) was added to a two-necked round-bottomed flask under nitrogen atmosphere and dissolved with dry THF (11.5 mL/mmol). 2,3-Dibromomaleimide (1.1 equiv.) dissolved in MeOH (34 mL/mmol) was then added, followed by TCEP·HCl (2 equiv.) and DIPEA (10 equiv.). After stirring at r.t. for 3 h, the reaction mixture was transferred into a separatory funnel and washed with HCl (0.1 N in uH_2O), a saturated aqueous solution of ammonium chloride (NH_4Cl) and brine. The organic layer was recovered and dried over $MgSO_4$. The crude compound was purified by flash chromatography (eluent: EtOAc/DCM 3:2 v/v) affording pure pMal as a yellow crystalline powder in 71% yield. ^{1}H-NMR (400 MHz, $CDCl_3$; Supplementary Figure 7): δ 8.16 – 8.10 (m, 4H), 7.77 – 7.71 (m, 4H), 7.65 – 7.57 (m, 2H), 7.57 – 7.31 (m, 21H), 7.03 (d, *J* = 9.3 Hz, 2H), 6.35 – 6.18 (m, 4H), 5.97 (dd, *J* = 9.3, 3.2 Hz, 2H), 5.67 (d, *J* = 7.2 Hz, 2H), 5.42 (d, *J* = 3.2 Hz, 2H), 4.97 (dd, *J* = 9.7, 2.2 Hz, 2H), 4.42 (dd, *J* = 10.9, 6.8 Hz, 2H), 4.38 – 4.27 (m, 6H), 4.18 (d, *J* = 8.5 Hz, 2H), 3.78 (d, *J* = 7.1 Hz, 2H), 3.49 (ddt, *J* = 52.2, 14.2, 6.2 Hz, 4H), 2.62 – 2.49 (m, 2H), 2.44 (s, 6H), 2.41 – 2.30 (m, 3H), 2.22 (d, *J* = 13.1 Hz, 8H), 1.89 (s, 8H), 1.68 (d, *J* = 2.6 Hz, 8H), 1.42 (s, 2H), 1.22 (s, 6H), 1.14 (d, *J* = 3.4 Hz, 6H). ^{13}C-NMR (101 MHz, $CDCl_3$; Supplementary Figure 8): δ 203.67, 192.12, 187.11, 179.45, 173.24, 171.24, 167.82, 167.07, 164.85, 156.46, 154.86, 153.81, 142.47, 136.59, 135.89, 133.70, 132.06, 130.21, 129.15, 128.71, 127.19, 126.68, 112.39, 81.12, 79.14, 75.02, 72.13, 67.83, 58.47, 52.71, 43.16, 35.60, 29.60, 26.79, 22.02, 20.81, 9.59.

1.8 Compound 4

pMal (1 equiv.) was added to a two-necked round-bottomed flask under nitrogen atmosphere and dissolved with dry THF (9 mL/mmol). *N*-methylmorpholine (NMM) (1.5 equiv.) was added to the

solution followed by methyl chloroformate (1.5 equiv.). After stirring at r.t. for 4 h, the reaction mixture was transferred into a separatory funnel and washed with uH_2O and brine. The organic layer was recovered and dried over $MgSO_4$. The crude compound was purified by flash chromatography (eluent: EtOAc/DCM 2:3 v/v) affording pure compound **4** as a yellow sticky oil in 99% yield. ^{1}H-NMR (400 MHz, $CDCl_3$; Supplementary Figure 9): δ 8.22 – 8.05 (m, 4H), 7.78 – 7.70 (m, 4H), 7.64 – 7.56 (m, 2H), 7.44 – 7.34 (m, 18H), 7.01 (d, *J* = 9.5 Hz, 2H), 6.31 – 6.21 (m, 4H), 6.00 (dd, *J* = 9.3, 3.2 Hz, 2H), 5.68 (d, *J* = 7.3 Hz, 2H), 5.43 (t, *J* = 4.0 Hz, 2H), 5.30 (s, 1H), 4.97 (d, *J* = 9.5 Hz, 2H), 4.41 (dq, *J* = 17.6, 6.2 Hz, 4H), 4.30 (t, *J* = 6.0 Hz, 4H), 4.20 (d, *J* = 7.6 Hz, 2H), 3.88 – 3.70 (m, 8H), 3.64 – 3.47 (m, 4H), 2.55 (ddd, *J* = 15.1, 9.5, 6.3 Hz, 2H), 2.51 – 2.35 (m, 8H), 2.24 (d, *J* = 3.7 Hz, 2H), 2.20 (s, 4H), 1.87 (dt, *J* = 18.5, 2.4 Hz, 12H), 1.68 (d, *J* = 2.9 Hz, 6H), 1.23 (s, 6H), 1.13 (s, 6H).

1.9 Derivative 5

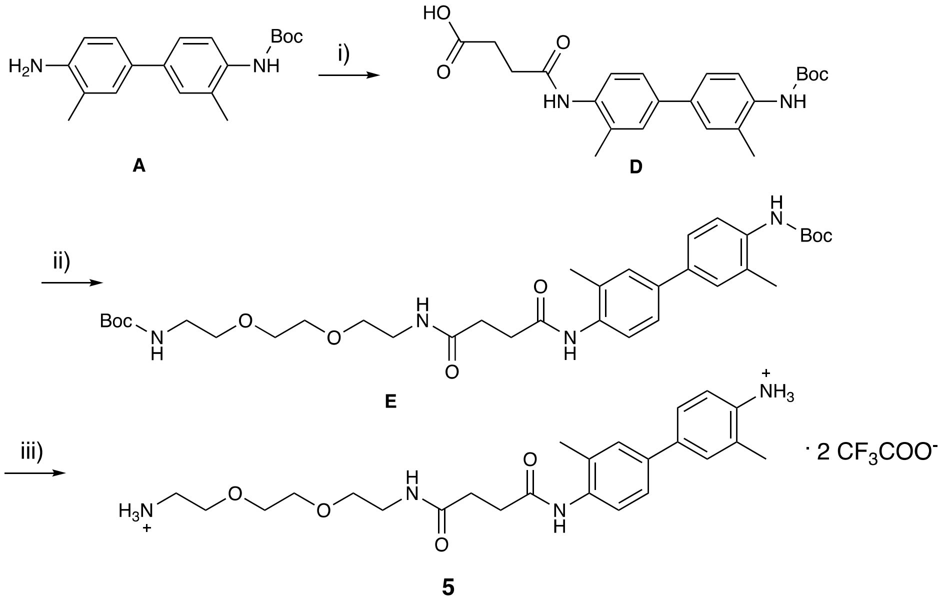

Supplementary Scheme 3. Synthesis of derivative **5**.

1.9.1 Compound D

Succinic anhydride (15 equiv.) was added to a solution of compound **A** (3) in anhydrous THF (15 mL/mmol) at r.t. and under inert atmosphere. DIPEA (10 equiv.) was then added dropwise. After stirring overnight, the reaction mixture was washed with a saturated solution of NH_4Cl and with uH_2O; the organic phase was then extracted with EtOAc, and the organic layer was recovered and dried over $MgSO_4$. The crude compound was purified by flash chromatography (eluent: DCM/MeOH 95:5 v/v), affording pure compound **D** as a yellow sticky oil in 55% yield. ^{1}H-NMR (400 MHz, CD_3OD): δ 7.45-7.42 (m, 6H), 2.71 (dd, *J* = 5.7, 3.9 Hz, 4H), 2.31 (s, 3H), 2.31 (s, 3H), 1.53 (s, 9H).

1.9.2 Compound E

Compound **D** (1 equiv.) and *N*-Boc-PEG$_2$-NH$_2$ (4 equiv.) (4) were added to a two-necked round-bottomed flask under nitrogen atmosphere and dissolved with dry THF (27 mL/mmol). DIPEA (15 equiv.) and (benzotriazol-1-yloxy)tri-pyrrolidinophosphonium hexafluorophosphate (PyBOP; 2.2 equiv.) were then added to the reaction mixture, which was subsequently stirred at r.t. for 3.5 h (monitored by TLC). The reaction mixture was transferred into a separatory funnel, washed three times with HCl (0.1 N in H_2O) and purified by flash chromatography (eluent: DCM/MeOH 20:1 v/v → 15:1 v/v → 1/1 v/v), affording pure compound **V** as a yellow sticky oil in 90% yield. ^{1}H-NMR (400 MHz,

$CDCl_3$): δ 7.88 (dd, *J* = 25.6, 8.1 Hz, 2H), 7.43 – 7.32 (m, 4H), 6.28 (bs, 2H), 3.59 – 3.47 (m, 8H), 3.31 – 3.23 (m, 4H), 2.76 – 2.67 (m, 4H), 2.33 (s, 3H), 2.30 (s, 3H), 1.54 (s, 9H), 1.44 (s, 9H).

1.9.3 Compound 5

Compound **E** (1 equiv.) was dissolved in DCM (20 mL/mmol) and TFA (30 mL/mmol) was added followed by triisopropylsilane (TIPS; 3 mL/mmol) at 0 °C under nitrogen atmosphere. The reaction was monitored by TLC (eluent: DCM/MeOH 20:1 v/v) showing an almost complete disappearance of the starting material after 2 h at r.t. The solvent was then removed under vacuum and the residue was treated with a Et_2O/n-pentane mixture (2:1 v/v) to allow the precipitation of compound **5**, which was recovered by filtration and vacuum drying in quantitative yield. ^{1}H-NMR (400 MHz, CD_3OD; Supplementary Figure 10): δ 7.41 – 7.10 (m, 6H), 3.40 (d, *J* = 11.1 Hz, 14H), 3.31 (t, *J* = 5.6 Hz, 2H), 3.14 (t, *J* = 5.6 Hz, 2H), 2.83 (t, *J* = 5.0 Hz, 2H), 2.50 (t, *J* = 6.8 Hz, 2H), 2.35 (t, *J* = 6.9 Hz, 2H), 2.20 (s, 3H), 2.07 (s, 3H).

1.9.4 Derivative 6

Compound **5** (1.5 equiv.) was added to a two-necked round-bottomed flask under nitrogen atmosphere and dissolved with dry DCM (5 mL/mmol). DIPEA (8 equiv.) was then added dropwise, followed by a solution of compound **4** dissolved in DCM (6.0 mL/mmol), at r.t. and under nitrogen atmosphere. The reaction was monitored by TLC following the disappearance of starting materials. After 1.5 h, the reaction was complete; the crude mixture was then transferred into a separatory funnel and washed with HCl (0.1 N in uH_2O) followed by a saturated aqueous solution of NH_4Cl. The collected organic layer was dried over $MgSO_4$, filtered, and concentrated under vacuum. The crude was purified by flash chromatography (eluent: EtOAc/DCM 3:1 v/v → EtOAc/DCM/MeOH 3:1:0.15 v/v/v), affording pure compound **6** in 49% (conversion) yield. ^{1}H-NMR (400 MHz, acetone-d$_6$; Supplementary Figure 11): δ 8.66 (d, *J* = 9.0 Hz, 2H), 8.18 – 8.10 (m, 4H), 7.93 – 7.86 (m, 4H), 7.75 – 7.55 (m, 12H), 7.55 – 7.37 (m, 10H), 7.42 – 7.29 (m, 2H), 7.33 – 7.20 (m, 4H), 6.73 (d, *J* = 8.2 Hz, 1H), 6.42 (s, 2H), 6.16 (td, *J* = 9.3, 1.8 Hz, 2H), 5.98 (dd, *J* = 9.0, 6.0 Hz, 2H), 5.67 (d, *J* = 7.2 Hz, 2H), 5.54 (d, *J* = 6.1 Hz, 2H), 4.96 (dd, *J* = 9.7, 2.1 Hz, 2H), 4.53 (s, 1H), 4.48 – 4.30 (m, 6H), 4.21 – 4.11 (m, 4H), 3.94 (s, 2H), 3.83 (d, *J* = 7.1 Hz, 2H), 3.68 – 3.42 (m, 17H), 3.34 (q, *J* = 5.5 Hz, 4H), 2.68 (t, *J* = 6.7 Hz, 2H), 2.59 (t, *J* = 6.7 Hz, 2H), 2.46 (s, 6H), 2.30 (s, 4H), 2.15 (s, 6H), 1.96 (d, *J* = 2.5 Hz, 12H), 1.83 – 1.72 (m, 2H), 1.65 (s, 6H), 1.20 – 1.15 (m, 12H). ^{13}C-NMR (101 MHz, acetone-*d*$_6$; Supplementary Figure 12): δ 179.00, 174.18, 170.02, 169.81, 166.97, 165.97, 165.69, 160.74, 154.07, 151.28, 146.39, 142.68, 141.17, 137.40, 134.45, 134.42, 133.56, 133.54, 133.21, 131.44, 130.36, 130.12, 129.99, 129.24, 128.82, 128.53, 128.35, 128.31, 128.25, 128.18, 127.64, 127.61, 127.54, 127.52, 127.43, 127.37, 93.69, 84.12, 80.81, 77.80, 77.60, 75.81, 75.30, 75.26, 75.00, 74.97, 71.80, 71.75, 71.52, 70.01, 69.99, 69.80, 69.51, 67.48, 67.18, 66.31, 58.10, 53.65, 46.24, 43.31, 36.25, 22.29, 21.54, 19.95, 14.11, 9.30.

1.10 pMAC2

Compound **6** (1 equiv.) was dissolved in a glass vial with ACN (66 mL/mmol) and cooled at 0 °C, then hydrochloric acid (0.3 M, 3 equiv.) was added dropwise followed by cold $NaNO_2$ dissolved in uH_2O (8.8 mL/mmol). After stirring for 30 min, the solution was added dropwise to another glass vial containing 1-amino-8-naphthol-2,4-disulfonic acid monosodium salt (1.1 equiv.) and $NaHCO_3$ (4 equiv.) in uH_2O (3.3 mL/mmol) at 0 °C; reaction completion (3 h) was followed by TLC. After the disappearance of the starting material **6**, solvents were removed under vacuum; the residue was dissolved in uH_2O, transferred into a cellulose dialysis bag (MWCO: 14 kDa) and dialyzed against excess uH_2O (100:1 v/v) for 24 h, replacing the dialysate every 3 h with fresh uH_2O. The remaining solution was freeze-dried, and the resulting powder was additionally washed with a Et_2O/DCM mixture (9:2 v/v) to remove possible impurities and afford pure pMAC2 in quantitative yield. ^{1}H-NMR (500

MHz, CD_3OD; Supplementary Figure 13): δ δ 8.72 (s, 1H), 8.13 – 8.08 (m, 4H), 7.99 (d, *J* = 9.8 Hz, 1H), 7.90 (d, *J* = 8.5 Hz, 1H), 7.82 – 7.74 (m, 4H), 7.66 (t, *J* = 7.4 Hz, 2H), 7.57 (t, *J* = 7.7 Hz, 6H), 7.54 – 7.36 (m, 19H), 7.25 (d, *J* = 7.4 Hz, 2H), 7.13 (d, *J* = 9.9 Hz, 1H), 6.43 (s, 2H), 6.07 (t, *J* = 9.1 Hz, 2H), 5.82 (d, *J* = 6.6 Hz, 2H), 5.62 (d, *J* = 7.1 Hz, 2H), 5.46 (d, *J* = 6.5 Hz, 2H), 4.60 (s, 2H), 4.41 (dt, *J* = 11.7, 5.9 Hz, 2H), 4.32 (dt, *J* = 11.8, 5.8 Hz, 4H), 4.17 (s, 4H), 3.79 (d, *J* = 7.1 Hz, 2H), 3.67 (dd, *J* = 11.0, 6.0 Hz, 2H), 3.55 (dtd, *J* = 22.1, 11.5, 10.0, 5.3 Hz, 20H), 3.37 (d, *J* = 5.4 Hz, 2H), 2.74 (t, *J* = 7.0 Hz, 2H), 2.61 (q, *J* = 7.1, 5.9 Hz, 2H), 2.50 (s, 3H), 2.38 (s, 4H), 2.29 (s, 6H), 2.21 – 2.11 (m, 8H), 1.92 – 1.89 (m, 4H), 1.81 (dd, *J* = 15.0, 8.8 Hz, 2H), 1.64 (s, 6H), 1.29 (s, 8H), 1.12 (d, *J* = 6.3 Hz, 12H). MS (ES+): m/z calcd for $C_{138}H_{146}N_8O_{45}S_4+H^+$ 2763.83 $[M+H]^+$; found 2763.77 (Supplementary Figure 14).

1.11 Compound 7

Nlg (1 equiv.) was placed in a two-necked round-bottomed flask under nitrogen atmosphere and dissolved with dry dichloromethane (6 mL/mmol). 4-dimethylaminopyridine (DMAP; 3.5 equiv.) was added to the solution cooled at 0 °C. A solution of triphosgene (0.4 equiv.) dissolved in DCM (7.1 mL/mmol) was then added dropwise; the reaction mixture was stirred for 2 h and 2,2′-disulfanediylbis(ethan-1-ol) was added (2.3 equiv.). After stirring for 24 h at 0 °C, the reaction mixture was transferred into a separatory funnel extracted with DCM and washed with HCl (0.1 N in uH_2O) and uH_2O. The collected organic layer was dried over $MgSO_4$, filtered and concentrated under vacuum. The crude was purified by flash chromatography (eluent: DCM/cHex/MeOH 10:1:1 v/v/v) affording pure compound **7** in 52% yield. ^{1}H-NMR (400 MHz, $CDCl_3$; Supplementary Figure 15): δ 7.79 (s, 1H), 7.52 (dt, *J* = 7.6, 0.9 Hz, 1H), 7.46 (dq, *J* = 7.6, 0.9 Hz, 1H), 7.36 (tt, *J* = 7.5, 0.8 Hz, 1H), 7.25 (td, *J* = 7.6, 1.2 Hz, 1H), 7.15 (s, 1H), 5.26 – 5.13 (m, 1H), 4.88 – 4.75 (m, 1H), 4.44 – 4.27 (m, 2H), 3.86 (tt, *J* = 7.5, 3.9 Hz, 2H), 2.91 (dt, *J* = 19.2, 6.3 Hz, 6H), 2.36 (m, 1H), 2.23 (m, 1H), 1.74 (m, 2H), 1.73 – 1.61 (m, 1H), 1.56 (ddt, *J* = 11.7, 6.0, 3.2 Hz, 1H), 1.27 – 0.96 (m, 6H). ^{13}C-NMR (101 MHz, $CDCl_3$; Supplementary Figure 16): δ 154.81, 144.40, 137.64, 132.17, 130.10, 128.75, 126.78, 123.93, 120.26, 118.47, 58.35, 42.28, 41.93, 37.22, 28.33, 28.03, 26.26, 25.97.

1.12 Compound 8

Compound **7** (1 equiv.) was placed in a two-necked round-bottomed flask under nitrogen atmosphere and dissolved with dry DCM (47 mL/mmol). DMAP (1 equiv.) was added to the solution followed by triethylamine (TEA; 4 equiv.) and p-nitrophenyl chloroformate (2 equiv.). The reaction was stirred at r.t. for 4 h and monitored by TLC. Once complete, the solution was transferred to a separatory funnel extracted with DCM and washed with HCl (0.1 N in uH_2O) and brine. The collected organic layer was dried over $MgSO_4$, filtered and concentrated under vacuum. The crude was purified by flash chromatography (eluent: DCM/cHex/MeOH 10:4:0.25 v/v/v), affording pure compound **8** in 60% yield. ^{1}H-NMR (400 MHz, $CDCl_3$; Supplementary Figure 17): δ 8.31 – 8.23 (m, 2H), 7.73 (s, 1H), 7.57 – 7.47 (m, 2H), 7.41 – 7.32 (m, 3H), 7.25 (td, *J* = 7.6, 1.2 Hz, 1H), 7.19 (s, 1H), 5.18 (dd, *J* = 7.2, 4.3 Hz, 1H), 4.80 (ddd, *J* = 10.3, 5.4, 2.5 Hz, 1H), 4.53 (t, *J* = 6.5 Hz, 2H), 4.41 – 4.29 (m, 1H), 4.33 – 4.23 (m, 1H), 3.03 (t, *J* = 6.5 Hz, 2H), 2.94 (t, *J* = 6.6 Hz, 2H), 2.43 (m, 1H), 2.19 (m, 1H), 1.78 – 1.69 (m, 2H), 1.69 – 1.62 (m, 3H), 1.60 – 1.47 (m, 1H), 1.28 – 0.92 (m, 5H).

1.13 Compound 9

Compound **5** (1.5 equiv.) was placed in a two-necked round-bottomed flask under nitrogen atmosphere and dissolved with dry DCM (2.5 mL/mmol). A solution of compound **8** (1 equiv.) dissolved in DCM (1 mL/mmol) was then added followed by DIPEA (8 equiv.). The reaction was stirred at r.t. for 2 h and monitored by TLC. Once complete, the solution was transferred to a separatory funnel extracted with DCM and washed with HCl (0.1 N in uH_2O) and a saturated aqueous solution of NH_4Cl. The collected organic layer was dried over $MgSO_4$, filtered and concentrated under vacuum. The crude was purified

by flash chromatography (eluent: DCM/cHex/MeOH 10:2:1 v/v/v) affording pure compound **9** in 32% yield. ^{1}H-NMR (400 MHz, $CDCl_3$; Supplementary Figure 18): δ 88.15 (s, 1H), 7.82 (d, *J* = 8.3 Hz, 1H), 7.75 (s, 1H), 7.52 (dd, *J* = 10.7, 7.6 Hz, 2H), 7.41 – 7.31 (m, 3H), 7.30 – 7.21 (m, 2H), 7.18 (s, 1H), 6.71 (d, *J* = 8.0 Hz, 1H), 5.51 (s, 1H), 5.18 (dd, *J* = 7.3, 4.2 Hz, 1H), 4.79 (m, 1H), 4.28 (q, *J* = 6.6 Hz, 4H), 3.60 – 3.51 (m, 8H), 3.51 – 3.43 (m, 2H), 3.35 (d, *J* = 5.8 Hz, 2H), 3.16 (td, *J* = 6.6, 3.4 Hz, 1H), 2.94 – 2.85 (m, 4H), 2.79 – 2.71 (m, 2H), 2.66 (d, *J* = 6.7 Hz, 2H), 2.42 (m, 1H), 2.30 (s, 3H), 2.22 (s, 3H), 2.21 – 2.12 (m, 1H), 1.85 – 1.77 (m, 1H), 1.73 (s, 2H), 1.65 (d, *J* = 10.2 Hz, 3H), 1.23 – 1.03 (m, 3H), 1.05 – 0.95 (m, 2H). ^{13}C-NMR (126 MHz, $CDCl_3$; Supplementary Figure 19): δ 154.65, 144.11, 137.48, 131.59, 128.96, 128.61, 128.36, 126.65, 125.42, 124.48, 123.99, 123.22 (d, J = 3.0 Hz), 122.54, 120.21, 104.17, 102.52, 78.67, 70.29, 70.09 (d, J = 22.5 Hz), 69.74, 63.78, 42.15, 40.80, 39.42, 37.66, 36.88, 31.90, 29.68, 28.31, 27.76, 26.10 (d, J = 2.9 Hz), 25.82, 17.97, 17.52, 15.36.

1.14 nMAC

Compound **9** (1 equiv.) was dissolved in a glass vial with ACN (0.028 mL/mmol **11**) and cooled at 0 °C; then, hydrochloric acid (0.3 M, 3 equiv.) was added dropwise followed by cold $NaNO_2$ dissolved in uH_2O (6.1 mL/mmol $NaNO_2$). After stirring for 30 min, the solution was added dropwise to another glass vial containing 1-amino-8-naphthol-2,4-disulfonic acid monosodium salt (1.1 equiv.) and $NaHCO_3$ (4 equiv.) dissolved in uH_2O (3.3 mL/mmol $NaHCO_3$) and kept at 0 °C; reaction completion (3 h) was followed by TLC. After the disappearance of the starting compound **9**, ACN was removed under vacuum; the residual aqueous solution was transferred into a cellulose dialysis bag (MWCO: 14 kDa) and dialyzed against excess uH_2O (100:1 v/v) for 24 h, with the dialysate replaced with fresh uH_2O every 3 h. The remaining solution was freeze-dried washed with a Et_2O/DCM mixture (2:1 v/v) to obtain pure nMAC as a dark purple powder in 70% yield. ^{1}H-NMR (400 MHz, DMSO-d_6; Supplementary Figure 20): δ 9.33 (s, 1H), 8.36 (s, 1H), 8.04 (d, *J* = 10.0 Hz, 1H), 7.97 – 7.86 (m, 2H), 7.64 (dd, *J* = 4.6, 2.6 Hz, 2H), 7.58 – 7.46 (m, 4H), 7.37 (dt, *J* = 22.4, 8.0 Hz, 2H), 7.31 – 7.22 (m, 1H), 7.22 (d, *J* = 5.6 Hz, 1H), 7.11 (s, 1H), 6.99 (d, *J* = 9.9 Hz, 0H), 5.40 (t, *J* = 5.0 Hz, 1H), 4.30 (dt, *J* = 8.6, 4.3 Hz, 1H), 4.16 (t, *J* = 6.4 Hz, 2H), 4.07 (dt, *J* = 11.2, 6.4 Hz, 1H), 3.96 (dt, *J* = 11.6, 6.2 Hz, 1H), 3.50 (d, *J* = 2.1 Hz, 6H), 3.44 – 3.37 (m, 4H), 3.22 (s, 1H), 3.12 (d, *J* = 5.8 Hz, 3H), 2.89 (dt, *J* = 23.3, 6.3 Hz, 4H), 2.59 (d, *J* = 6.5 Hz, 2H), 2.44 (t, *J* = 6.6 Hz, 1H), 2.28 (s, 2H), 2.32 – 2.19 (m, 1H), 2.10 (d, *J* = 12.5 Hz, 2H), 1.64 (s, 2H), 1.56 (s, 2H), 1.52 (d, *J* = 12.6 Hz, 2H), 1.20 – 1.04 (m, 6H), 0.99 – 0.81 (m, 3H). MS (ES+): m/z calcd for $C_{58}H_{68}N_8O_{16}S_4+H^+$ 1261.37 $[M+H]^+$; found 1261.00; MS (ES-): m/z calcd for $C_{58}H_{68}N_8O_{16}S_4-H^-$ 1259.36 $[M-H]^-$; found 1258.97 (Supplementary Figure 21).

1.15 HPLC-MS analyses

HPLC-MS analyses were performed on a Dyonex Ultimate 3000 HPLC (Thermo Fisher Scientific, Italy) equipped with a triple quadrupole mass spectrometer TSQ Quantum Access Max and electrospray ionization source detector. 0.5 mL samples were used as sources for the automated injection. The chromatographic separation was performed on a reverse phase Zorbax C18 column 4.6 x 150 mm, 5 μm (Agilent Technologies, CA, USA), at flow rate of 0.5 mL/min, linear gradient mobile phases A (HCOOH 0.1% in uH_2O v/v) and B MeOH (NH_4OAc 5 mM and HCOOH 0.1% in MeOH v/v) from 30:70 to 5:95. Chromatograms at the relevant detection wavelength 560 nm for pMAC1, pMAC2 and nMAC were extracted from PDA data (600–200 nm).

1.16 Sensorgrams

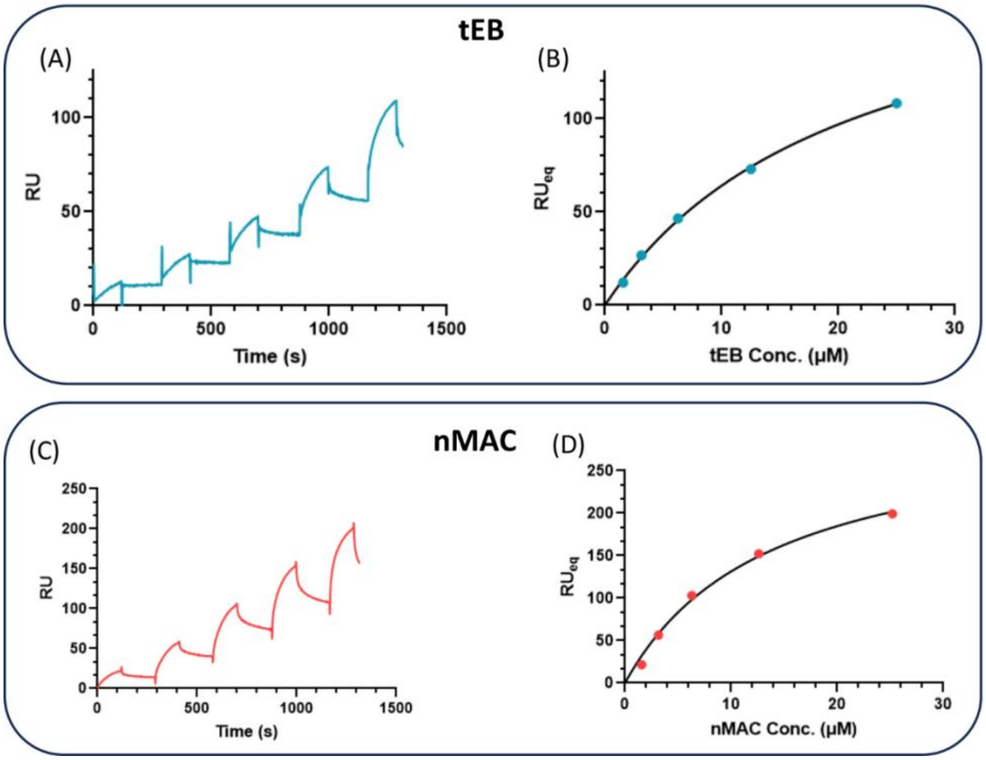

Supplementary Figure 1. SPR analysis. Representative sensorgram profiles and isothermal binding curves for tEB (A, B), and nMAC (C, D). Double-referenced single-cycle sensorgrams are reported.

2 NMR and LC-MS Spectra

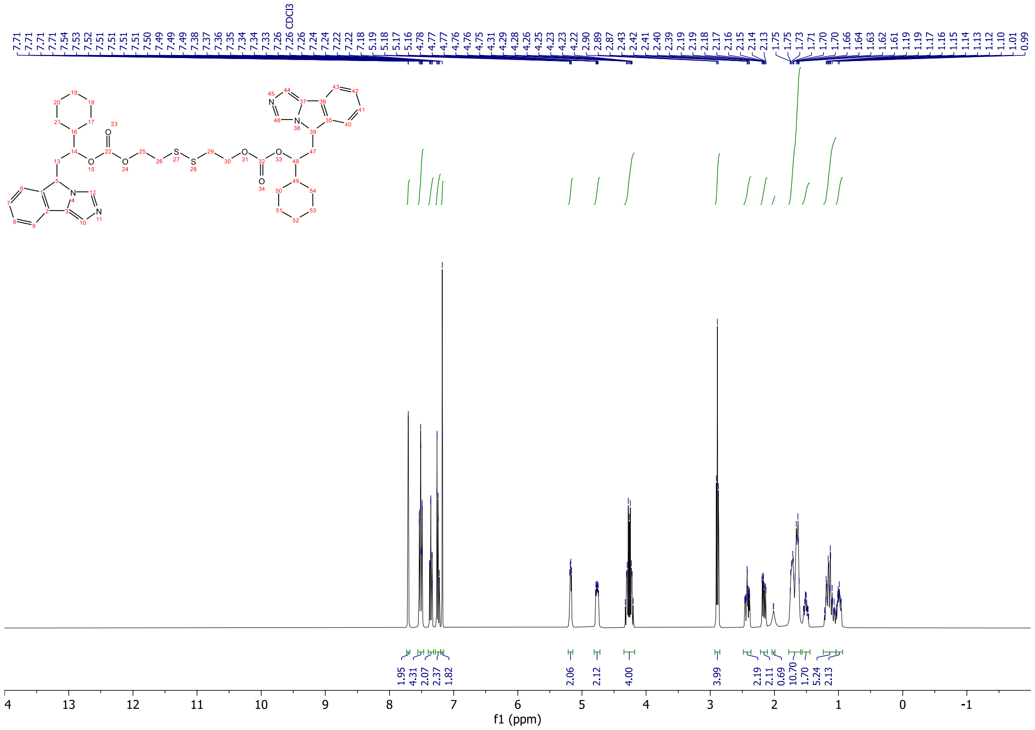

Supplementary Figure 2. ^{1}H-NMR spectrum of NlgD(400 MHz, $CDCl_3$).

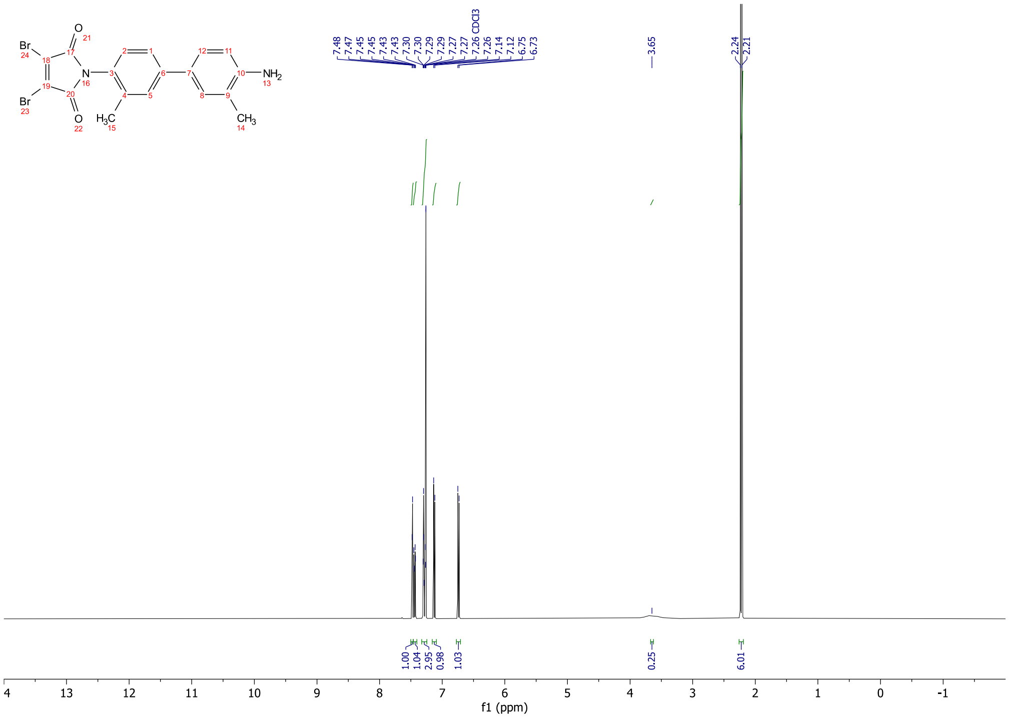

Supplementary Figure 3. ^{1}H-NMR spectrum of compound **2** (400 MHz, $CDCl_3$).

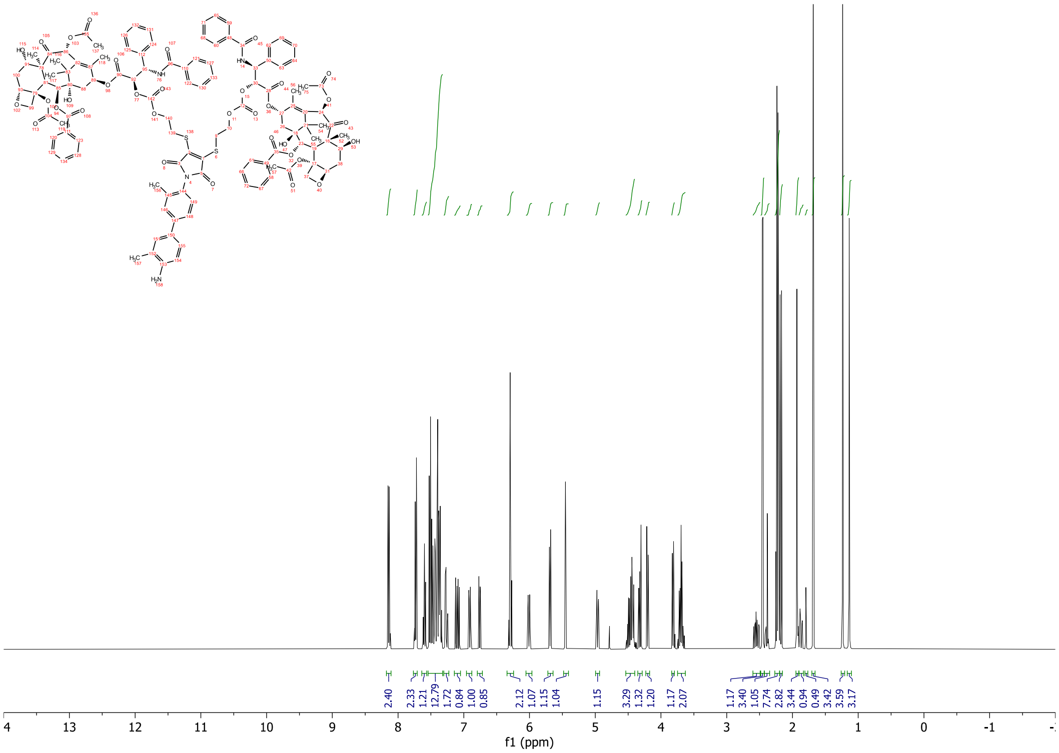

Supplementary Figure 4. ^{1}H-NMR spectrum of compound **3** (400 MHz, $CDCl_3$).

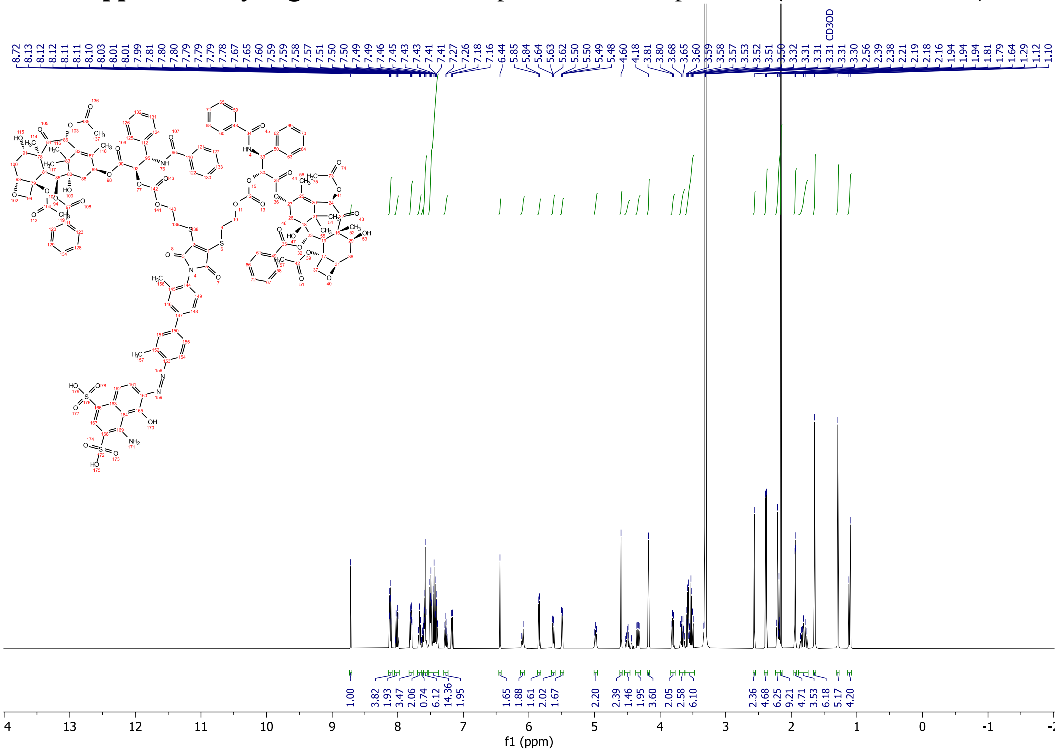

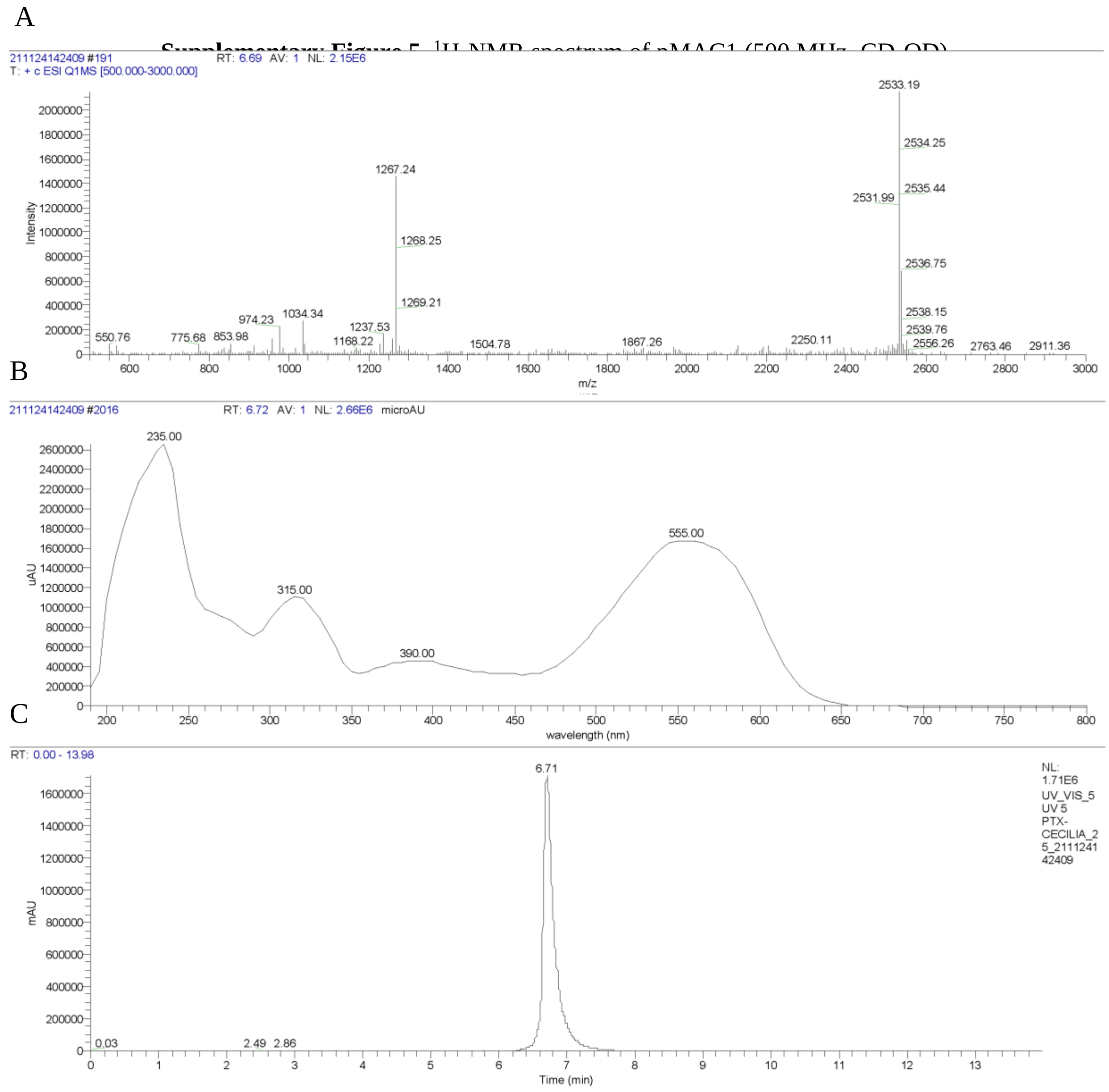

Supplementary Figure 6. LC-MS analysis of pMAC1. (A) Mass spectrum of peak at t_R = 6.71 min; (B) UV spectrum of peak at t_R = 6.71 min; (C) HPLC profile of pMAC1 at (λ = 560nm).

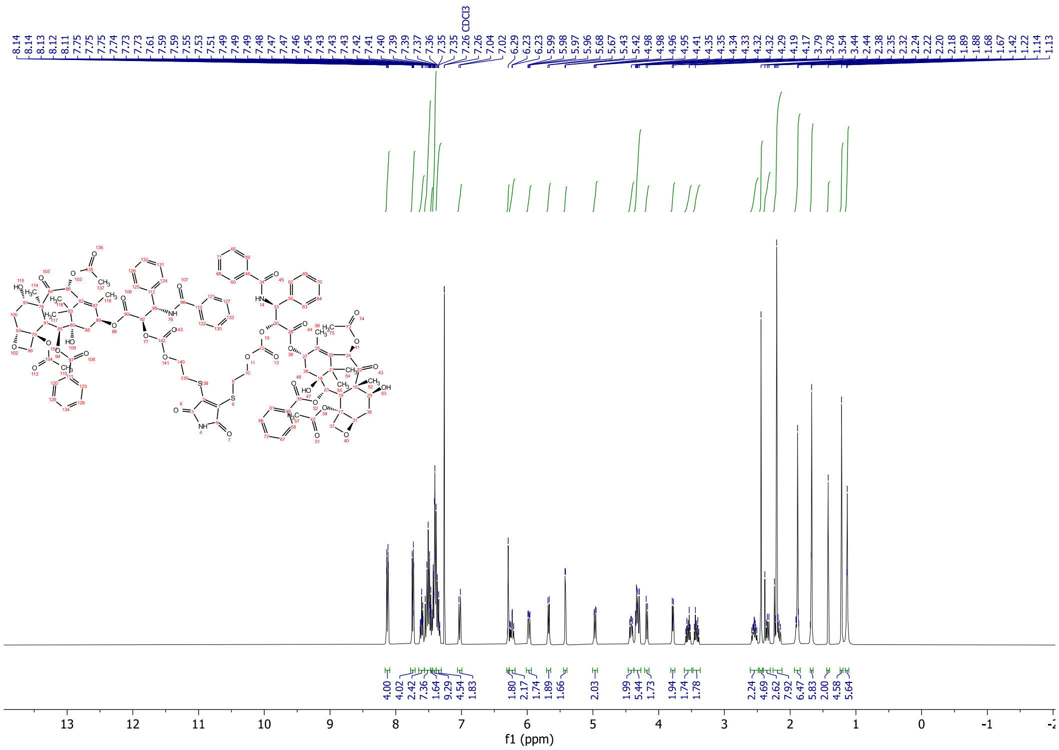

Supplementary Figure 7. ^{1}H-NMR spectrum of pMal (400 MHz, $CDCl_3$).

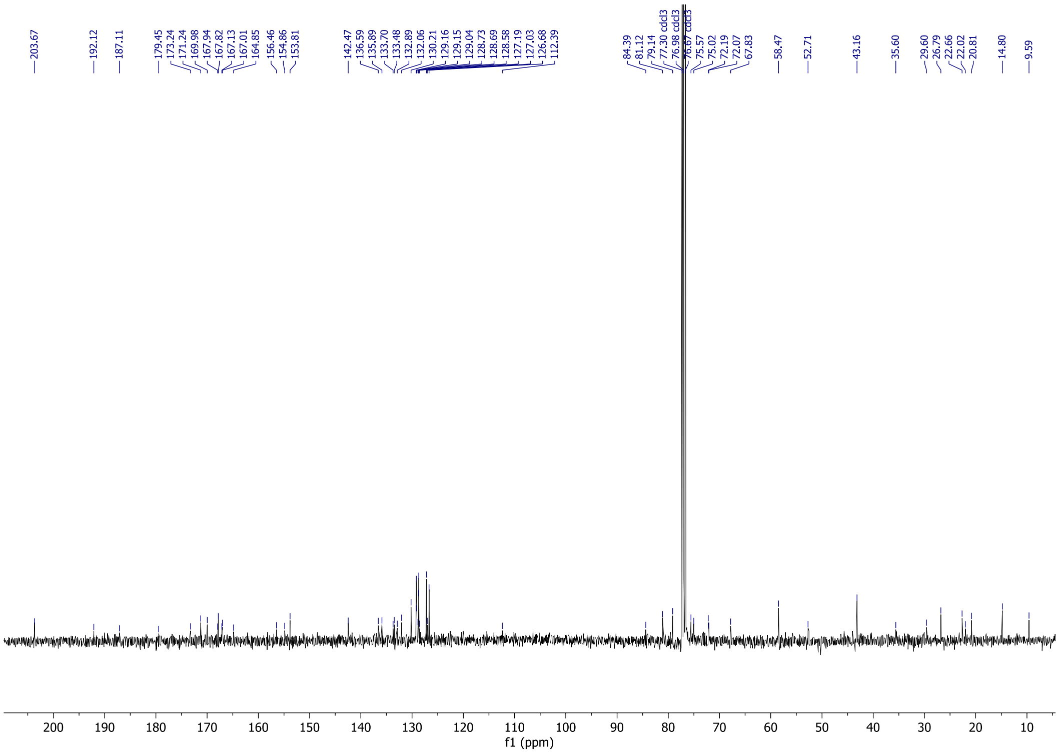

Supplementary Figure 8. ^{13}C-NMR spectrum of pMal(400 MHz, $CDCl_3$).

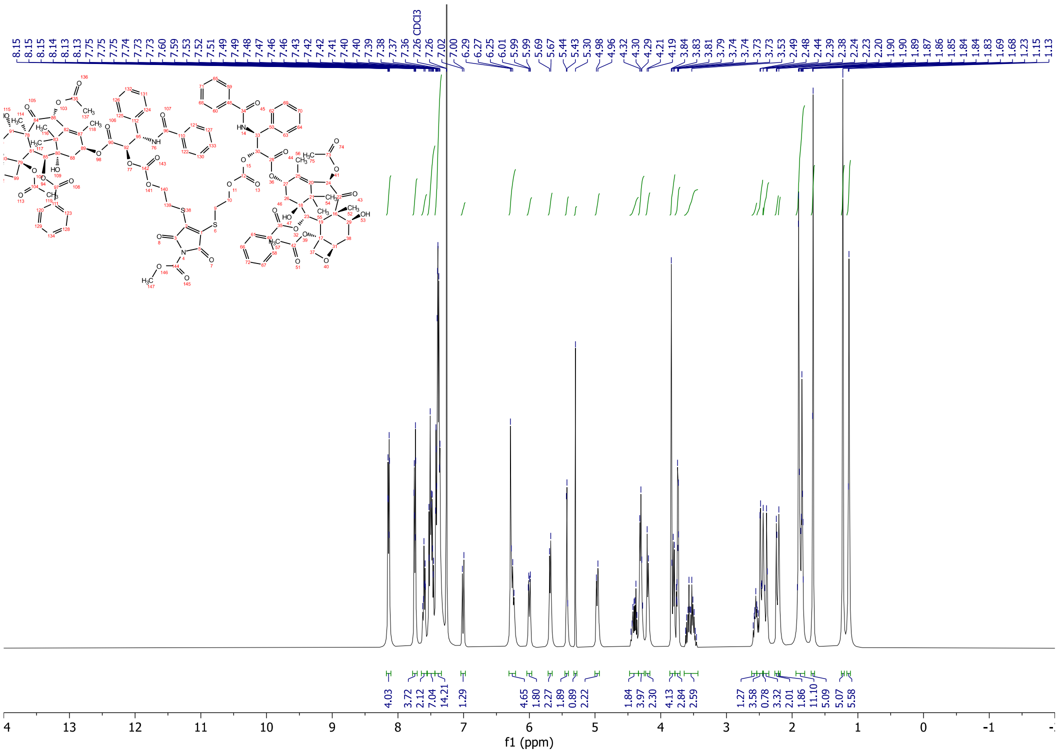

Supplementary Figure 9. ^{1}H-NMR spectrum compound **4** (400 MHz, $CDCl_3$).

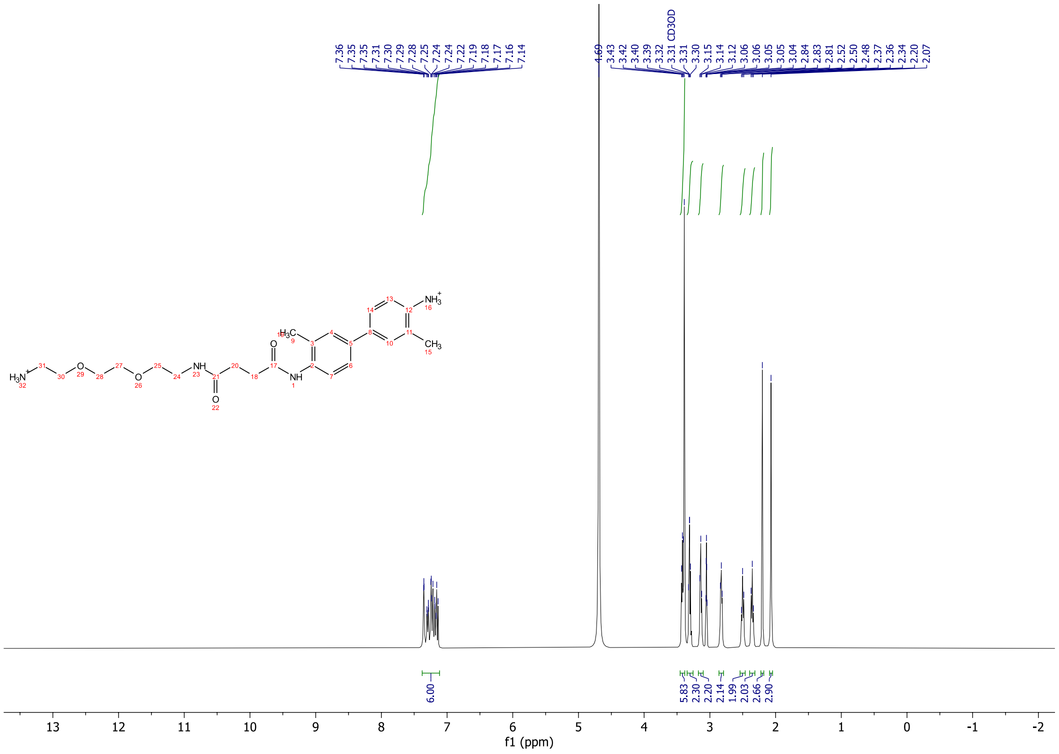

Supplementary Figure 10. ^{1}H-NMR spectrum of compound **5** (400 MHz, CD_3OD).

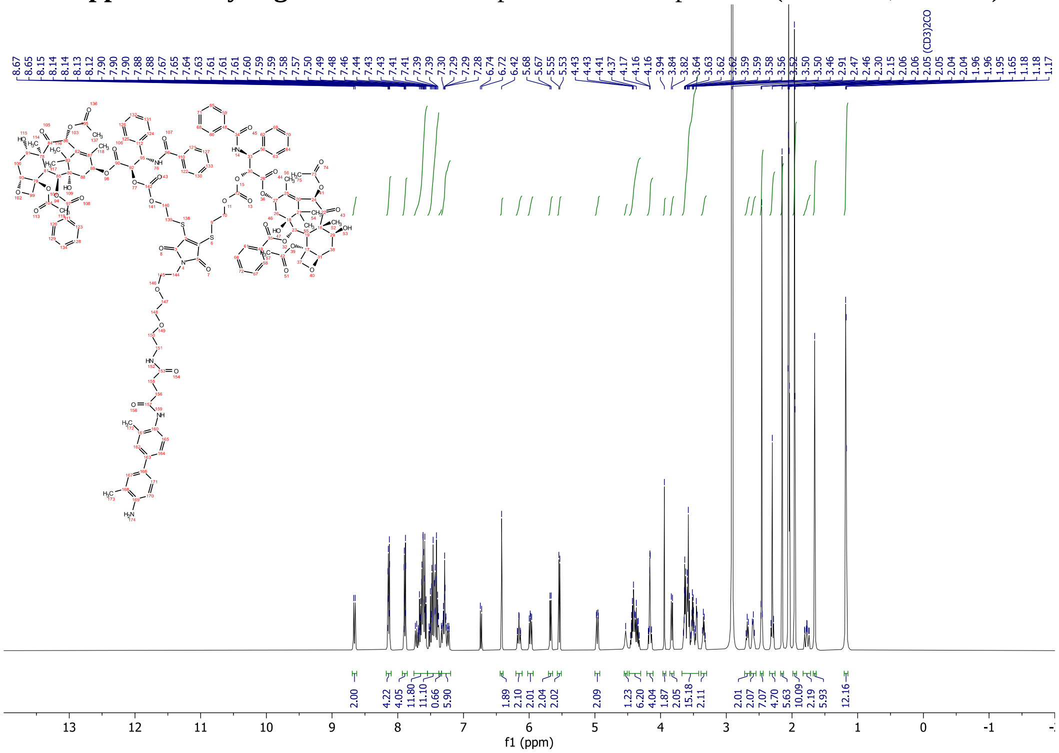

Supplementary Figure 11. ^{1}H-NMR spectrum of compound **6** (400 MHz, acetone-d_6).

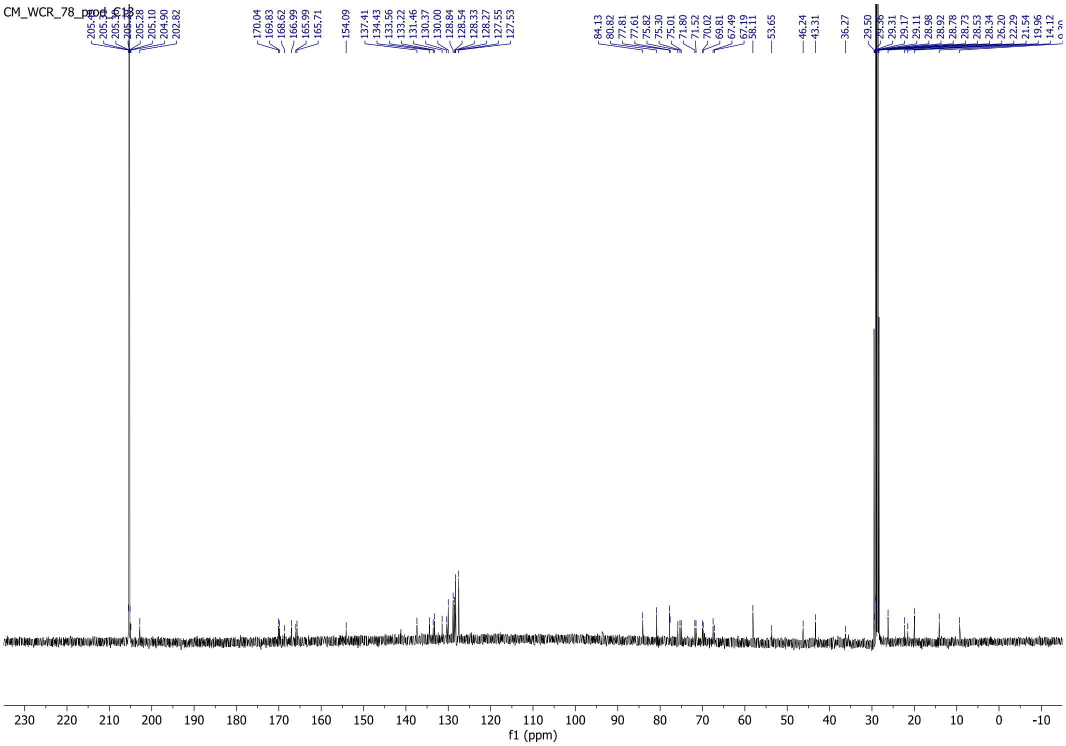

Supplementary Figure 12. ^{13}C-NMR spectrum of compound **6** (101 MHz, acetone-d_6).

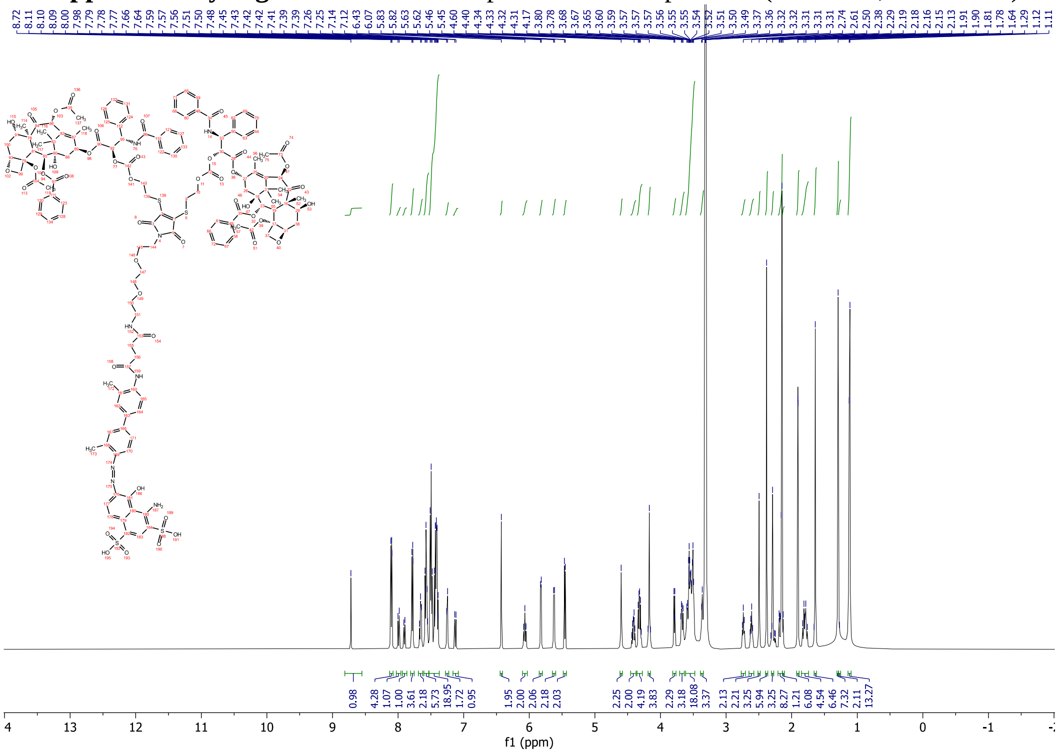

A Supplementary Figure 13. ^1H NMR spectrum of pMAC2(500 MHz, CD_3OD)

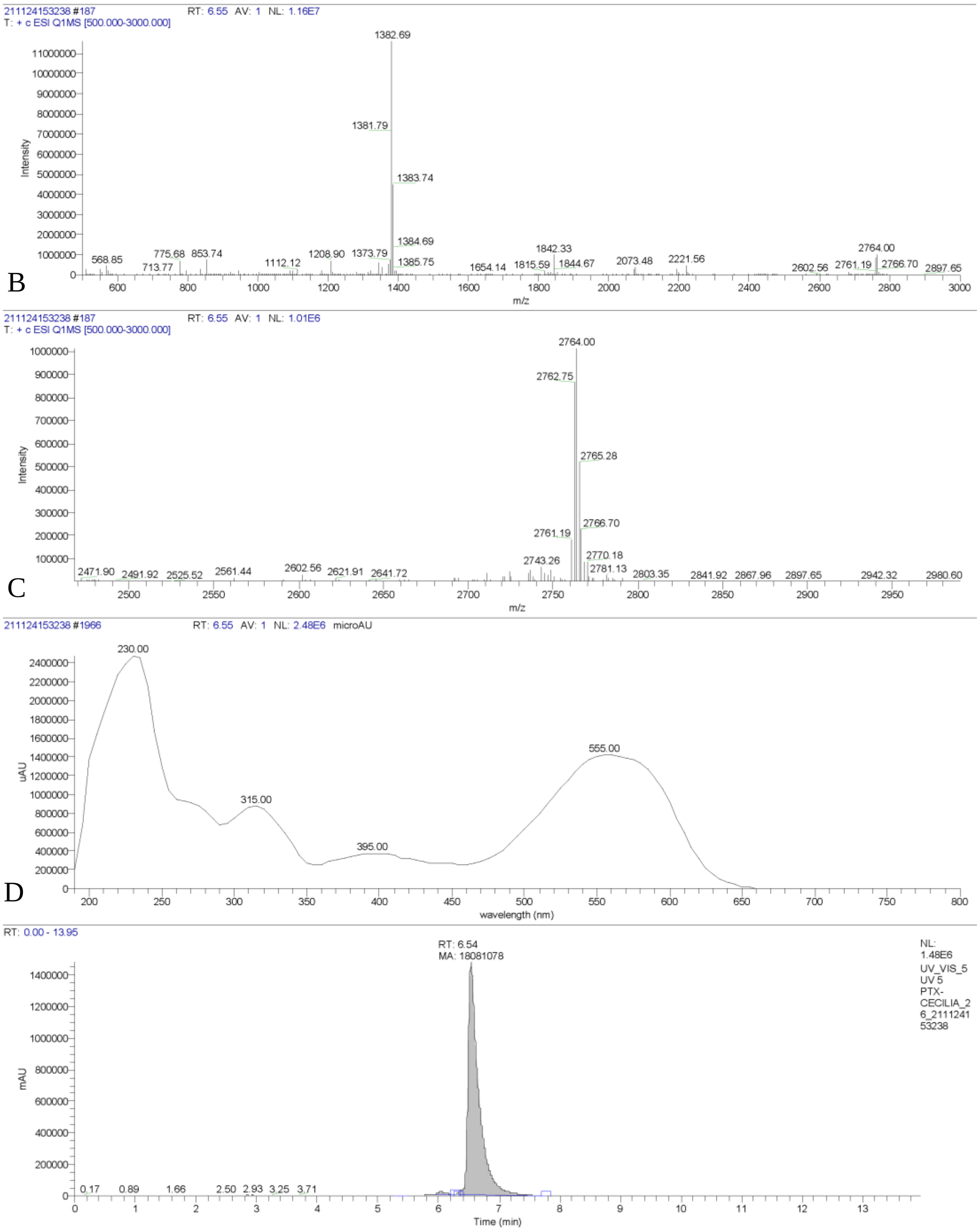

Supplementary Figure 14. LC-MS analysis of pMAC2. (A) Mass spectrum of peak at t_R = 6.54 min; (B) Magnification of mass spectrum; (C) UV spectrum of peak at t_R = 6.54 min; (D) HPLC profile of pMAC2 (λ = 560 nm).

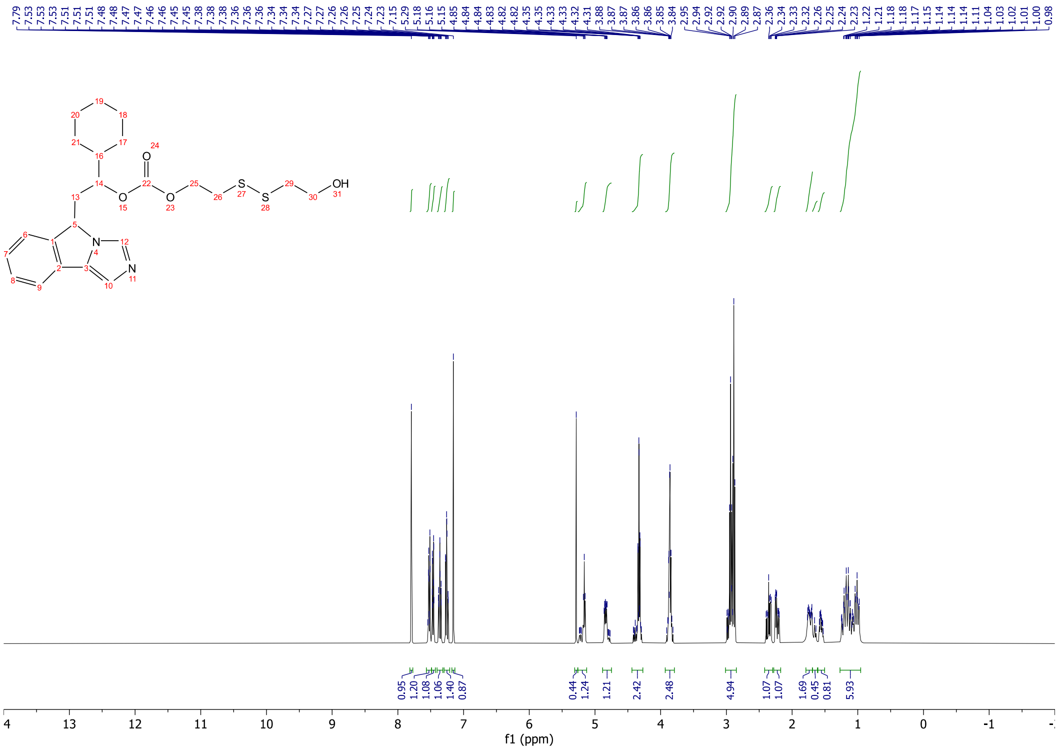

Supplementary Figure 15. ^{1}H-NMR spectrum of compound **7**(400 MHz, $CDCl_3$).

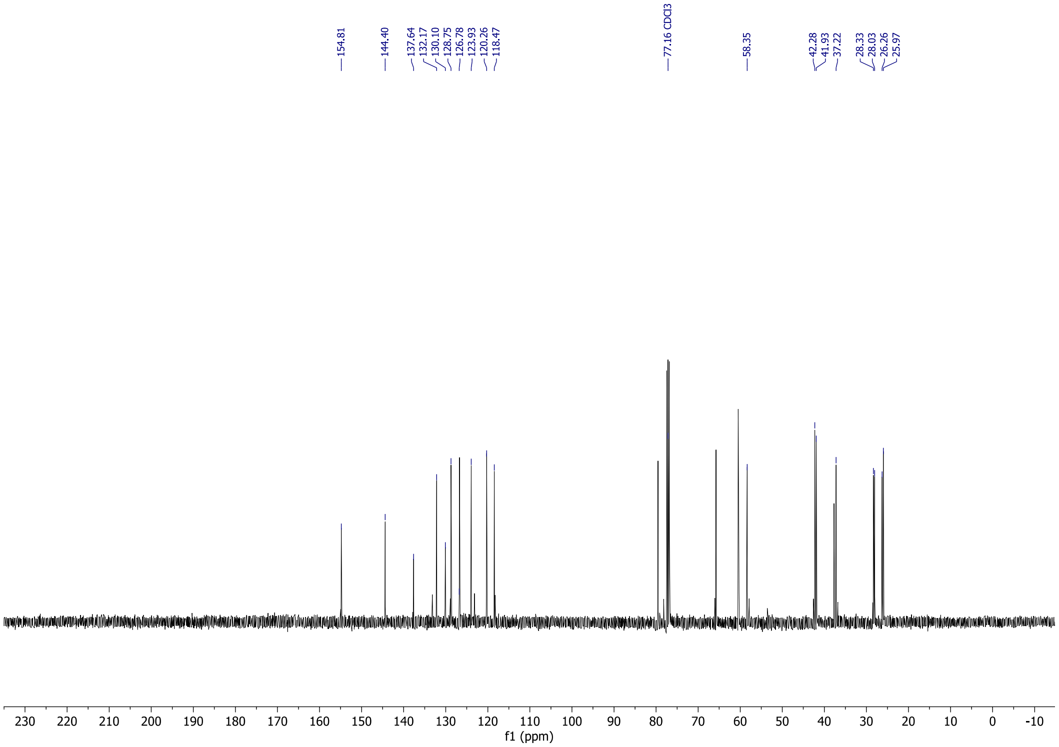

Supplementary Figure 16. ^{13}C-NMR spectrum compound **7** (101 MHz, $CDCl_3$).

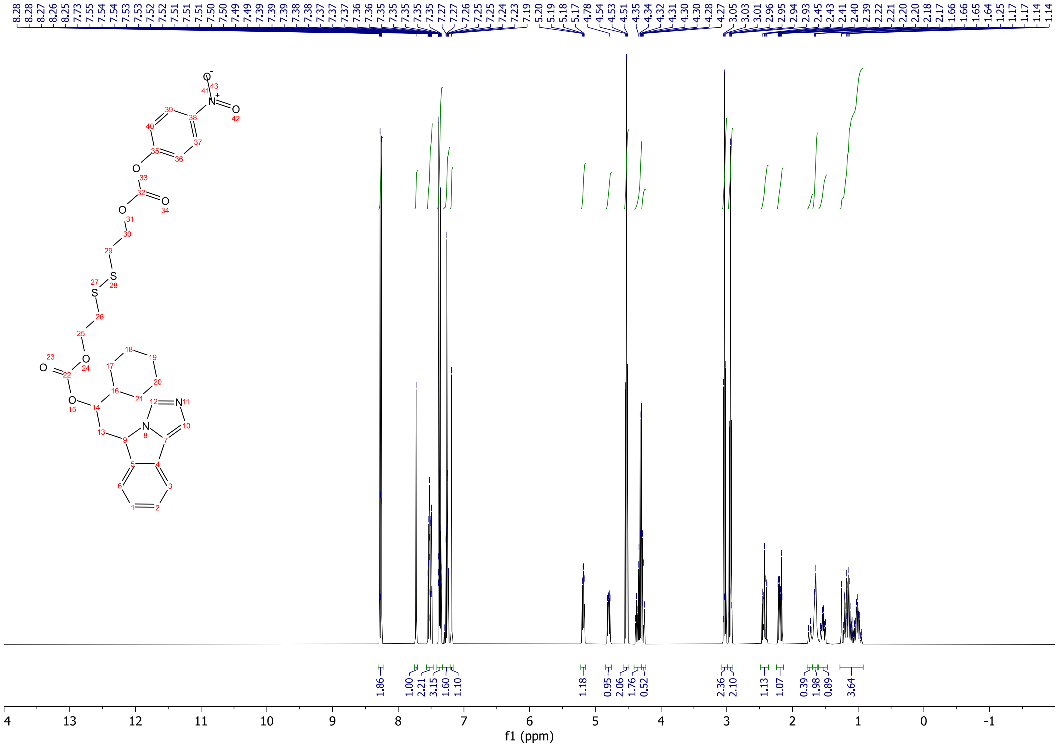

Supplementary Figure 17. ^{1}H-NMR spectrum of compound **8** (400 MHz, $CDCl_3$).

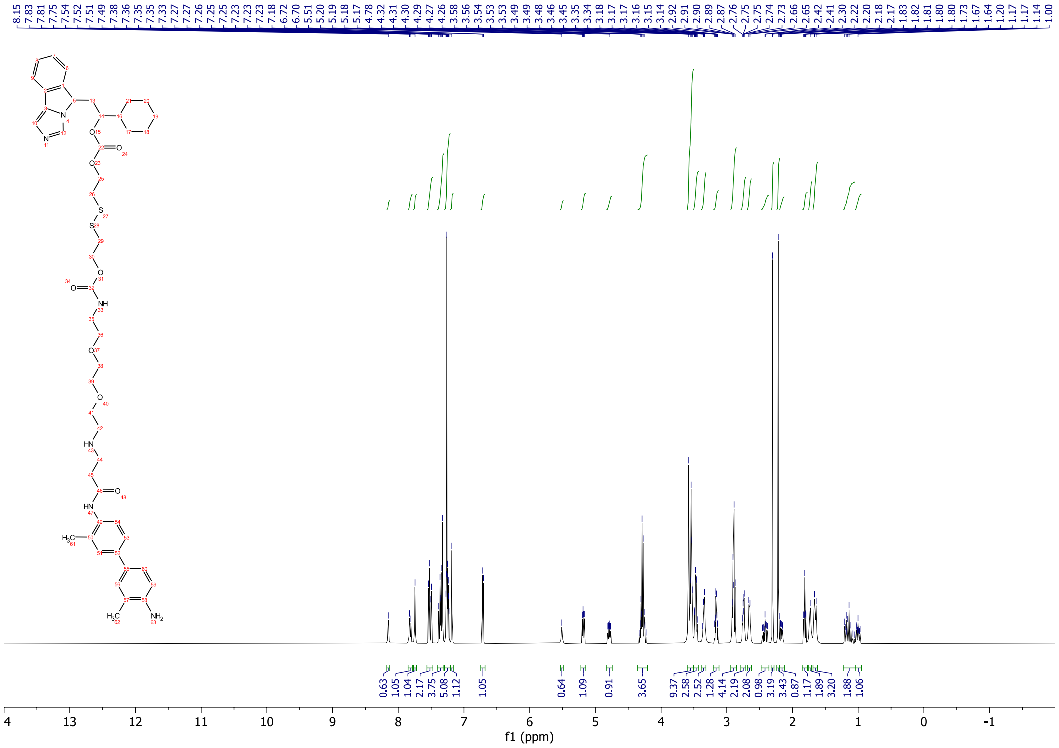

Supplementary Figure 18. ^{1}H-NMR spectrum of compound **9**(400 MHz, $CDCl_3$).

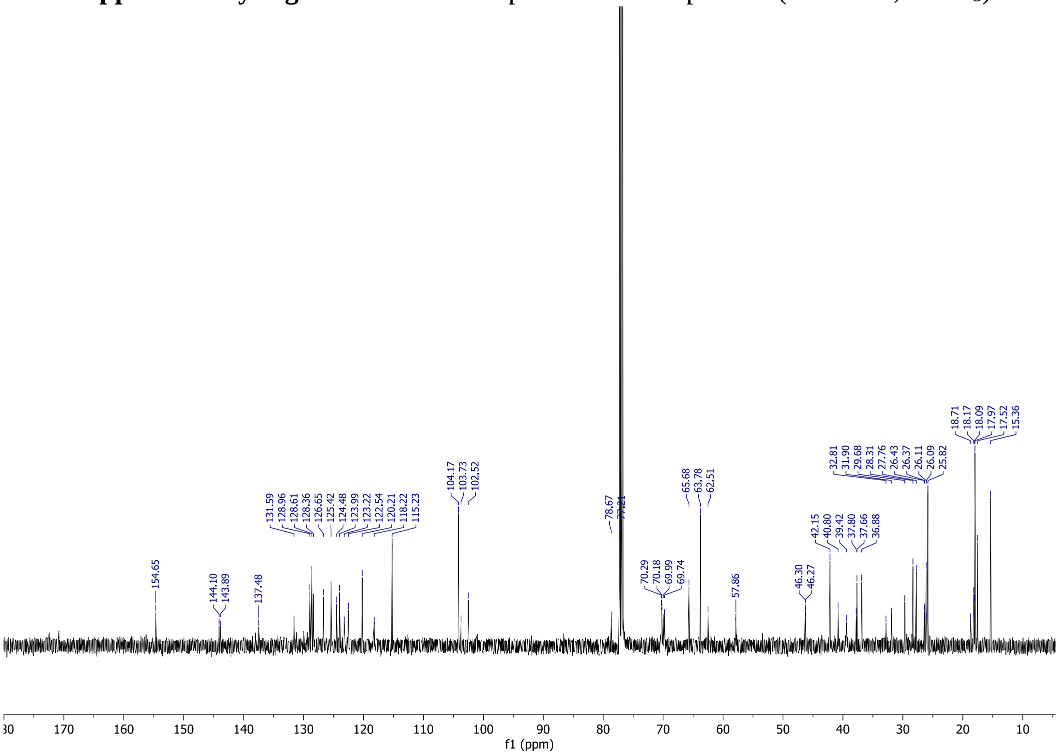

Supplementary Figure 19. ^{13}C-NMR spectrum of compound **9** (126 MHz, $CDCl_3$).

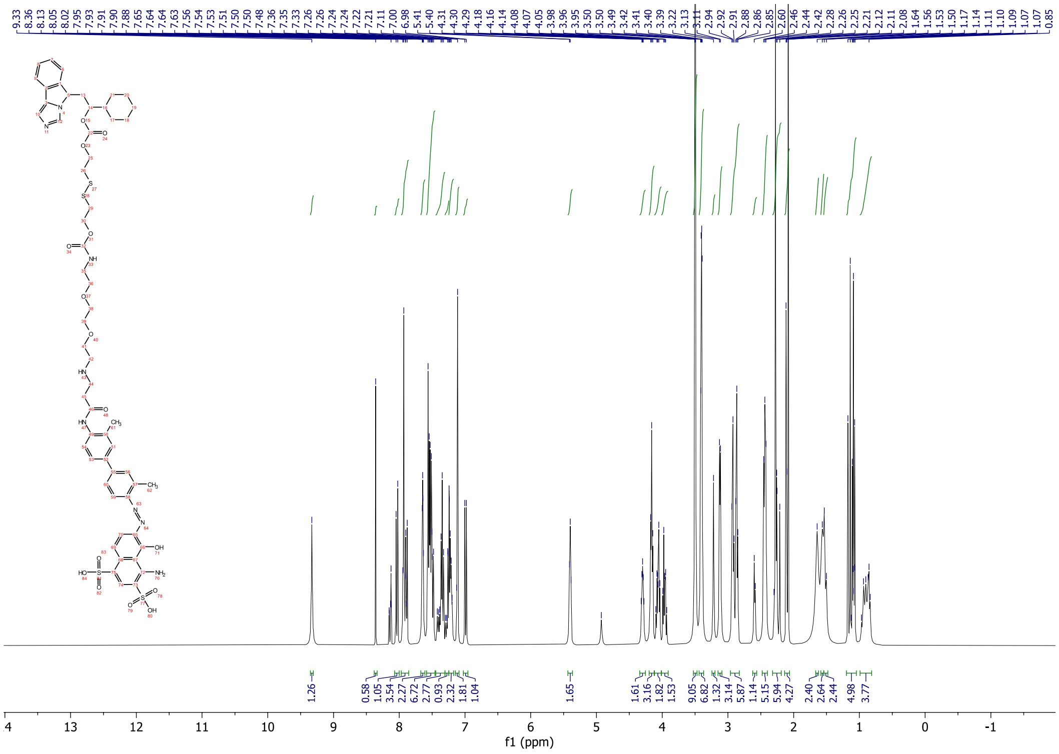

A Supplementary Figure 20. ^{1}H NMR spectrum of compound nMAC(400 MHz, DMSO-d_6)

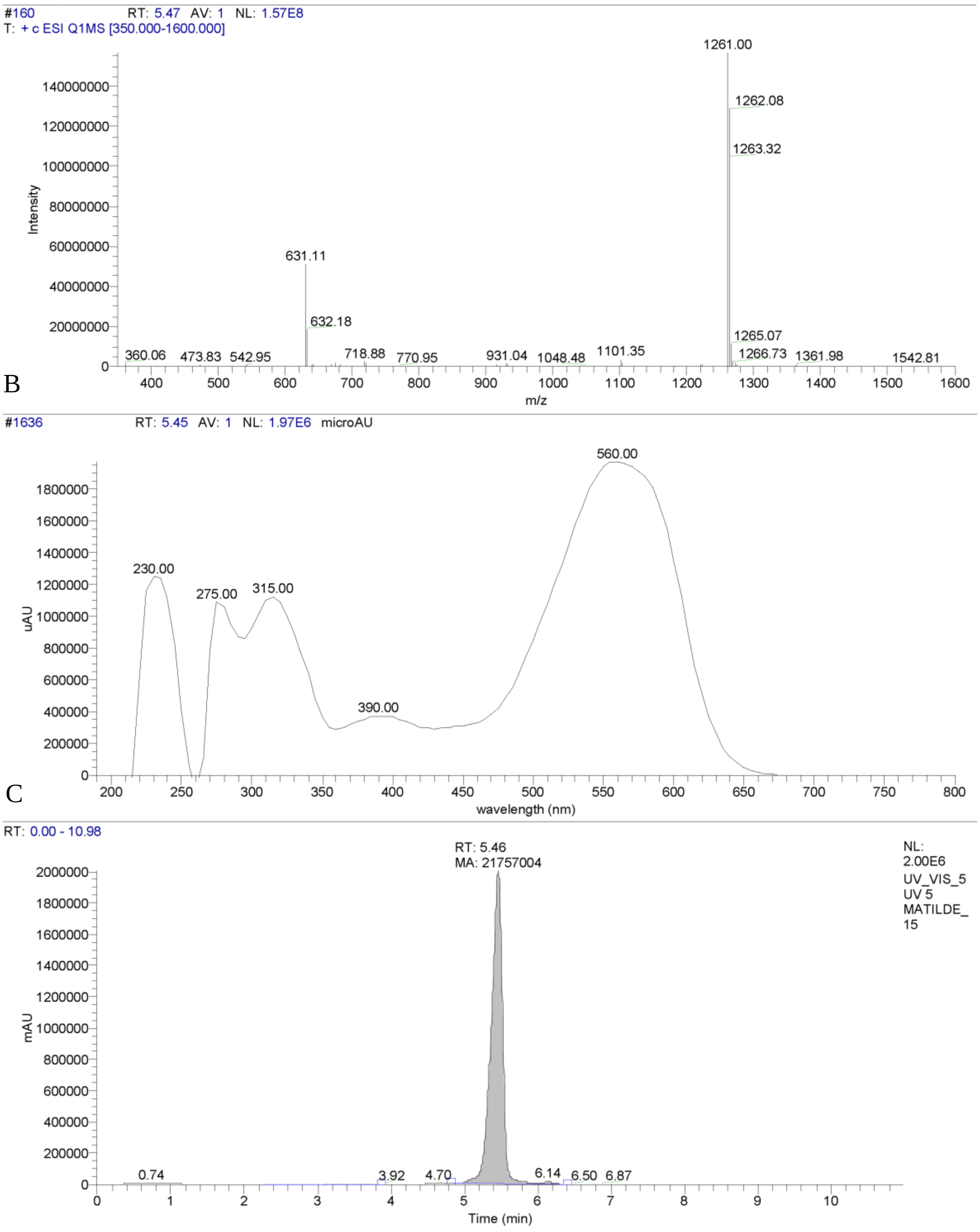

Supplementary Figure 21. LC-MS analysis of nMAC. (A) Mass spectrum of peak at t_R = 5.46 min; (B) UV spectrum of peak at t_R = 5.46 min; (C) HPLC profile of nMAC (λ = 560 nm).

3 HPLC Analysis

3.1 UV spectra of drugs and prodrugs

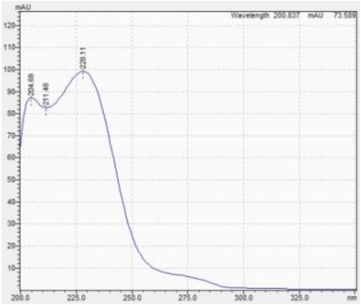

Ptx

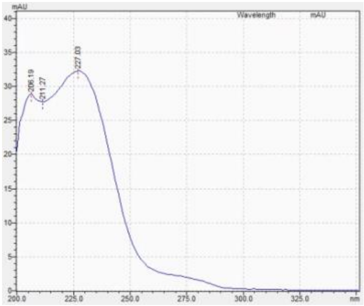

7-epi-Ptx

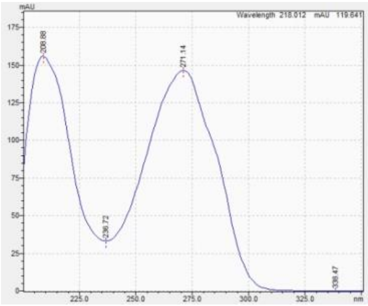

(*S*,*R*)/(*R*,*S*)-Nlg

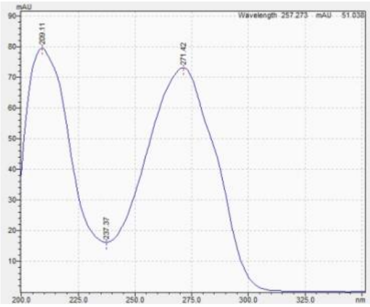

(*R*,*R*)/(*S*,*S*)-Nlg

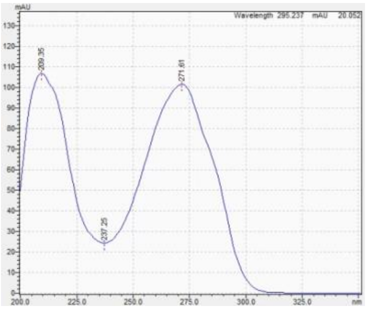

NlgD

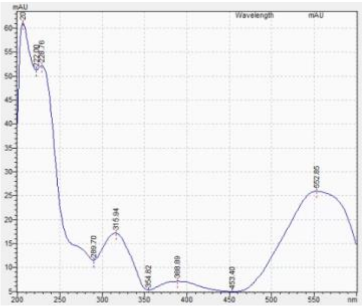

pMAC1

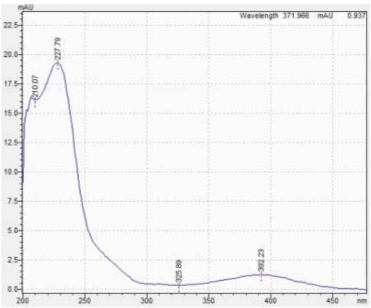

pMal

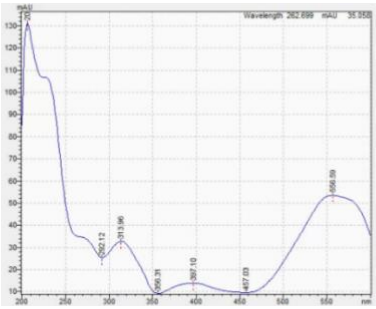

pMAC2

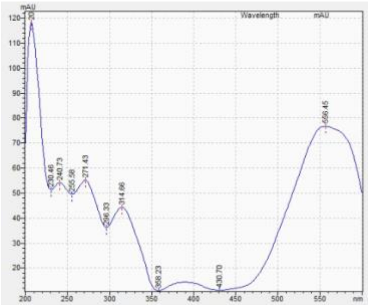

nMAC

Supplementary Figure 22. PDA-extracted UV spectra of Ptx, Nlg and their prodrugs.

3.2 Prodrug stability over time

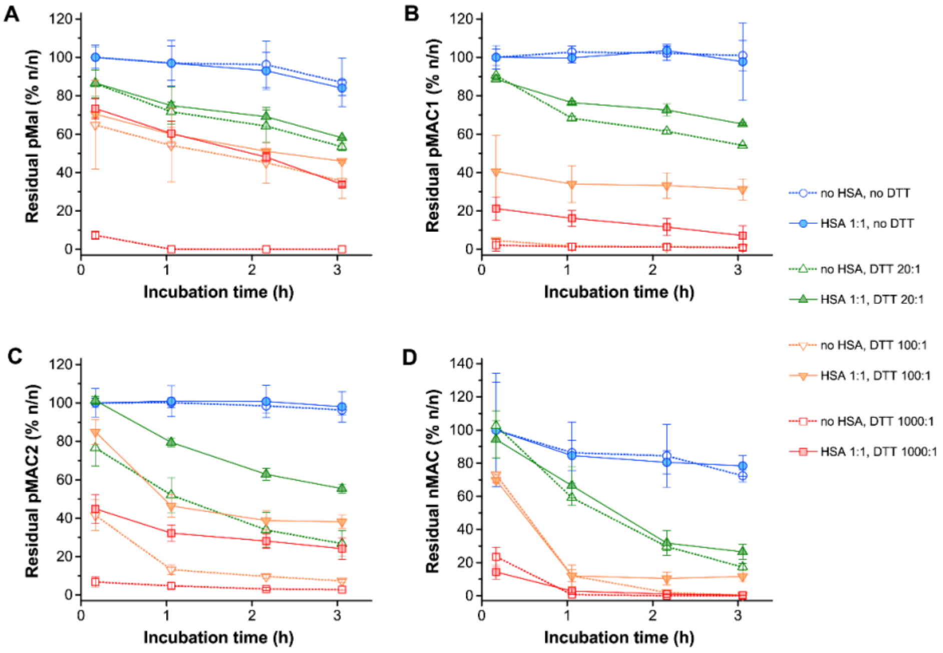

Supplementary Figure 23. Stability of pMal and MACs(10 µM in PBS/EtOH 75:25 v/v) as a function of incubation time, in the absence or in the presence of HSA and in different redox conditions (HSA and DTT concentrations expressed in molar ratio), as determined by HPLC- UV analysis. (A) pMal. (B) pMAC1. (C) pMAC2. (D) nMAC.

Stereoisomers of Ptx and Nlg

Ptx

paclitaxel

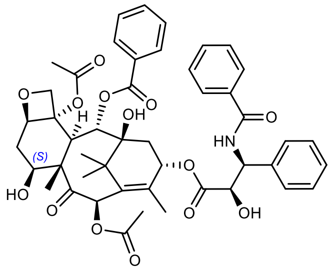

(*R*,*S*)-Nlg

(5*R*,2′*S*)-NLG919

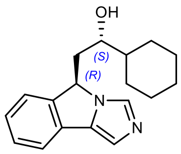

(*R*,*R*)-Nlg

(5*R*,2′*R*)-NLG919

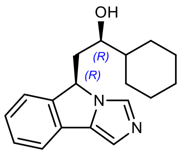

7-epi-Ptx

7-epipaclitaxel

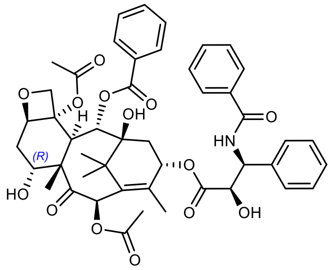

(*S*,*R*)-Nlg

(5*S*,2′*R*)-NLG919

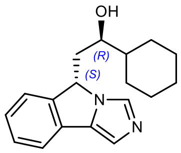

(*S*,*S*)-Nlg

(5*S*,2′*S*)-NLG919

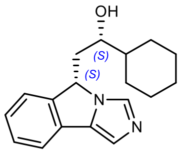

Supplementary Figure 24. Chemical structures of the main stereoisomers of Ptx and Nlg.

3.4 HPLC calibration for Ptx and Nlg

The quantification of Ptx and Nlg in drug release studies was performed using the optimized HPLC method reported in the Main Text. Calibration curves were obtained by linear regression from the values of cumulative peak area for the main stereoisomers of both analytes – Ptx and 7-epi-Ptx, (*R*,*S*/*S*,*R*)- and (*S*,*S*/*R*,*R*)-Nlg – as a function of quantity, expressed in picomoles. Samples for calibration were prepared in PBS/EtOH 75:25 v/v by addition of PBS pH 7.4 to EtOH solutions of increasing concentrations, obtained by dilution from highly concentrated stock solutions (5 mg/mL in DMSO for Ptx; 5 mg/mL in EtOH for Nlg).

Supplementary Table 1. Linear regression parameters and limits for the calibration of the HPLC method used for the quantification of Ptx and Nlg in drug release studies.

**Analyte**	**Ptx**	**Nlg**
**Equation**	$y = ax + b$	$y = ax + b$
$\boldsymbol{x}$	Quantity (pmol)	Quantity (pmol)
$\boldsymbol{y}$	Peak area (µV s) [λ = 228 nm]	Peak area (µV s) [λ = 271 nm]
**Samples (replicates, repeats)**	11 (2×2)	11 (2×2)
**Range**	0.3–585.5 pmol	0.9–1770.6 pmol
$\boldsymbol{a}$	1576.8	884.9
$\boldsymbol{b}$	−1895.7	−2021.8
$\boldsymbol{R^2}$	0.999905	0.999902
**LOD, pmol (Area)**	2.20 (1578)	6.77 (3965)
**LOQ, pmol (Area)**	6.61 (8526)	20.30 (15939)

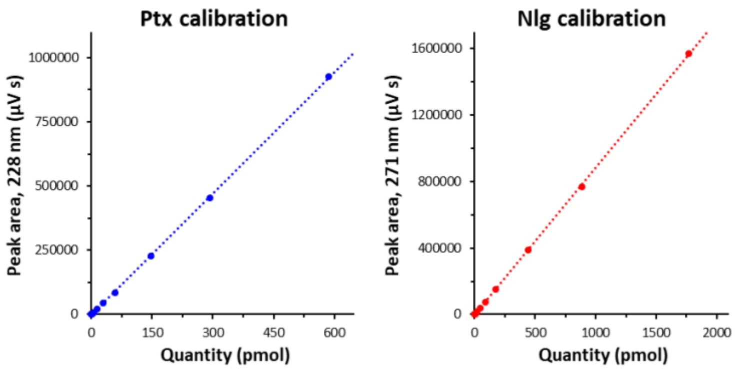

Supplementary Figure 25. Calibration curves for the HPLC-UV quantification of Ptx and Nlg in drug release studies.

3.5 Ptx and Nlg release from NB@5

Supplementary Table 2. Released quantities of Ptx and Nlg from NB@5 in PBS/EtOH 75:25 v/v, at 37 °C and in different redox conditions (DTT concentrations), as determined by HPLC analysis in drug release studies.

DTT 0 mM			DTT 1 mM			DTT 10 mM		
Dialysis time (h)	Ptx release (m/m)	Nlg release (m/m)	Dialysis time (h)	Ptx release (m/m)	Nlg release (m/m)	Dialysis time (h)	Ptx release (m/m)	Nlg release (m/m)
0.083	< LOQ	< LOQ	0.133	5.6 ± 0.3%	4.2 ± 0.4%	0.183	11.9 ± 0.7%	11.6 ± 0.7%
0.417	< LOQ	< LOQ	0.467	6.9 ± 0.4%	6.2 ± 0.4%	0.517	12.7 ± 0.7%	13.5 ± 0.8%
0.750	< LOQ	< LOQ	0.800	8.1 ± 0.6%	7.7 ± 0.5%	0.850	13.9 ± 1.6%	16.4 ± 1.9%
1.083	< LOQ	< LOQ	1.133	9.0 ± 1.0%	9.3 ± 0.9%	1.183	16.6 ± 1.1%	23.6 ± 1.7%
1.417	< LOQ	< LOQ	1.467	11.5 ± 0.7%	13.0 ± 0.8%	1.517	18.8 ± 1.1%	29.7 ± 1.8%
2.083	< LOQ	< LOQ	2.133	14.6 ± 1.3%	19.0 ± 1.8%	2.183	23.7 ± 1.4%	42.8 ± 2.7%
3.083	< LOQ	< LOQ	3.133	21.4 ± 1.4%	32.4 ± 2.0%	3.183	31.2 ± 1.8%	55.8 ± 3.5%
4.083	< LOQ	< LOQ	4.133	27.2 ± 2.5%	42.0 ± 3.9%	4.183	35.8 ± 2.5%	62.1 ± 4.4%
5.083	< LOQ	< LOQ	5.133	34.5 ± 2.0%	54.2 ± 3.4%	5.183	44.0 ± 2.6%	69.5 ± 4.4%
6.083	< LOQ	< LOQ	6.133	41.1 ± 2.4%	63.9 ± 4.0%	6.183	49.1 ± 2.9%	74.7 ± 4.6%
7.083	< LOQ	< LOQ	7.133	45.0 ± 2.6%	68.5 ± 4.3%	7.183	52.3 ± 3.1%	77.2 ± 4.8%
24.083	< LOQ	2.3 ± %	24.133	74.2 ± 04.3%	85.7 ± 5.3%	24.183	74.3 ± 4.3%	85.1 ± 5.3%
28.083	< LOQ	2.4 ± %	28.133	76.1 ± 04.4%	85.6 ± 5.3%	28.183	76.7 ± 4.5%	84.8 ± 5.3%
48.000	4.4 ± 0.3%	2.6 ± %	48.050	79.6 ± 04.7%	86.3 ± 5.3%	48.100	78.1 ± 4.6%	85.3 ± 5.3%

4 Nanobinders preparation and characterization

Supplementary Table 3. Experimental details for the preparation of MAC-devoid micelles.

Sample	pMal stock (mg/mL)	NlgD stock (mg/mL)	pMal/NlgD ratio (m/m)	Nlg/Ptx ratio (n/n)	Diameter (nm)	PDI
@pMal	10 (EtOH)	—	100:0	—	130.3 ± 2.1	0.151 ± 0.001
@NlgD	—	10 (EtOH)	0:100	—	120.8 ± 1.3	0.180 ± 0.001
@pMal-NlgD	10 (EtOH)	10 (EtOH)	40:60	3.91	152.0 ± 1.7	0.068 ± 0.001

Supplementary Table 4. Experimental details for the preparation of selected pMAC1-based NBs.

Sample	pMAC stock (mg/mL)	NlgD stock (mg/mL)	nMAC stock (mg/mL)	Nlg/Ptx ratio (n/n)	MAC loading (n/n)	Diameter (nm)	PDI
pMAC1@NB1	10 (DMF)	10 (EtOH)	—	0.49	67%	232.6 ± 15.1	0.331 ± 0.047
pMAC1@NB2	10 (DMF)	10 (EtOH)	—	0.33	75%	87.0 ± 5.6	0.489 ± 0.059
pMAC1@NB3	10 (DMF)	10 (EtOH)	—	0.82	55%	50.4 ± 7.1	0.365 ± 0.076
pMAC1@NB4	8 (DMF)	10 (EtOH)	8 (DMF)	0.47	80%	65.0 ± 11.3	0.479 ± 0.107

Supplementary Table 5. Experimental details for the preparation of selected pMAC2-based NBs.

Sample	pMAC stock (mg/mL)	NlgD stock (mg/mL)	nMAC stock (mg/mL)	pMAC/NlgD/nMAC ratio (m/m/m)[a]
pMAC2@NB1 (**NB@1**)	10 (DMF)	10 (EtOH)	—	85:15:0
pMAC2@NB2 (**NB@2**)	10 (DMF)	10 (EtOH)	—	70:30:0
pMAC2@NB3 (**NB@3**)	10 (DMF)	10 (EtOH)	8 (DMF)	40:40:20
pMAC2@NB4 (**NB@4**)	10 (DMF)	10 (EtOH)	8 (DMF)	40:50:10
pMAC2@NB5 (**NB@5**)	10 (DMF)	10 (EtOH)	8 (DMF)	40:30:30

[a] Ratios are expressed as mass (m) to mass.

Supplementary Table 6. Experimental details for the preparation of fluorescently labelled NB formulations.[a]

Sample	pMAC2 content (m/m)	NlgD content (m/m)	nMAC content (m/m)	pMal content (m/m)	NR content (m/m)	Nlg/Ptx ratio (n/n)	Ptx/NR ratio (n/n)	MAC loading (n/n)	pMAC2 loading (n/n)	Diameter (nm)
f-NB@5	37%	28%	28%	0%	8%	3.55	1.05	37%	14%	92.1 ± 1.8
f-NB@6	23%	54%	0%	16%	8%	4.39	1.25	7%	7%	92.2 ± 0.6
f-NB@7	0%	21%	45%	26%	8%	3.59	0.99	35%	0%	99.2 ± 2.3

[a] pMAC2 stock solution = 10 mg/mL in DMF; NlgD stock solution = 10 mg/mL in EtOH; nMAC stock solution = 8 mg/mL in EtOH; Nile red (NR) stock solution = 8 mg/mL in EtOH.

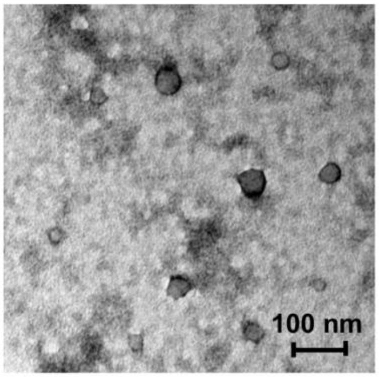

Supplementary Figure 26. TEM analysis of @pMal-NlgD pre-incubated with HSA (10:1, m/m). Scale bar 100 nm.

5 In vitro studies on TNBC cell monolayers

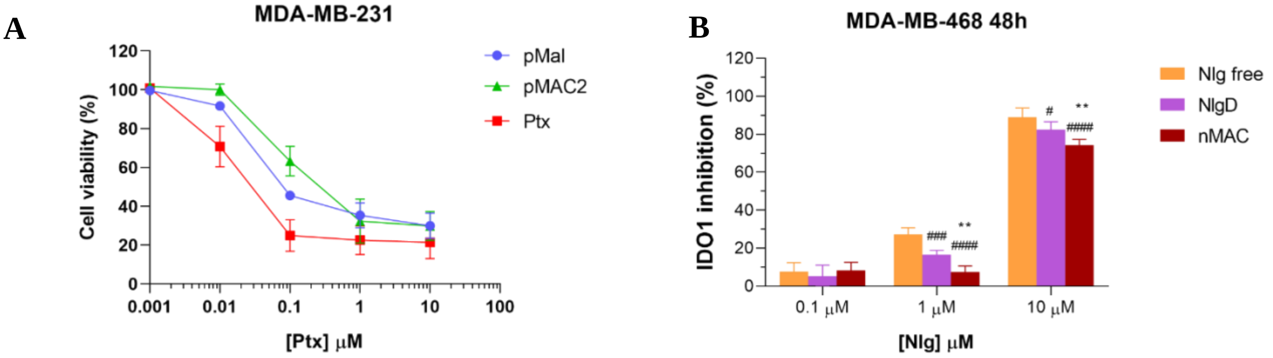

Supplementary Figure 27. (A) Viability curves of MDA-MB-231 cells exposed to increasing concentrations of Ptx and Ptx bioresponsive conjugates measured by MTS tests after 48 h of incubation. **(B)** IDO1 inhibition rate (%) in MDA-MB-468 cells incubated with the compounds for 48 h.

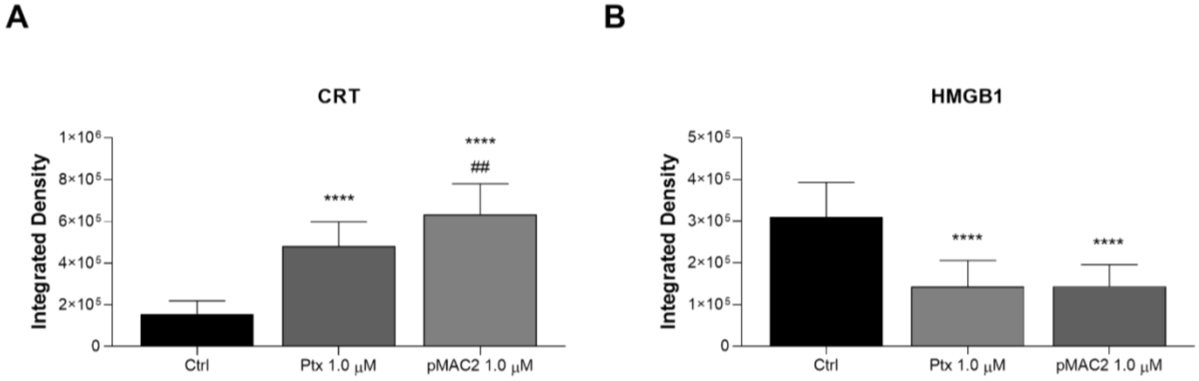

Supplementary Figure 28. Quantification of CRT (A) and HMGB1 (B) signal after 12 h of treatment of MDA-MB-468 with Ptx and pMAC2, respectively. Data are expressed as mean ± SD of at least 2 independent experiments carried out in triplicate and statistical analysis has been generated using the one-way ANOVA test, and Tukey's multiple comparison as a post-test. Results were considered statistically significant at p-values < 0.05 (** p-values < 0.01 and **** p-values < 0.0001). Data compared to Ctrl are reported as *, while data compared to Ptx 1.0 µM are indicated with #.

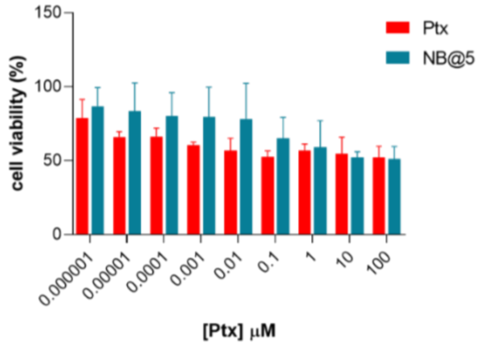

Supplementary Figure 29. Evaluation of cytotoxicity in MSCs cells exposed to drugs for 48 h.

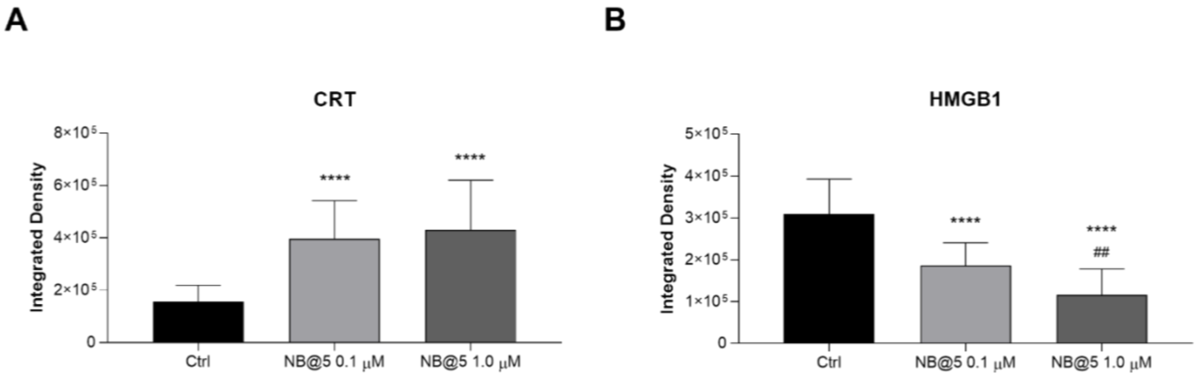

Supplementary Figure 30. Quantification of CRT (A) and HMGB1 (B) signals after 12 h of treatment of MDA-MB-468 at two different concentrations of Ptx in NB@5, 0,1 and 1 µM, respectively. Data are expressed as mean ± SD of at least 2 independent experiments carried out in triplicate and statistical analysis has been generated using the one-way ANOVA test, and Tukey's multiple comparison as a post-test. Results were considered statistically significant at p-values < 0.05 (** p-values < 0.01 and **** p-values < 0.0001). Data compared to Ctrl are reported as *, while data compared to NB@5 0.1µM are indicated with #.

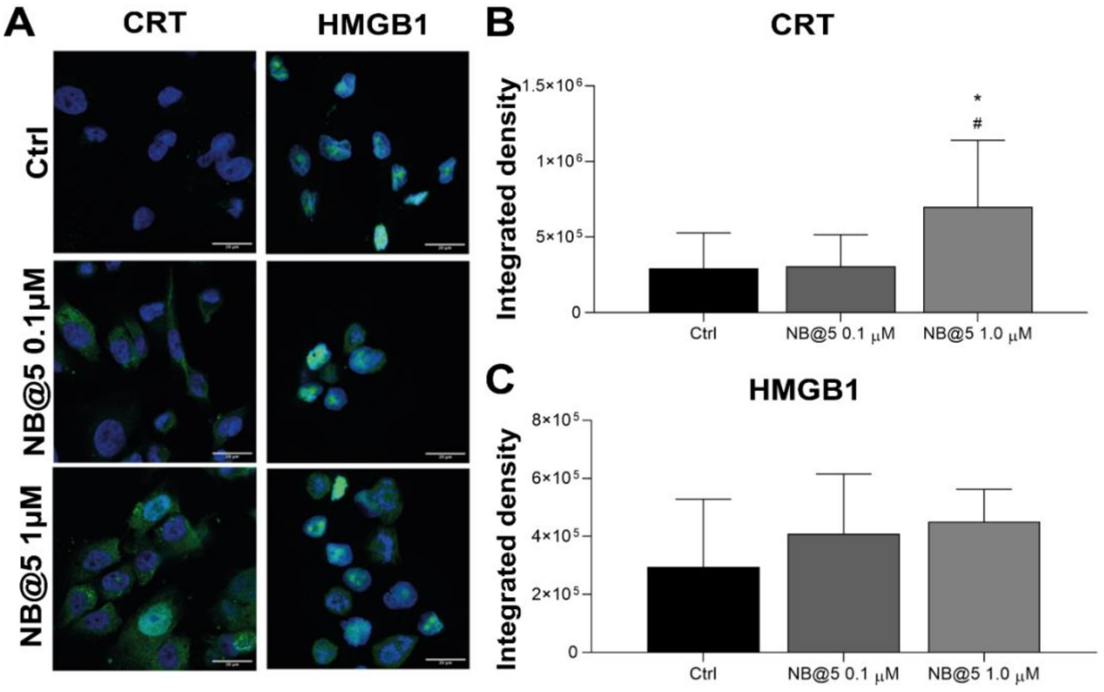

Supplementary Figure 31. (A) Immunofluorescence images of MDA-MB-231 cells incubated for 1 h with two different concentrations of Ptx in NB@5, 0,1 and 1 µM, respectively, and stained with antibody against CRT and HMGB1 FITC conjugated (green) and with Hoechst 33342 (blue) for nuclei recognition. Scale bar: 20 µm. (B) and (C). Quantification of CRT and HMGB1 signals, respectively. Data are expressed as mean ± SD of at least 2 independent experiments carried out in triplicate and statistical analysis has been generated using the one-way ANOVA test, and Tukey's multiple comparison as a post-test. Results were considered statistically significant at p values < 0.05 (* p-values < 0.05). Data compared to Ctrl are reported as *, while data compared to NB@5 0.1µM are indicated with #.

6 References

1. Wang Q, Guan J, Wan J, Li Z. Disulfide based prodrugs for cancer therapy. RSC Adv. 2020;10(41):24397–409.
2. Chen H, Wang G, Lang L, Jacobson O, Kiesewetter DO, Liu Y, et al. Chemical conjugation of evans blue derivative: A strategy to develop long-acting therapeutics through albumin binding. Theranostics. 2016;6(2):243–53.
3. Castañeda L, Wright ZVF, Marculescu C, Tran TM, Chudasama V, Maruani A, et al. A mild synthesis of N-functionalised bromomaleimides, thiomaleimides and bromopyridazinediones. Tetrahedron Lett. 2013;54(27):3493–5.
4. Suzuki T, Hisakawa S, Itoh Y, Suzuki N, Takahashi K, Kawahata M, et al. Design, synthesis, and biological activity of folate receptor-targeted prodrugs of thiolate histone deacetylase inhibitors. Bioorg Med Chem Lett. 2007 Aug 1;17(15):4208–12.
